# Supplementary material for: Probabilistic inference of the genetic architecture underlying functional enrichment of complex traits
Source: Nat Commun. 2021 Nov 30;12:6972. doi: 10.1038/s41467-021-27258-9 (PMC8633298; doi:10.1038/s41467-021-27258-9)
Supplement: Supplementary file 1 — Supplementary Information [file 41467_2021_27258_MOESM1_ESM.pdf]

Supplementary Information

Probabilistic inference of the genetic architecture underlying functional enrichment of complex traits

Marion Patxot, Daniel Trejo Banos, Athanasios Kousathanas, Etienne J. Orliac, Sven E. Ojavee, Gerhard Moser, Alexander Holloway, Julia Sidorenko, Zoltan Kutalik, Reedik Mägi, Peter M. Visscher, Lars Rönnegård, Matthew R. Robinson

Supplementary Tables

generative model	causal variant allocation	causal variants	effect size (b), LD (w), MAF (p) relationship
1	highest MAF per LD block	10,000	$b \propto N(0, w^{-0.25}[p(1-p)]^{-0.25})$
2	highest MAF per LD block	10,000	$b \propto N(0, w^{0.25}[p(1-p)]^{-0.25})$
3	highest MAF per LD block	10,000	$b \propto N(0, w^{-0.25}[p(1-p)]^{0.75})$
4	highest MAF per LD block	10,000	$b \propto N(0, w^{0.25}[p(1-p)]^{0.75})$
5	highest MAF per LD block	10,000	$b \propto N(0, w^{0}[p(1-p)]^{0})$
6	highest MAF per LD block	5,000	$b \propto N(0, w^{-0.25}[p(1-p)]^{-0.25})$
7	highest MAF per LD block	5,000	$b \propto N(0, w^{0.25}[p(1-p)]^{-0.25})$
8	highest MAF per LD block	5,000	$b \propto N(0, w^{-0.25}[p(1-p)]^{0.75})$
9	highest MAF per LD block	5,000	$b \propto N(0, w^{0.25}[p(1-p)]^{0.75})$
10	highest MAF per LD block	5,000	$b \propto N(0, w^{0}[p(1-p)]^{0})$
11	random	10,000	$b \propto N(0, w^{-0.25}[p(1-p)]^{-0.25})$
12	random	10,000	$b \propto N(0, w^{0.25}[p(1-p)]^{-0.25})$
13	random	10,000	$b \propto N(0, w^{-0.25}[p(1-p)]^{0.75})$
14	random	10,000	$b \propto N(0, w^{0.25}[p(1-p)]^{0.75})$
15	random	10,000	$b \propto N(0, w^{0}[p(1-p)]^{0})$
16	random	5,000	$b \propto N(0, w^{-0.25}[p(1-p)]^{-0.25})$
17	random	5,000	$b \propto N(0, w^{0.25}[p(1-p)]^{-0.25})$
18	random	5,000	$b \propto N(0, w^{-0.25}[p(1-p)]^{0.75})$
19	random	5,000	$b \propto N(0, w^{0.25}[p(1-p)]^{0.75})$
20	random	5,000	$b \propto N(0, w^{0}[p(1-p)]^{0})$

Supplementary Table 1. The generative genetic models used in the simulation study. Imputed SNP marker data from chromosomes 19, 20, 21 and 22 of 40,000 randomly selected UK Biobank participants were selected, giving 596,741 markers in total. Marker effects were simulated according to the 20 generative models in two ways: (i) a single distribution of marker effects, and (ii) 13 distributions of marker effects for 13 different genomic annotation groups with different proportions of SNP heritability (h^2_{SNP}) explained for exonic variants ($h^2_{SNP} = 0.1$), intronic variants ($h^2_{SNP} = 0.2$), 1kb promotor variants ($h^2_{SNP} = 0.05$), 1-10kb enhancer variants (0.025), 1-10kb transcription factor binding sites ($h^2_{SNP} = 0.025$), 1-10kb other variants ($h^2_{SNP} = 0$), 10-500kb enhancers ($h^2_{SNP} = 0.05$), 10-500kb transcription factor binding sites ($h^2_{SNP} = 0.05$), 10-500kb other variants ($h^2_{SNP} = 0$), 500kb-1Mb enhancers ($h^2_{SNP} = 0.05$), 500kb-1Mb transcription factor binding sites ($h^2_{SNP} = 0.05$), 500kb-1Mb other variants ($h^2_{SNP} = 0$),and other non-annotated SNPs ($h^2_{SNP} = 0$). 10 simulation replicates were created for both (i) and (ii) giving a total set of 400 simulated phenotypes.

Supplementary Figures

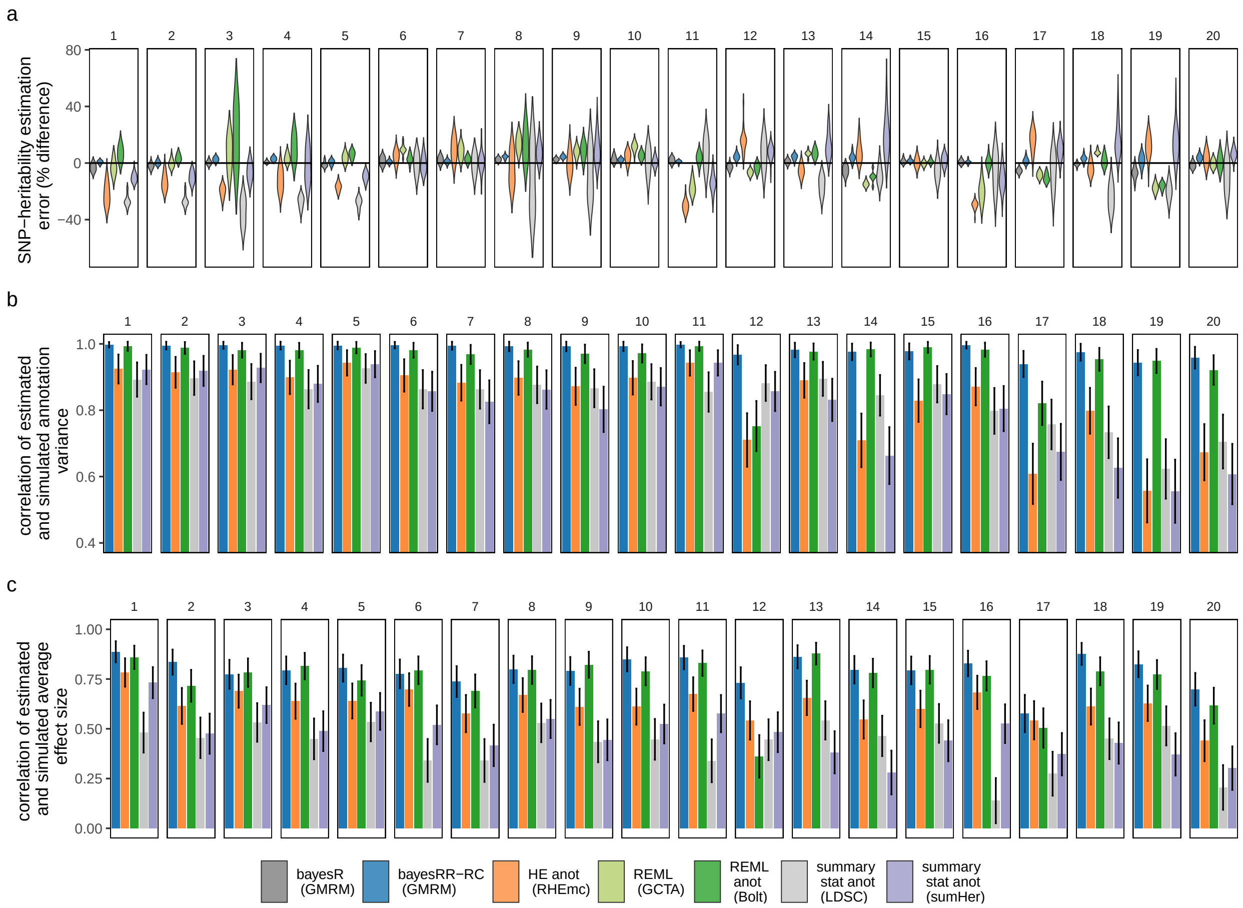

Supplementary Figure 1. Simulation study of the variance component estimation performance of BayesRR-RC implemented in GMRM. (a) Violin-plot of the genome-wide SNP-heritability estimates as a percentage difference from the simulated value for 40 replicates, for each of 20 different generative genetic models described in Table S1. For each generative genetic model we compare seven different statistical models: a mixture of regression model with a single global variance component known as "bayesR" implemented in our GMRM software (bayesR GMRM), the mixture of regression model with multiple group-specific variance components described in this work (bayesRR-RC GMRM), Haseman-Elston regression with annotation-specific relationship matrices implemented in the RHEmc software (HE anot RHEmc), a single component REML model implemented in the software GCTA (REML GCTA), a multiple group-specific variance component REML model implemented in the software bolt (REML anot Bolt), and two annotation summary statistic models implemented in the software LDSC and sumHer. (b) The correlation of the estimated genetic variance for each of 13 genetic annotation groups and the simulated genetic variance across the 40 replicates, for each of the five statistical approaches which enable annotation-specific estimation. (c) Bar-plots of the correlation of the estimated and simulated average effect size of each annotation across simulation replicates. Error bars give the SD.

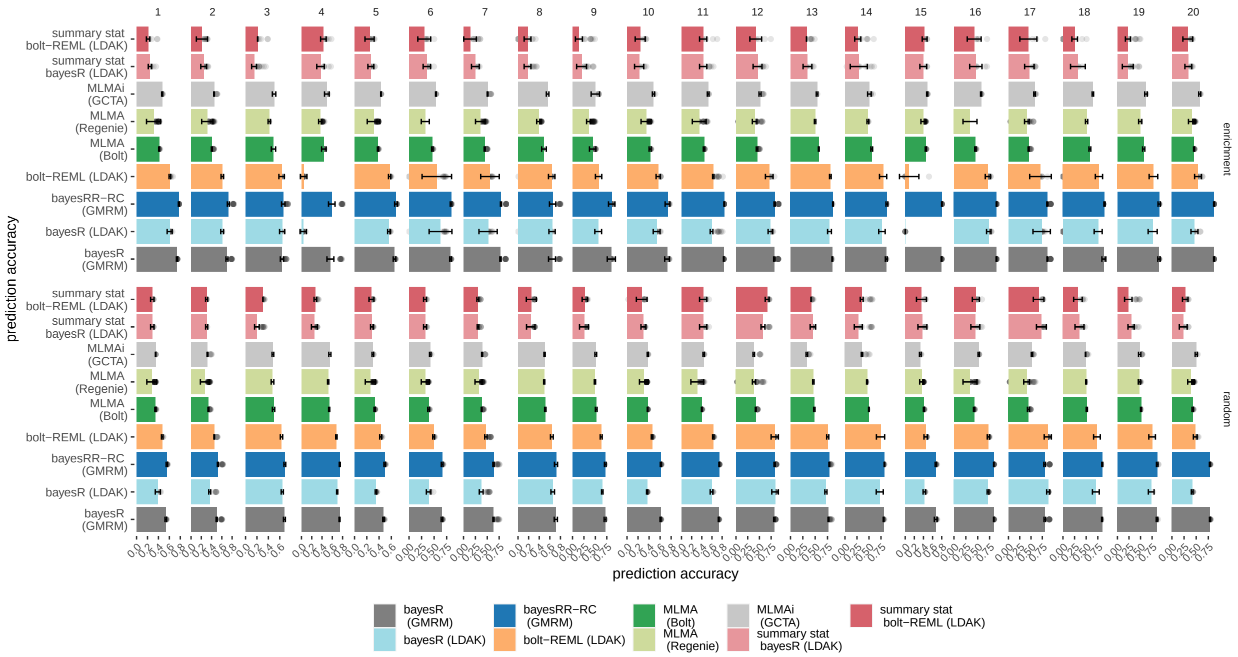

Supplementary Figure 2. Simulation study of the prediction performance of BayesRR-RC implemented in GMRM. Average prediction accuracy in an independent sample, defined as the squared correlation of the predicted and simulated genetic value, with error bars giving the SD. For each of the 20 different generative genetic models described in Supplementary Table 1, we compare the prediction accuracy obtained in a testing set of 10,000 unrelated individuals from the UK Biobank, selected at random and unrelated to the training data. We predicted simulated phenotypes using SNP marker effect sizes obtained from nine different statistical methods: bayesR implemented in our GMRM software (bayesR GMRM); the mixture of regression model with multiple group-specific variance components described in this work (bayesRR-RC GMRM); three frequentist mixed-linear association models (MLMA) where the genetic marker tested for association is removed from the relationship matrix (implemented in software Bolt and Regenie), or fitted both as fixed and random (MLMAi implemented in the software GCTA); and four MegaPRS models using genomic annotation SNP variance estimates from SumHer and implemented in the software LDAK: (i) an individual-level bayesR model (bayesR LDAK), (ii) an individual-level boltREML model (bolt-REML LDAK), (iii) a summary statistic bayesR model (summary stat bayesR LDAK) and (iv) a summary statistic boltREML model (summary stat bolt-REML).

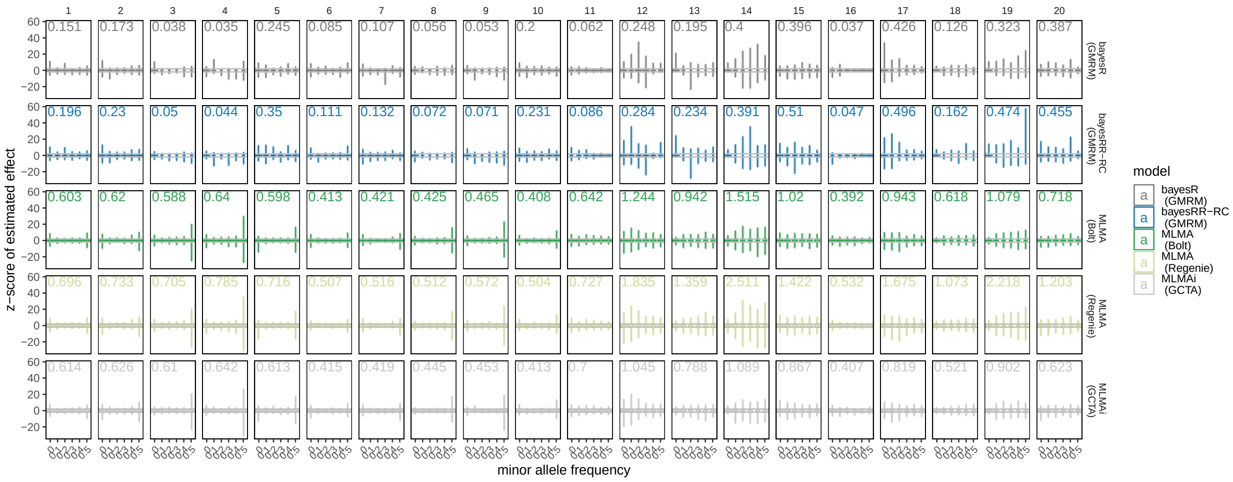

Supplementary Figure 3. Simulation study of the effect size estimation of BayesRR-RC implemented in GMRM. For each of the 20 different generative genetic models described in Supplementary Table 1, we compare model performance of our approach (bayesRR-RC GMRM) to bayesR implemented in our GMRM software (bayesR GMRM) and frequentist mixed-linear association models (MLMA) where the genetic marker tested for association is removed from the relationship matrix (implemented in software Bolt and Regenie), or fitted both as fixed and random (MLMAi implemented in the software GCTA). For bayesR (GMRM) and bayesRR-RC (GMRM), we summed the squared regression coefficient estimates of all SNPs in LD with each causal variant (markers in LD $R^2 \geq 0.1$ within 1MB), took the posterior mean, and calculated the z-score from the simulated value. For the MLMA approaches, we calculated the z-score of the causal marker estimate from the simulated value. Violin plots for groups of minor allele frequency of the causal variant are shown, with values giving the variance in each facet.

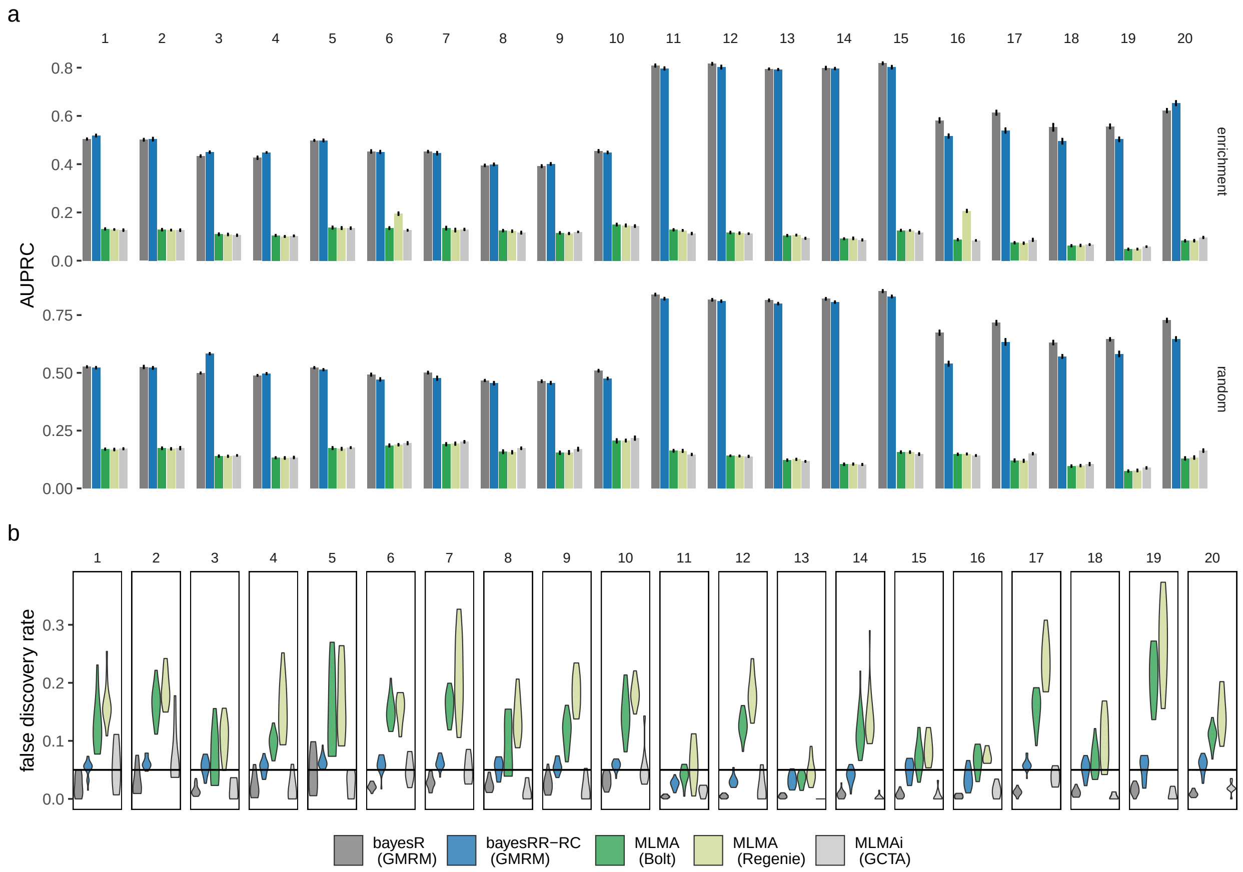

Supplementary Figure 4. Simulation study of the effect size localization of BayesRR-RC implemented in GMRM. (a) For each of the 20 different generative genetic models described in Table S1, we compare the area-under the precision-recall curve (AUPRC) for bayesRR-RC (described in this work and implemented in GMRM), bayesR (implemented in GMRM) and mixed-linear association models (MLMA). For Bayesian methods bayesR (GMRM) and bayesRR-RC (GMRM), we use our PPWV metric (see Methods), with true positives defined as LD blocks that contain a causal variant and false positives defined as LD blocks that did not contain a causal variant. For MLMA methods implemented in GCTA (MLMAi GCTA), Bolt (MLMA Bolt) and Regenie (MLMA Regenie), we LD-clumped the results (LD $R^2 \geq 0.01$) using the p-value of the chi-squared statistics. Markers in $R^2 \geq 0.01$ with simulated causal variants were defined as true positives and those not in LD $R^2 \geq 0.01$ as false positives. (b) False discovery rate (FDR), with the line giving the 5% threshold. For the MLMA methods, FDR was calculated as the proportion of LD independent SNPs with p-value $\leq 5\text{x}10^{-8}$ that were not in LD $R^2 \geq 0.01$ with causal variants. For the Bayesian methods, we defined FDR as the proportion of LD blocks with posterior probability of window variance (PPWV), of $\geq 95\%$ at 0.001% variance threshold that did not contain a causal variant.

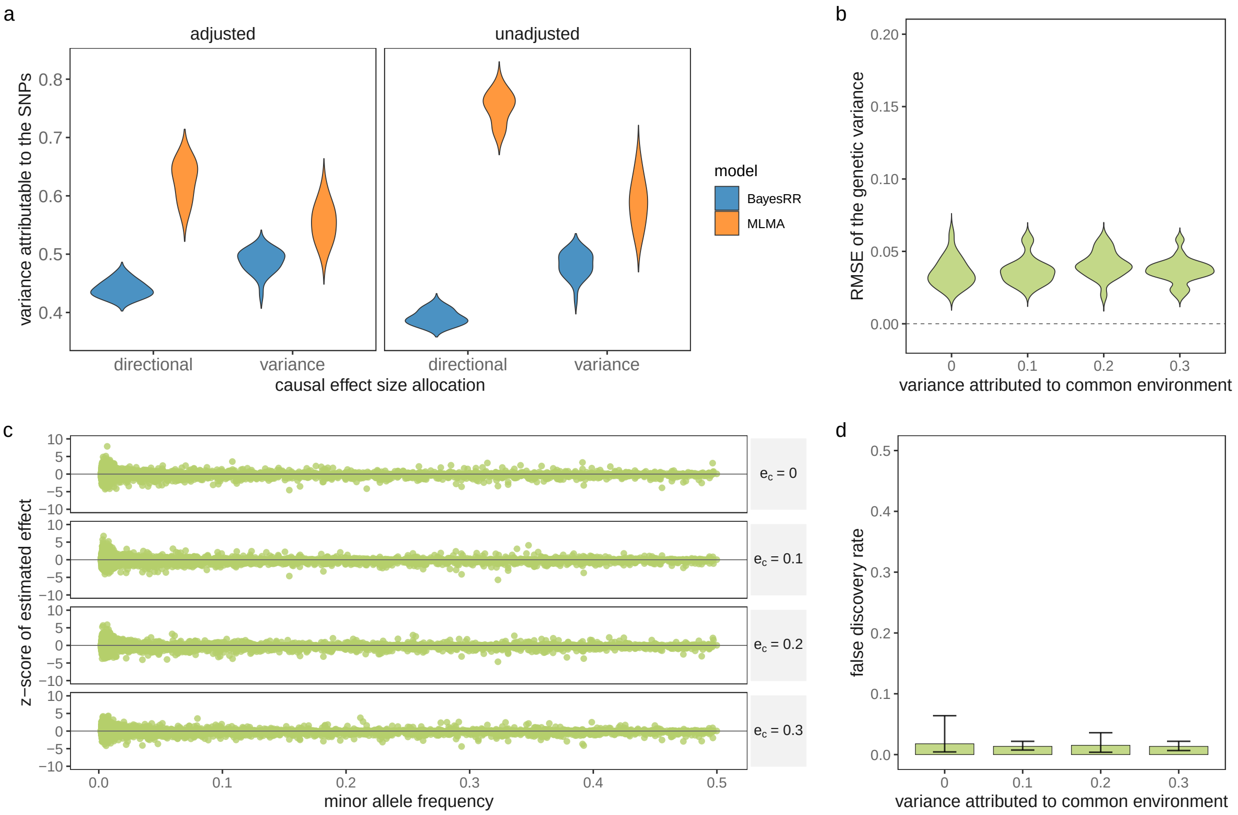

Supplementary Figure 5. Exploring effects of population stratification and relatedness among samples. (a) Simulation study using real genomic data from chromosome 22 where 10,000 individuals were selected from 2 UK Biobank assessment centres (Glasgow and Croydon). First, causal variants were allocated to 5000 high-LD SNPs with effect sizes simulated from a normal distribution with variance proportional to the F_{ST} among the two populations at each SNP (labelled 'variance', see Methods). Second, we selected the same high-LD SNPs as the causal variants, but simulated effect sizes to have correlation 0.5 with the allele frequency differences of the SNPs among the two populations, and thus not only is the effect size proportional to the F_{ST}, but there is also directional differentiation (trait increasing loci tend to be those with higher allele frequency in Croydon, trait decreasing alleles have lower frequency in Croydon). For each of these two scenarios, we simulated 50 replicate phenotypes where the phenotypic variance attributable to the causal SNPs is 0.5, there is a phenotypic difference where Croydon individuals have a phenotype that is on average 0.5 SD higher than Glasgow individuals (contributing variance 0.05), and residual variance was simulated from a normal with variance 0.45, to give a phenotype with mean of zero and variance of 1. The distribution across simulations of the estimated phenotypic variance attributable to the SNP markers is shown for each of the two causal effect size allocation scenarios when the data was analysed using a mixed-linear model association (MLMA, distribution of the point estimates) and a grouped Bayesian dirac spike and slab models (BayesRR, distribution of the posterior means). In the analysis, we either adjusted the phenotype by the first 20 PCs of the genetic data used in the simulation study ("adjusted") or we did not adjust the phenotype for the PCs ("unadjusted"). (b), (c) and (d) show BayesRR-RC simulation results using real genomic data from chromosome 21 and 22 and 10,000 families randomly selected from the UK Biobank. We simulated 20 replicates where we selected 2000 LD blocks at random, with an LD block defined as a group of SNP markers with squared LD correlation of at least 0.15. We assigned a causal SNP per LD block and for each replicate, we simulated 4 phenotypes increasing the variance attributed to family effects from 0 (no common environment) to 0.3 (see Methods). (b) Violin-plot of the root mean square error (RMSE) of the SNP-heritability estimates across simulation replicates. (c) For each LD block of each simulation replicate, we summed the squared regression coefficient estimates of all SNPs in the block and took the posterior mean. We then calculated the z-score of the LD block and plotted it against the minor allele frequency of the causal variant of the block. (d) Shows mean and 95% credible intervals of the false discovery rate defined as the posterior probability of window variance (PPWV), of $\geq 95\%$ at 0.001% variance threshold that did not contain a causal variant.

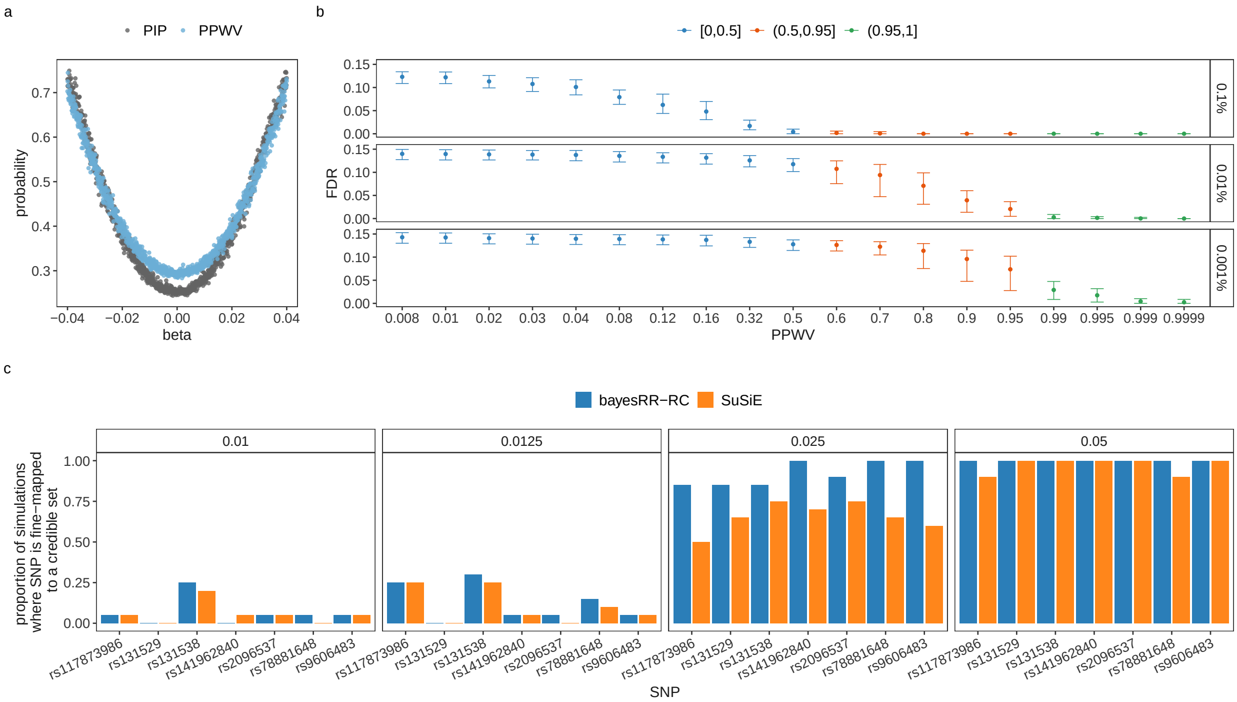

Supplementary Figure 6. Posterior inclusion probability (PIP) and posterior probability of window variance (PPWV).(a) We validate the use of PPWV in simulation study, first simulating 500 replicate data sets of 10,000 SNP markers for 5,000 individuals for each of two scenarios. In the first scenario, 1000 SNPs are randomly selected to be causal variants and all 10,000 SNP markers are LD independent. In the second, the 1000 causal variants are each in LD with four other variants with LD = 0.95, with the remaining 5000 variants having zero effect size and LD = 0. For each scenario, we simulate effect sizes as an equally spaced sequence from an effect size of -0.04 SD, to 0.04 SD giving genetic variance of 0.55, and we simulate residual variance from a normal distribution with zero mean and variance 0.45, to give a phenotype with zero mean and unit variance. For the first scenario, we calculate the posterior inclusion probability of each causal SNP. For the second scenario, we calculate the PPWV for each 5-SNP group. Across the 500 replicates, we take the mean PIP for each SNP of the 1000 different effect sizes for the first scenario and the mean PPWV of each of the 1000 5-SNP windows for the second scenario, and these are the points on the figure. (b) Shows mean and 95% credible interval of the false discovery rate (FDR), defined as the proportion of regions identified that do not contain a causal variant, at PPWV thresholds ranging from 0.8% to 100%. Here, we grouped SNPs in 50kb regions and selected the number of regions that explain at least 0.1%, 0.01% and 0.001% of the variance attributed to all SNP markers in 0.8% to 100% of the iterations using simulated data for chromosome 22 in the UK Biobank (see Methods). We compare the FDR at these different PPWV thresholds and as we lower the PPWV variance, the number of false discoveries in the model increases, but remains at $\leq$ 5% at PPWV $\geq$ 95%. (c) A comparison of BayesRR-RC and SuSiE where we assigned effect sizes of either 0.05, 0.025, 0.0125, or 0.01 on the SD scale to seven SNPs. For BayesRR-RC, we calculate the PPWV of the LD blocks containing the seven focal SNPs, and then prune these blocks based on the LD among the markers in the block to identify a credible set with LD $R^2 \geq 0.9$. We then count the proportion of times across 20 simulation replicates that each causal variant was contained with one of the credible sets. For SuSiE, we calculate the proportion of times that the credible sets identified contained one of the seven causal variants.

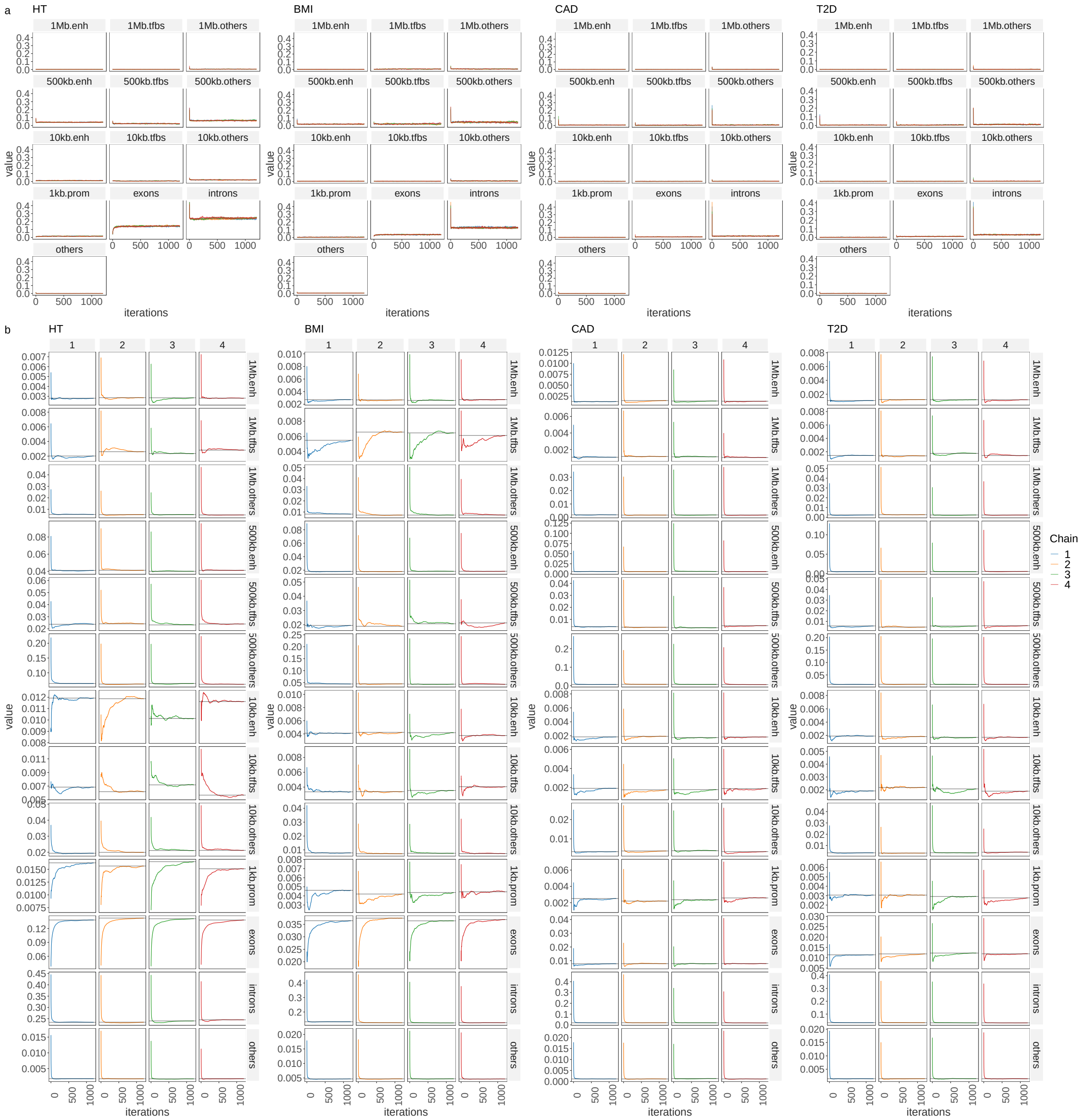

Supplementary Figure 7. Convergence diagnostics of model chains for UK Biobank analysis. (a) Traceplot of the phenotypic variance attributable to SNP markers for each trait across functional annotation of exonic regions, intronic regions, promoters (prom) 1kb upstream of coding regions, enhancers (enh) 1kb to 10kb upstream of coding regions, transcription factor binding sites (tfbs) 1kb to 10kb upstream of coding regions, other snps 1kb to 10kb upstream of coding regions, enh 10kb to 500kb upstream, tfbs 10kb to 500kb upstream, other snps 10kb to 500kb upstream, enh 500kb to 1MB upstream,tfbs 500kb to 1Mb upstream, other snps 500kb to 1Mb upstream and SNP markers elsewhere in the genome (other), with colours representing the different chains. (b) A time series of the running mean of each chain, for each annotation group and each trait showing all chains approach the same mean value for each parameter.

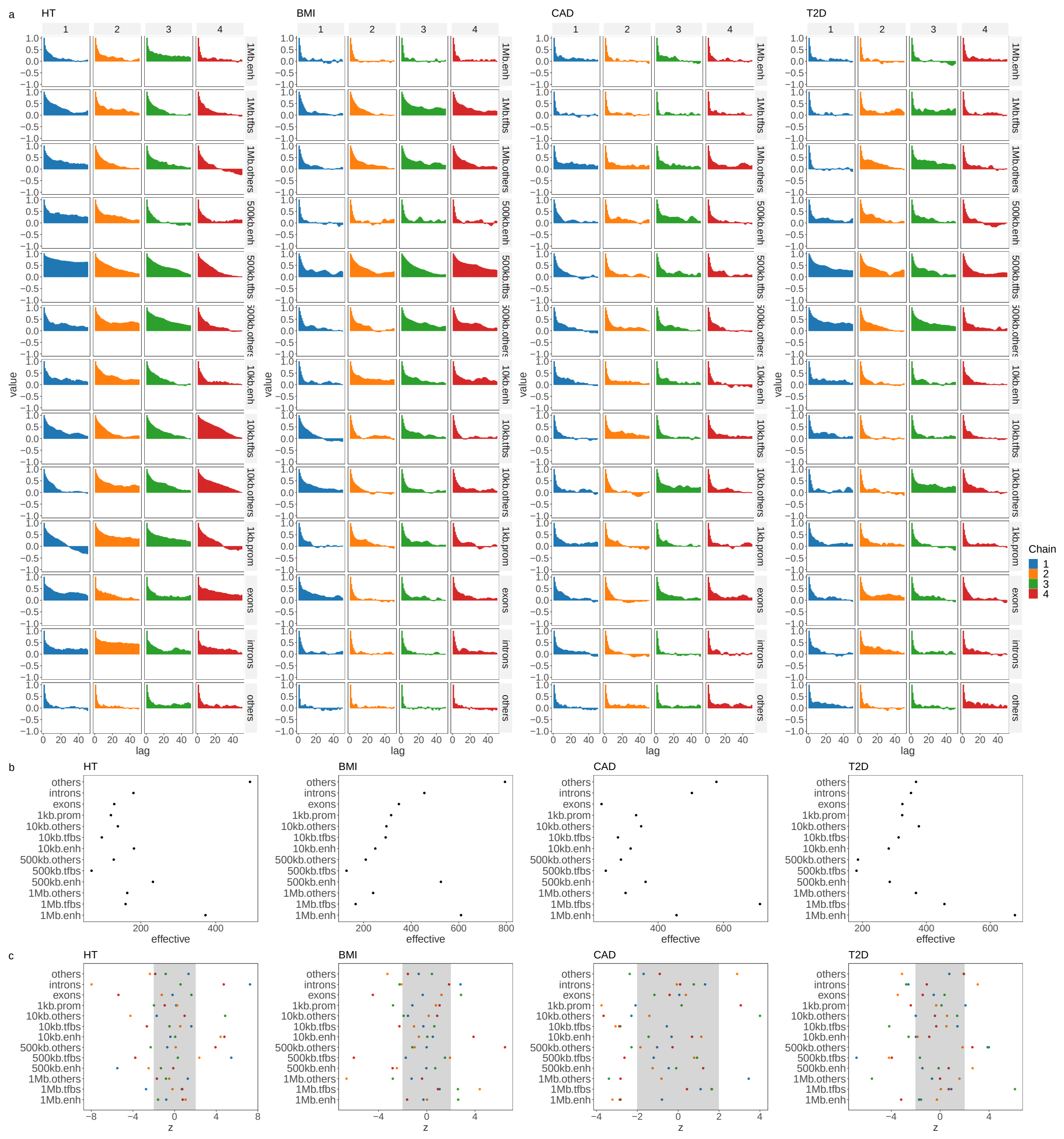

Supplementary Figure 8. Convergence diagnostics of model chains for UK Biobank analysis. (a) Lagged autocorrelation plot of each chain, for each annotation group and each trait and (b) Effective number of uncorrelated sampled obtained for each annotation group and each trait. As phenotypic variance is being partitioned it is not expected that posterior estimates obtained are entirely uncorrelated. (c) Geweke z-score statistic comparing the initial part of the chain to the final part, for each annotation group and each trait.

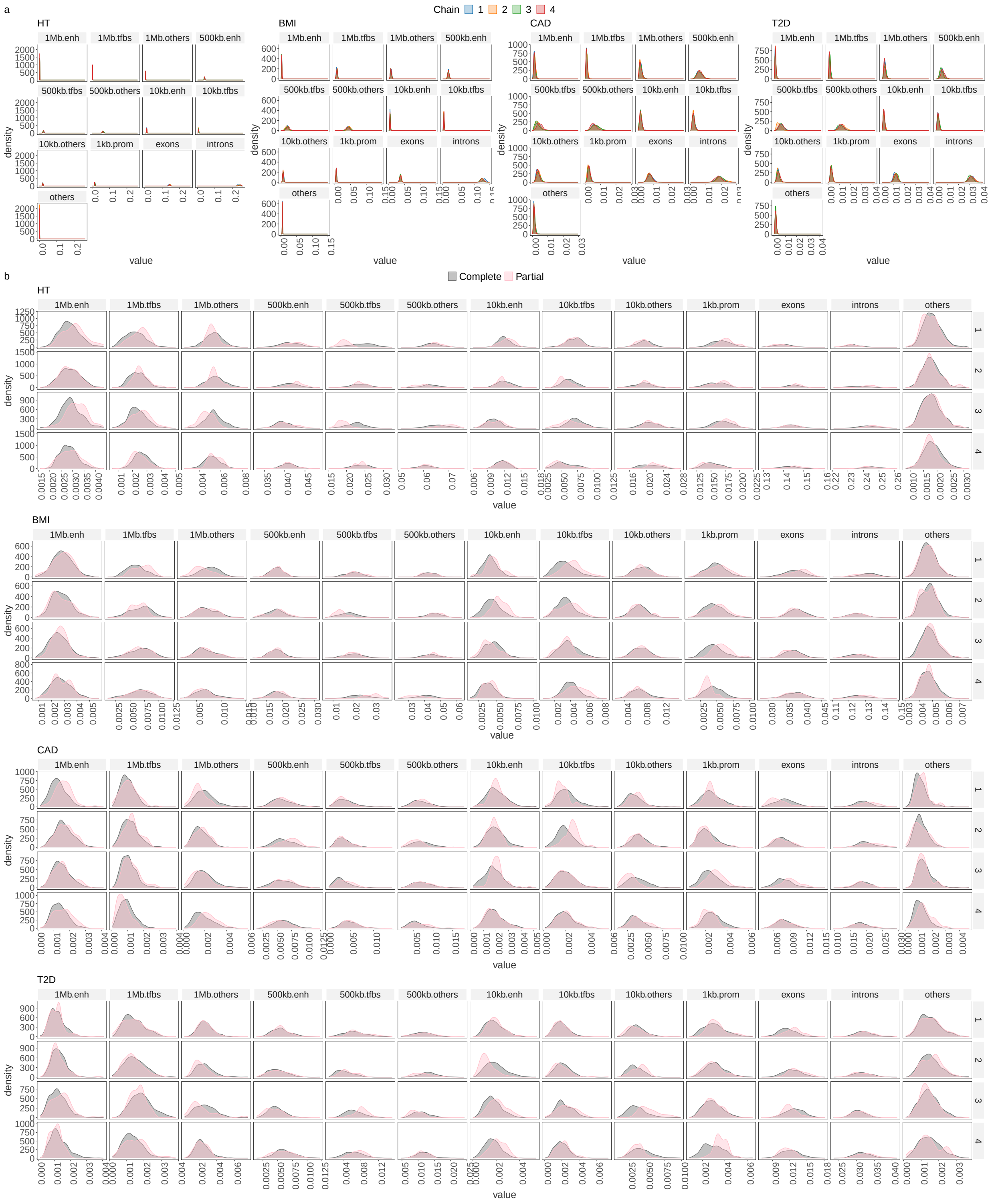

Supplementary Figure 9. Convergence diagnostics of model chains for UK Biobank analysis. (a) Overlapped density plots to compare the target distribution by chain showing each chain has converged in a similar space, for each annotation group and each trait. (b) Overlapped density plots comparing the last 10 percent of the chain (green), with the whole chain (pink), showing that the initial and final parts of the chain are sampling the same target distribution for each annotation group and each trait.

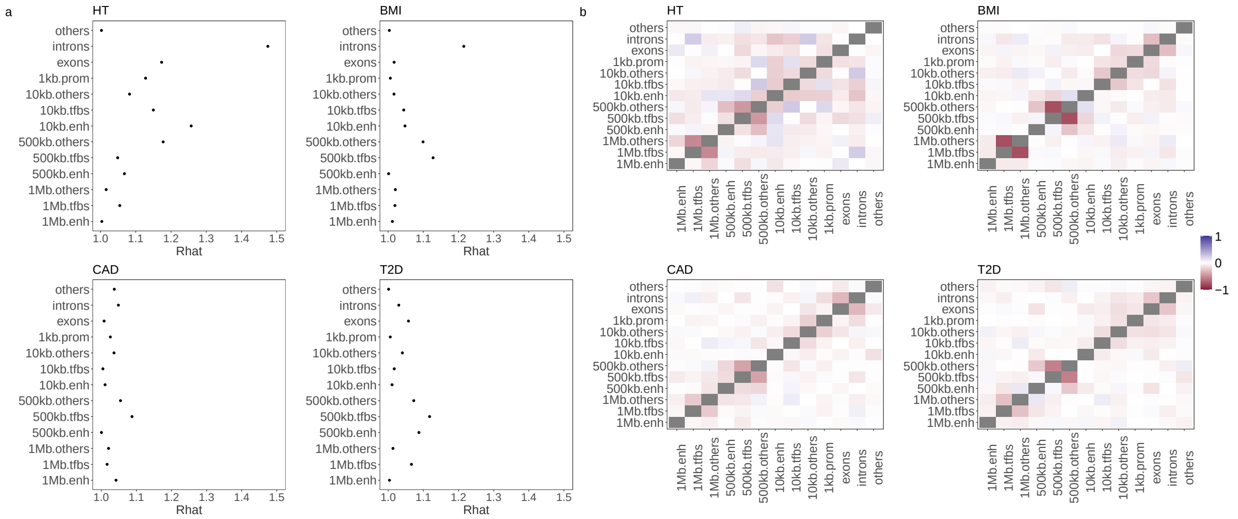

Supplementary Figure 10. Convergence diagnostics of model chains for UK Biobank analysis. (a) The potential scale reduction factor comparing the among- and within-chain variance for each annotation group and each trait. (b) The cross-correlation between all parameters for each annotation group and each trait.

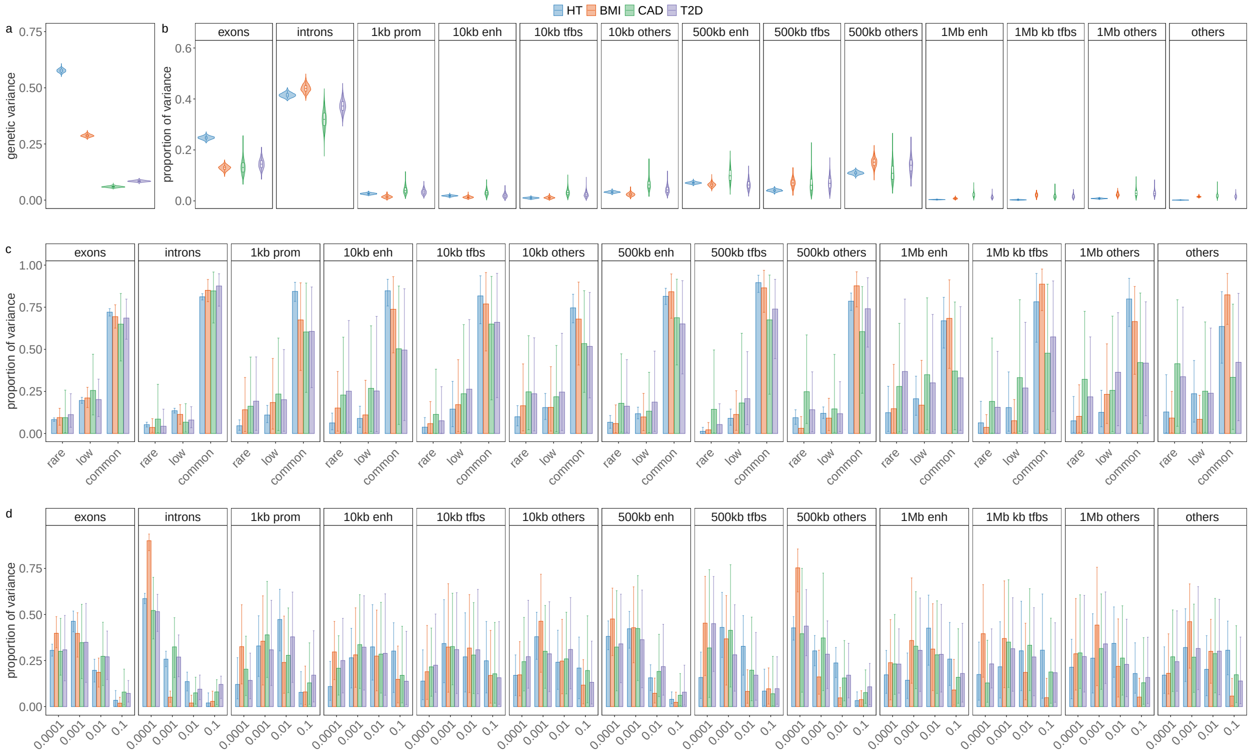

Supplementary Figure 11. Genetic architecture of height, body-mass-index (BMI), cardiovascular disease (CAD) and type-2-diabetes (T2D).(a) Shows violin plots with boxplots giving the 95% credible intervals for the posterior mean of the phenotypic variance attributable to the SNP markers in each trait. We find that SNPs contribute 57.66% (95%CI 56.09, 59.14) for height, 28.74% (95%CI 27.62, 30.00) for BMI, 5.94% (95%CI 5.30, 6.67) for CAD and 8.45% (95%CI 7.83, 9.18) for T2D. Values are summed over annotation, MAF and LD groups. (b) Violin plot with boxplots giving the 95% credible intervals of the proportion of the total genetic variance attributable to each annotation group. Values are summed over MAF and LD groups. All four traits show the same pattern of annotation-specific genetic variance, with main contributions from intronic regions, exonic regions, and SNPs located 10kb to 500kb upstream of genes to the genetic variance in the population. (c) Bar plots with error bars giving the 95% credible intervals for the proportion of variance of each annotation group that is attributable to each of the three MAF groups (rare, low, common) for each trait. Values are summed over LD groups. (d) Bar plots with error bars giving the 95% credible intervals for the proportion of variance of each annotation group that is attributable to each of the four non-zero mixtures (0.0001, 0.001, 0.01, 0.1) for each trait. Values are summed over MAF and LD groups. Within each annotation, variation is (c) attributable predominantly to variants with MAF>0.05 and (d) attributable predominantly to small (0.0001) to moderate (0.001) effect sizes variants with little differences across traits, except for BMI which has higher polygenicity compared to height, CAD and T2D. Posterior summary of n = 6,000 iterations with a thin of 5 and burn-in of 500 for each trait in all panels.

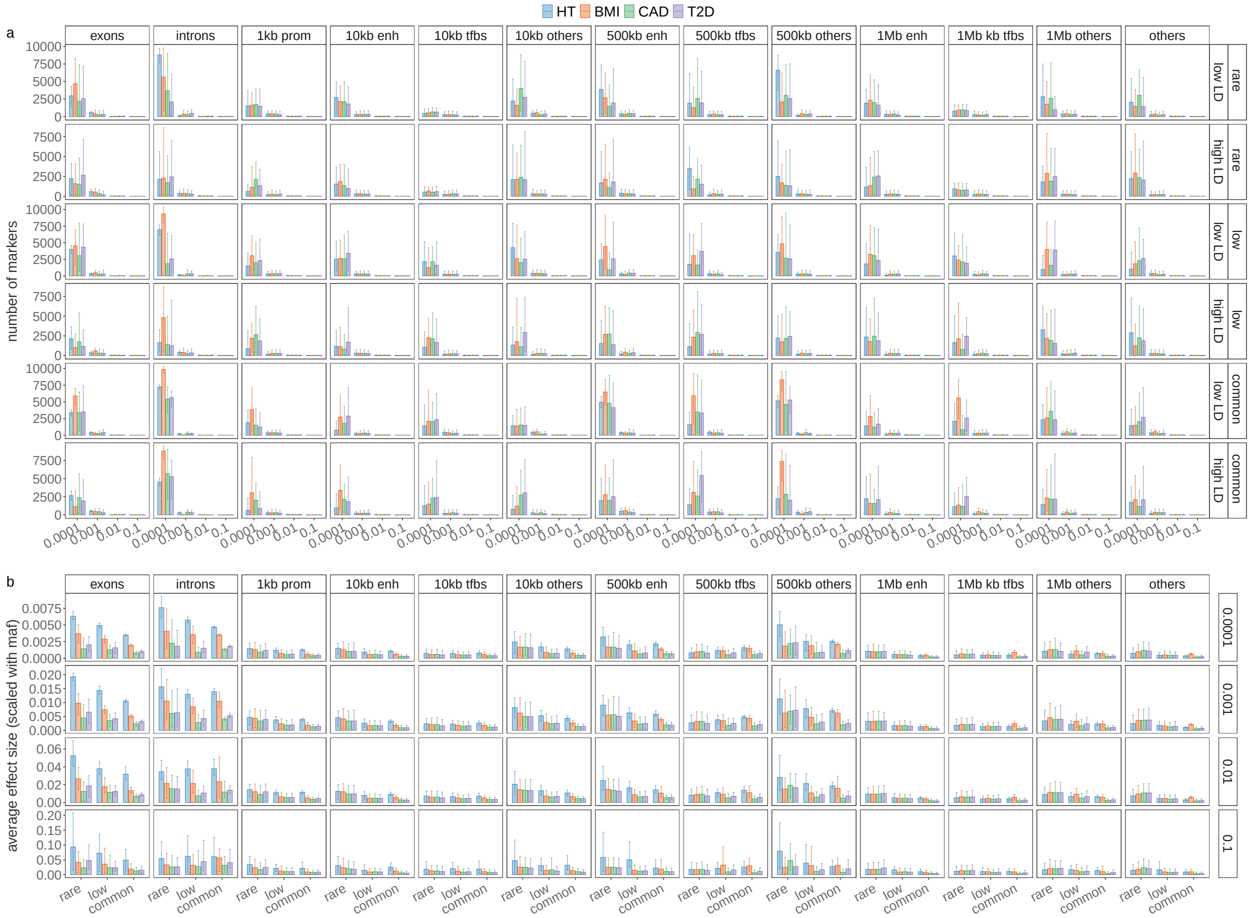

Supplementary Figure 12. Marker inclusion and effect estimate overview. (a) Bar plots of the number of markers entering the model for each mixture group (x-axis), within each MAF-LD group (y-axis facets, with top row MAF and bottom row LD), within each annotation (x-axis facets). Mixture 1 = 0.0001, 2 = 0.001, 3 = 0.01, 4 = 0.1. (b) Bar plots of the average effect size of markers in the model for each annotation group, scaling the effects to their frequency and split by mixture. Posterior summary of n = 6,000 iterations with a thin of 5 and burn-in of 500 for each trait in (a) and (b). Error bars give the 95% credible intervals in both panels.

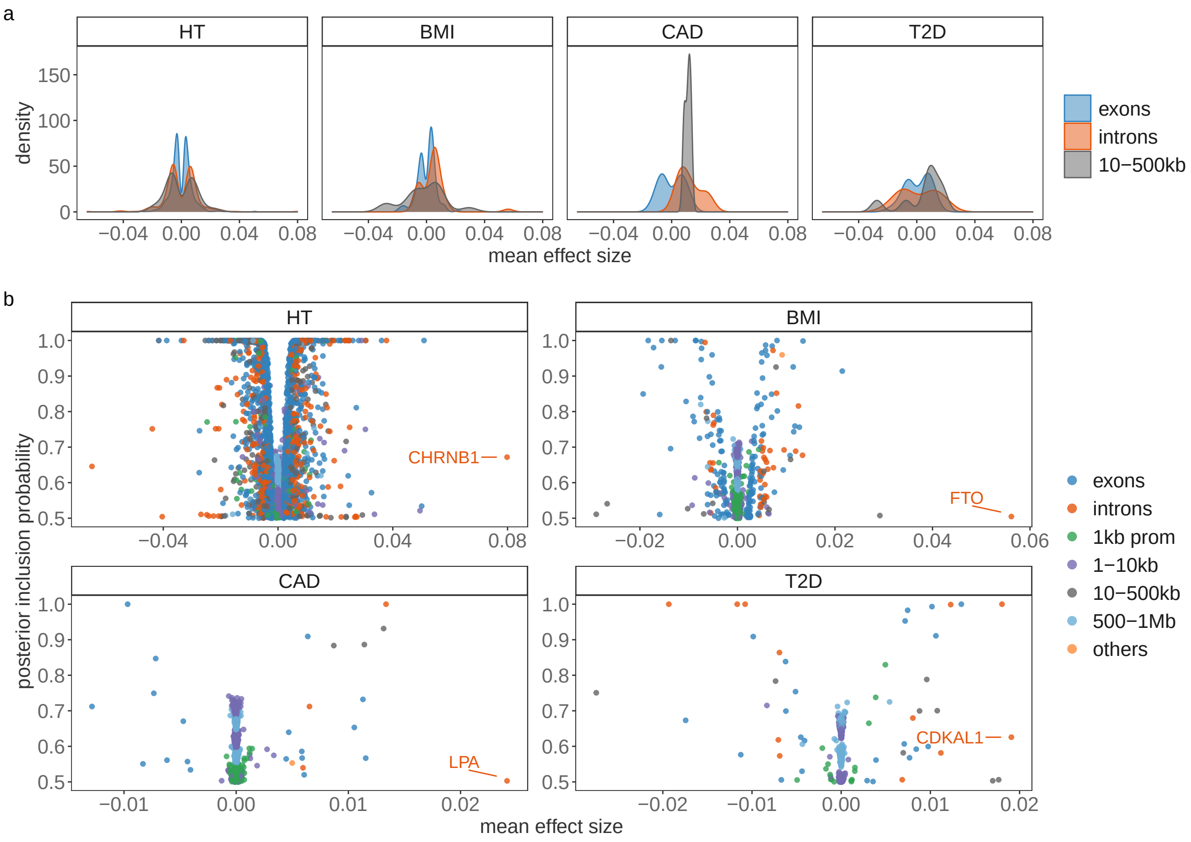

Supplementary Figure 13. Contribution of SNPs with posterior inclusion probability (PIP) > 0.5 to height, body-mass-index (BMI), cardiovascular disease (CAD) and type-2-diabetes (T2D). (a) Shows the distribution of mean effect sizes for SNPs with PIP > 0.5 attributed to exons, introns and 500kb upstream of genes in each trait. (b) We then plot the relationship between mean effect size and posterior inclusion probability for SNPs with PIP > 0.5 attributed to the annotation groups (exons, introns, SNPs located 1kb, 1-10kb, 10-500kb and 500-1Mb upstream of genes and other un-mapped SNPs). We labelled the closest gene to the SNP with the highest mean effect size in each trait.

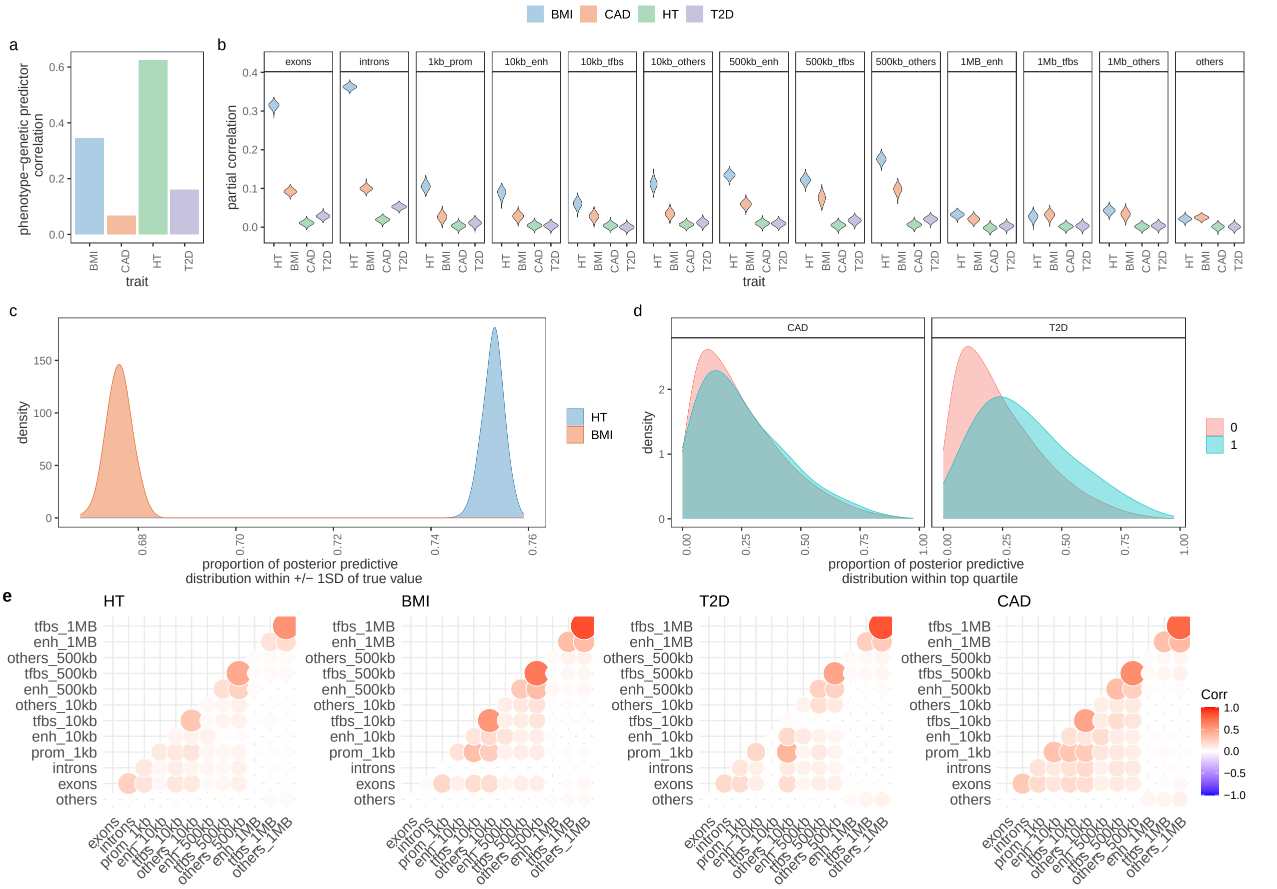

Supplementary Figure 14. Cross-cohort prediction accuracy and the posterior predictive distribution. (a) Correlation of the posterior mean predictor and height (HT), body mass index (BMI), type-2 diabetes (T2D), and cardiovascular disease (CAD). (b) the partial correlations of the phenotype and genomic predictors specific to different genomic annotations. (c) For height and BMI, we calculate the probability that the distribution of genomic predictors obtained for each individual is within 1 SD of the true phenotypic value. The density of these probabilities is shown. (d) For CAD and T2D, we plot density plots of the proportion of the posterior predictive distribution for each individual that is within the top quartile of the risk distribution. (e) Correlation of genetic predictors obtained across annotation groups.

Supplementary Notes

Supplementary Note 1

Model Specification

We begin by outlining the basic model BayesR, before then presenting our extensions. Consider p single nucleotide polymorphism (SNP) markers. If we gather samples for $i = 1, ...N$ subjects in an $N \times p$ matrix, $\mathbf{G}$, in which the elements are coded as 0 for homozygous individuals at the major allele, 1 for heterozygous individuals and 2 for minor allele homozygotes. Now, we wish to model their linear association with the phenotype $\mathbf{y} = (y_i)$ of subjects $i = 1, \ldots, N$ in a standard linear regression model:

$$\mathbf{y} = \mathbf{1}\mu + \mathbf{X}\boldsymbol{\beta} + \boldsymbol{\epsilon} \tag{1}$$

We assume that the genotypes are standardized so that $\mathbf{X}_j = \frac{(\mathbf{G_j} - \mu_j \mathbf{1})}{\sigma_j}$ is the vector of genotypes for the jth marker ($j = 1$, p) with zero mean and unit variance, i.e. the centered and scaled j^{th} column of $\mathbf{G}$. The column's mean $\mu_j \approx 2f_j$ and the column's standard deviation $\sigma_j \approx \sqrt{2f_j\left(1 - f_j\right)}$ being f_j the minor allele frequency (MAF) of the SNP. We define $\boldsymbol{\beta}$ as a $p \times 1$ vector of partial regression coefficients with β_j the effect of a 1 SD change in the jth covariate, and $\boldsymbol{\epsilon}$ is a vector (N x 1) of residuals.

We estimate the model's parameters using Bayesian inference, assuming that the error term $\boldsymbol{\epsilon}|\sigma_\epsilon^2 \sim \mathcal{N}\left(0, \mathbf{I}\sigma_\epsilon^2\right)$. The log-likelihood of this model can be written as

$$l\left(\mu, \boldsymbol{\beta}, \sigma_\epsilon^2\right) = -\frac{N}{2}\log\left(2\pi\sigma_\epsilon^2\right) - \frac{1}{2\sigma_{epsilon}^2}\left(N\left(\hat{y} - \mu\right)^2 + \left(\mathbf{y}_c - \mathbf{X}\boldsymbol{\beta}\right)^T\left(\mathbf{y}_c - \mathbf{X}\boldsymbol{\beta}\right)\right) \tag{2}$$

with $\mathbf{y}_c = \frac{(\mathbf{y} - \mathbf{1}\mu)}{\sigma_y}$ a vector of centred and scaled responses (SD 1).

As we adopt a Bayesian approach, we place priors over the model parameters. For the covariate effects, $\boldsymbol{\beta}$, we use a mixture prior with Dirac spike and slab components, which have been extensively used for variable selection [1, 2]. The prior induces sparsity in the model through a Dirac-delta at zero, excluding variables from the model by setting their coefficients to zero. A slab component is centered at zero and shrinks the non-zero coefficients towards zero according to the slab's width. In our approach, the slab component is a scale mixtures of normals and thus each $\beta_j \in \boldsymbol{\beta}$ is distributed according to:

$$\beta_j \sim \pi_0\delta_0 + \pi_1\mathcal{N}\left(0, \sigma_1^2\right) + \ldots + \pi_L\mathcal{N}\left(0, \sigma_L^2\right)$$

where $\pi_\beta = (\pi_0, \pi_1, \ldots, \pi_L)$ are the mixture proportions, $\left\{\sigma_1^2, \ldots, \sigma_L^2\right\}$ are the mixture-specific variances, and δ_0 is a discrete probability mass at zero. We further constrain the prior by assuming a single parameter representing the total variance explained by the effects σ_G^2, with the component-specific variances proportional to σ_G^2 multiplied by a constant $\{C_1, \ldots, C_L\}$ so that

$$\begin{bmatrix} \sigma_1^2 \\ \vdots \\ \sigma_L^2 \end{bmatrix} = \sigma_G^2 \begin{bmatrix} C_1 \\ \vdots \\ C_L \end{bmatrix}$$

The remaining prior structure for the model is then

$$\begin{aligned} \pi &\sim \text{Dirichlet}\left(\mathbf{1}\right) \\ \sigma_G^2 &\sim \text{Inv} - \text{Scaled}\chi^2\left(\text{v}_0, \text{s}_0^2\right) \\ \sigma_\epsilon^2 &\sim \text{Inv} - \text{Scaled}\chi^2\left(\text{v}_0, \text{s}_0^2\right) \end{aligned} \tag{3}$$

with weakly informative parameters for hyperparameters $\text{v}_0 = \text{s}_0^2 = 0.001$.

For notational convenience, we will refer to the mixture membership labels as $(l_0, l_1, \ldots, l_L)$ and we define a latent indicator of each SNP, j, $\gamma = (\gamma_j, \ldots, \gamma_p)^T$ with $\gamma_{j,l} = 0$ or 1, indicating whether or not the effect of SNP j falls into the zeroth mixture $\gamma_{j,l} = 0$, or follows a normal distribution with variance σ_l^2. We define the "active set of coefficients" as those β_j such that $\beta_j \neq 0$ denoted as $\boldsymbol{\beta}_{\gamma \neq 0}$ with cardinality $||\gamma_\varphi||_0$. Thus the objective of our inference scheme is to compute an estimate of the posterior distribution $f\left(\boldsymbol{\beta}_{\gamma\neq 0}, \sigma_\epsilon^2, \sigma_G^2, \mu|\mathbf{y_c}\right)$. This model has been termed BayesR [3, 4] and an effective proposed Gibbs sampling scheme [4] follows the following steps:

(i) sample μ from $\mathcal{N}\left(\frac{\sum_{i=1}^{N}(\mathbf{y_c}_i-\mathbf{X}_j\boldsymbol{\beta}_{\gamma\neq 0})}{N}, \frac{\sigma_\epsilon^2}{N}\right)$

(ii) sample $\boldsymbol{\beta}_{\gamma\neq 0}$ from its conditional as described below

(iii) sample σ_G^2 from $\text{Inv}-\text{Scaled}\chi^2\left(||\gamma_\varphi||_0+\text{v}_0, \frac{||\gamma_\varphi||_0||\boldsymbol{\beta}_{\gamma\neq 0}||^2+\text{v}_0 S_0^2}{\text{v}_0+||\gamma_\varphi||_0}\right)$

(iv) sample σ_ϵ^2 from $\text{Inv}-\text{Scaled}\chi^2\left(\text{v}_0+N, \frac{||\mathbf{y_c}-\mu-\mathbf{X}\boldsymbol{\beta}_{\gamma\neq 0}||^2+\text{v}_0 S_0^2}{\text{v}_0+N}\right)$

From the former algorithm, steps (i), and (iv) are straight-forward applications of conjugacy and are common to many Gibbs sampling algorithms for linear regression. Step (iii) follows from conjugacy and the assumption that the individual mixtures represent fractions of the total variance explained by the coefficients. Step (ii) is the biggest bottleneck in any linear regression problem, and in the next section we will proceed to detail the derivations of the sampling scheme for this step.

While it is not uncommon to use non-proper priors for the residual's variance σ_ϵ^2, in our case we chose to keep a proper prior for algorithmic and modeling reasons as: (a) conjugacy is amenable to Gibbs sampling (b) we assume σ_ϵ^2 and σ_G^2 are not nuisance parameters, and in some cases we possess prior information on its distribution. It is also common to specify the distribution of β_j having a variance depending on the residual's variance σ_ϵ^2, which would make the estimates transformation-invariant. Recent results suggest the estimates for σ_ϵ^2 in this latter transformation-invariant formulation are biased [5]. Another concern may be that the prior's hyperparameters induce biased estimates for small variances [6], we acknowledge that may be an issue, and allow parameters v_0, s_0^2 to be adjusted if deemed necessary. The scale mixture of Gaussians, allows the prior distribution to have heavier tails than a single Gaussian, which allows big effects to be shrunk to a lesser degree than small effects [7]. Finally, the original formulation of [3,4] assumes $\sigma_G^2 = r^2\sigma_y$ which for centered and scaled phenotypes and genotypes, with heritability h^2 equal to reliability $r^2 = \frac{\text{Var}[\mathbf{X}\boldsymbol{\beta}_{\gamma\neq 0}]}{\text{Var}[y]}$, would mean $\sigma_G^2 = h^2 = r^2 = \text{Var}\left[\mathbf{X}\boldsymbol{\beta}_{\gamma\neq 0}\right] = \sum_{\gamma\neq 0}\boldsymbol{\beta}_{\gamma\neq 0}^2$, but there is no constraint in the model ensuring $\sigma_G^2+\sigma_\epsilon^2=\sigma_y^2$. As we will see, further assumptions are necessary for having unbiased estimates of σ_G^2 and h^2 under varying LD and MAF. These estimates will achieve the equivalence $\sigma_G^2 = r^2 = h^2$ without relying in either using a point estimate of r^2 [3], informative priors on σ_G^2, or normalising the posterior variances by $h^2 = \frac{\sigma_G^2}{\sigma_G^2+\sigma_\epsilon^2}$ [8].

Sampling the effects

For sampling $\boldsymbol{\beta}$, the challenge is two-fold: (a) determining if the effect β_j is part of $\boldsymbol{\beta}_{\gamma\neq 0}$, and if so, to which component it belongs; and then (b) sampling the vector $\boldsymbol{\beta}_{\gamma\neq 0}$ from a multivariate Gaussian with covariance matrix $\Sigma = \mathbf{X}_{l\neq 0}^T\mathbf{X}_{l\neq 0}+\Lambda$ where Λ is the diagonal matrix with entries $\lambda_{l,j} = \frac{\sigma_\epsilon^2}{\sigma_{j,l}^2}$, with $\sigma_{j,l}^2$ the variance of the mixture component to which marker β_j was assigned. For (a), marginalization of each effect individually is required to compute the membership probability, which requires solving a determinant of the size of $||\gamma_\varphi||_0-1$ [2]. For (b), either a system of size $||\gamma_\varphi||_0$ must be solved through LU decomposition, or Cholesky decomposition of size $||\gamma_\varphi||_0$, and both operations are resource intensive when the size of $||\gamma_\varphi||_0$ is large. Instead, we determine the inclusion of a marker in the active set, along with its mixture membership and its partial regression coefficient β_j, in single-site updates. Single-site Gibbs sampling, also known as stochastic relaxation [9], has a long history given its equivalence to iterative Gauss Siedel methods to solve matrix equations [10]. Although we choose to use the BayesR model, many alternative models can easily be placed within the iterative solving and computational framework we outline here.

In this scheme, we sample each element, j, of $\boldsymbol{\beta}$ from the full conditional posterior $f\left(\beta_j|\boldsymbol{\beta}_{\backslash j},\mathbf{y}\right) \propto f\left(\beta_j,\boldsymbol{\beta}_{\backslash j},\mathbf{y}\right)$ which can be written as $f\left(\beta_j,\boldsymbol{\beta}_{\backslash j},\mathbf{y}\right) = f\left(\mathbf{y}|\boldsymbol{\beta}\right)f\left(\beta_j\right)f\left(\boldsymbol{\beta}_{\backslash j}\right)$ where $f\left(\mathbf{y}|\boldsymbol{\beta}\right)$ is the density function of the conditional distribution of $\mathbf{y}|\boldsymbol{\beta}$ and $f\left(\beta_j\right)$ and $f\left(\boldsymbol{\beta}_{\backslash j}\right)$ are the densities of the prior distributions of β_j and $\boldsymbol{\beta}_{\backslash j}$ respectively, with notation $\backslash j$ representing all other covariates except j. The kernel of the full conditional posterior for β_j is proportional to the product of the likelihood, the prior distribution for β_j and the prior distributions of the variances, and thus ignoring factors that are constant with respect to β_j gives

$$f\left(\beta_j \mid l_j,\boldsymbol{\theta}_{\backslash j},\mathbf{y}\right) \propto \exp\left[-\frac{\left(\mathbf{y_c}-\mathbf{X}\boldsymbol{\beta}\right)^T\left(\mathbf{y_c}-\mathbf{X}\boldsymbol{\beta}\right)}{2\sigma_\epsilon^2}\right]\exp\left[-\frac{\beta_j^2}{2\sigma_{j,l}^2}\right] \tag{4}$$

where l_j represents the mixture β_j is assigned, $\boldsymbol{\theta}_{\backslash j} = \{\beta_{\backslash j},\sigma_\epsilon^2,\sigma_G^2,\pi_\beta,\mu\}$ and $\sigma_{j,l}^2$ the corresponding mixture variance. We can reduce the expanded form and drop terms that are free from β_j as

$$\begin{aligned}
f\left(\beta_j \mid l_j, \boldsymbol{\theta}_{\backslash j}, \mathbf{y}\right) &\propto \exp\left[-\frac{1}{2\sigma_\epsilon^2}\left(\mathbf{y_c} - \mathbf{X}_j\beta_j - \mathbf{X}_{\backslash j}\boldsymbol{\beta}_{\backslash j}\right)^T\left(\mathbf{y_c} - \mathbf{X}_j\beta_j - \mathbf{X}_{\backslash j}\boldsymbol{\beta}_{\backslash j}\right) + \frac{\beta_j^2\sigma_\epsilon^2}{2\sigma_{j,l}^2}\right] \\
&\propto \exp\left[-\frac{1}{2\sigma_\epsilon^2}\left(\tilde{\mathbf{y}}^T\tilde{\mathbf{y}} - 2\mathbf{X}_j^T\tilde{\mathbf{y}}\beta_j + \mathbf{X}_j^T\mathbf{X}_j\beta_j^2 + \frac{\beta_j^2\sigma_\epsilon^2}{2\sigma_{j,l}^2}\right)\right] \\
&\propto \exp\left[-\frac{1}{2\sigma_\epsilon^2}\left(\tilde{\mathbf{y}}^T\tilde{\mathbf{y}} - 2\mathbf{X}_j^T\tilde{\mathbf{y}}\beta_j + \beta_j^2\Sigma_{j,l}\right)\right] \\
&\propto \exp\left[-\frac{1}{2\sigma_\epsilon^2}\left(\tilde{\mathbf{y}}^T\tilde{\mathbf{y}} - 2\hat{\beta}_j\Sigma_{j,l}\beta_j + \beta_j^2\Sigma_{j,l} + \hat{\beta}_j^2\Sigma_{j,l} - \hat{\beta}_j^2\Sigma_{j,l}\right)\right] \\
&\propto \exp\left[-\frac{1}{2}\frac{(\beta_j - \hat{\beta}_j)^2}{\frac{\sigma_\epsilon^2}{\Sigma_{j,l}}}\right] \qquad (5)
\end{aligned}$$

with $\tilde{\mathbf{y}} = \mathbf{y}_c - \mathbf{X}_{\backslash j}\boldsymbol{\beta}_{\backslash j}$, $\Sigma_{j,l} = \mathbf{X}_j^T\mathbf{X}_j + \lambda_{j,l}$ and $\hat{\beta}_{j,l} = \frac{\mathbf{X}_j^T\tilde{\mathbf{y}}}{\Sigma_{j,l}}$. This gives the Gibbs sampling update for β_j as

$$\beta_j \sim \mathcal{N}(\Sigma_{j,l}^{-1}\mathbf{X}_j^T\tilde{\mathbf{y}}, \sigma_\epsilon^2\Sigma_{j,l}^{-1}) \qquad (6)$$

To avoid reducibility of the Markov chain, prior to drawing the effect β_j, we first need to select the mixture K for each covariate j, and as above we can condition on the individual coordinates and to obtain the probability that a coefficient j belongs to a given mixture.

$$\mathbb{P}\left(l_j = K \mid \boldsymbol{\theta}_{\backslash j}, \mathbf{y}\right) = \frac{f\left(\tilde{\mathbf{y}} \mid l_j = K, \boldsymbol{\theta}, \mathbf{y}\right)\mathbb{P}\left(l_j = K\right)}{\sum_{k=1}^{L} f\left(\tilde{\mathbf{y}} \mid l_j = k, \boldsymbol{\theta}, \mathbf{y}\right)\mathbb{P}\left(l_j = k\right)} \qquad (7)$$

We integrate out the β_j coordinate following the equations above with

$$\begin{aligned}
f\left(\tilde{\mathbf{y}} \mid l_j, \boldsymbol{\theta}, \mathbf{y}\right) &= \int f\left(\tilde{\mathbf{y}} \mid \beta_j, \sigma_\epsilon^2\right) f\left(\beta_j \mid l_j, \sigma_{j,l}^2\right) d\beta_j \\
&= \int (2\pi\sigma_\epsilon^2)^{-n/2} \exp\left[-\frac{(\tilde{\mathbf{y}} - \mathbf{X}_j\beta_j)^T(\tilde{\mathbf{y}} - \mathbf{X}_j\beta_j)}{2\sigma_\epsilon^2}\right] (2\pi\sigma_{j,l}^2)^{-q/2} \exp\left[-\frac{\beta_j^2}{2\sigma_{j,l}^2}\right] d\beta_j
\end{aligned}$$

where $q = 2$. We then expand this equation using the relationship $\Sigma_{j,l}\hat{\beta}_j = \mathbf{X}_j^T\tilde{\mathbf{y}}$ from Eq. 6 and complete the squares

$$\begin{aligned}
f\left(\tilde{\mathbf{y}} \mid l_j, \boldsymbol{\theta}, \mathbf{y}\right) &= \int (2\pi\sigma_{j,l}^2)^{-q/2}(2\pi\sigma_\epsilon^2)^{-n/2} \exp\left[-\frac{1}{2\sigma_\epsilon^2}\left(\tilde{\mathbf{y}}^T\tilde{\mathbf{y}} - 2\hat{\beta}_{j,l}\Sigma_{j,l}\beta_j + \beta_j^2\Sigma_{j,l} + \hat{\beta}_{j,l}^2\Sigma_{j,l} - \hat{\beta}_{j,l}^2\Sigma_{j,l}\right)\right] d\beta_j \\
&= (2\pi|\sigma_\epsilon^2\Sigma_{j,l}^{-1}|)^{1/2}(2\pi\sigma_{j,l}^2)^{-q/2}(2\pi\sigma_\epsilon^2) \exp\left[-\frac{1}{2\sigma_\epsilon^2}\left(\tilde{\mathbf{y}}^T\tilde{\mathbf{y}} - \hat{\beta}_{j,l}^2\Sigma_{j,l}\right)\right] \times \\
&\int (2\pi|\sigma_\epsilon^2\Sigma_{j,l}^{-1}|)^{-1/2} \exp\left[-\frac{1}{2\sigma_\epsilon^2}\left(\beta_j - \hat{\beta}_{j,l})^2\Sigma_{j,l}\right)\right] d\beta_j \\
&= \left(|\lambda_{l,j}\Sigma_{j,l}^{-1}|\right)^{\frac{1}{2}} \left(2\pi\sigma_\epsilon^2\right)^{-\frac{n}{2}} \exp\left[-\frac{1}{2\sigma_\epsilon^2}\left(\tilde{\mathbf{y}}^T\tilde{\mathbf{y}} - \hat{\beta}_{j,l}^2\Sigma_{j,l}\right)\right] \qquad (8)
\end{aligned}$$

where the final reduction in Eq. 8 occurs as the integral component is now a normal distribution that integrates to 1 and then terms are removed that do not contain, nor depend upon $\Sigma_{j,l}$ nor $\hat{\beta}_{j,l}$. The probability for inclusion in the model in the first mixture, as compared to the spike, then depends upon the ratio

$$\frac{f\left(\tilde{\mathbf{y}} \mid l_j = 0, \boldsymbol{\theta}, \mathbf{y}\right)}{f\left(\tilde{\mathbf{y}} \mid l_j = 1, \boldsymbol{\theta}, \mathbf{y}\right)} = \frac{(2\pi\sigma_\epsilon^2)^{-\frac{n}{2}} \exp\left[-\frac{1}{2\sigma_\epsilon^2}(\tilde{\mathbf{y}}^T\tilde{\mathbf{y}})\right]}{\left(|\lambda_{l,j}\Sigma_{j,2}^{-1}|\right)^{\frac{1}{2}} (2\pi\sigma_\epsilon^2)^{-\frac{n}{2}} \exp\left[-\frac{1}{2\sigma_\epsilon^2}\left(\tilde{\mathbf{y}}^T\tilde{\mathbf{y}} - \hat{\beta}_{j,l}^2\Sigma_{j,2}\right)\right]}$$
$$= \left(|\lambda_{l,j}\Sigma_{j,2}^{-1}|\right)^{-\frac{1}{2}} \exp\left[-\frac{1}{2\sigma_\epsilon^2}(\tilde{\mathbf{y}}^T\tilde{\mathbf{y}}) + \frac{1}{2\sigma_\epsilon^2}(\tilde{\mathbf{y}}^T\tilde{\mathbf{y}}) - \frac{1}{2\sigma_\epsilon^2}(\hat{\beta}_{j,l}^2\Sigma_{j,2})\right]$$
$$= \left(|\lambda_{l,j}\Sigma_{j,2}^{-1}|\right)^{-\frac{1}{2}} \exp\left[-\frac{1}{2\sigma_\epsilon^2}(\hat{\beta}_{j,l}^2\Sigma_{j,2})\right] \tag{9}$$

Analogous to equation 9, any comparison between mixtures has the same form and allows us to omit the $\tilde{\mathbf{y}}^T\tilde{\mathbf{y}}$ term. Thus placing Eq.9 into Eq.7 and re-arranging to a numerically more stable version [3] gives

$$\mathbb{P}\left(l_j = K|\boldsymbol{\theta}_{\setminus j}, \mathbf{y}\right) = \frac{1}{1 + \sum_{k=0}^{L} \exp\left[\log(LK_K) - \log(LK_k)\right]} \tag{10}$$

with $\log(LK_0) = \log(\pi_0)$ and $\log(LK_l) = -\frac{1}{2}\left[-\log\left(|\lambda_{l,j}\Sigma_{j,l}^{-1}|\right) - \left(\frac{\hat{\beta}_{j,l}^2\Sigma_{j,l}}{\sigma_\epsilon^2}\right)\right] + \log(\pi_l)$ for l in $(1...L)$.

Having derived the regression coefficients and their inclusion probabilities, fully specifying the BayesR model, we now proceed to: (1) extend this to a BayesRR-RC model in the Methods section; (2) derive a computational implementation that facilitate the application of the model to biobank sized data in Supplementary Note 2; and (3) derive the properties of the model parameters when applied to highly correlated genomic data (under multicollinearity) and compare these to estimates made by other approaches in the field in Supplementary Note 4.

Supplementary Note 2

A Gibbs sampling scheme for biobank size data

For "$p >> n$" regimes, such as in genomics, where the number of covariates is greater than the number of individuals, hierarchical models controlling assumptions over the sparsity of the model are typically proposed, with examples of sparsity-inducing priors like the "spike and slab" prior [1, 11], the Bayesian LASSO [12] and the Horseshoe [13] prior. There are efficient tools to perform Bayesian regression analysis "out-of-the-box" using MCMC and variational inference [14–16], but these methods are limited to problems with explanatory variables in the low thousands of observations. Recent results show that Gibbs samplers for the Horseshoe prior [17], or for the Bayesian LASSO [18], offer a competitive advantage when combined with approximation schemes for problems of high dimensionality (over 100,000 covariates). These latter methods exchange the inversion of the coefficient matrix, for a matrix multiplication, thus reducing complexity from cubic to almost quadratic on the number of variables. However, despite these good properties, scaling these approaches up to a factor of millions of variables remains prohibitive.

We now describe an effective algorithmic implementation of our BayesRR-RC model that scales to millions of individuals, each genotyped at millions of genetic markers. We outline a Gibbs sampling algorithm that enables all sampling steps to utilize genetic data stored in mixed binary/sparse-index representation, reducing computational complexity of a single Gibbs step from $\mathcal{O}(n)$ to $\mathcal{O}(n_z)$, with n_z the number of non-zero genotypes. We then outline a Bulk Synchronous Parallel Gibbs sampling scheme implemented based on a hybrid MPI + OpenMP model, distributing data across MPI tasks over as many compute nodes as required to hold all the data in memory. Uniquely, this enables large-scale genomic data to be split up into smaller manageable segments, whilst still conducting the analysis in the same way, estimating the marker effects jointly.

Algorithm 1: Serial Algorithm for sampling over the posterior distribution $p\left(\mu, \beta, \epsilon, \sigma_\epsilon, \theta\right)$. $\mathbf{X}_{marker_j}$ represents column of $\mathbf{X}$ corresponding to the column j of the vector *marker*. Given that *marker* is shuffled before sampling the effects, this is equivalent to permuting the order of the effects to be sampled.

Data: Coefficient matrix $\mathbf{X}$, measurement vector $\mathbf{y}$, prior hyperparameters $\mathrm{v}_0, \mathrm{s}_0^2$, iterations I

Result: mean μ, effects vector $\boldsymbol{\beta}$, residual vector ϵ, residual variance σ_ϵ^2 and variance contributed by the marker effects, σ_G^2

1 Initialize $\boldsymbol{\beta}, \mu, \sigma_\epsilon^2, \sigma_G^2, \pi_\phi$;
2 $effects = 1, \ldots, p$;
3 $\epsilon = \mathbf{y} - \mu$;
4 **for** $i \leftarrow 1$ **to** I **do**
5   Sample μ;
6   Shuffle $(effects)$;
7   **for** $j \leftarrow 1$ **to** p **do**
8         $\beta_j^{old} = \beta_j$;
9         $\hat{\beta}_{j,l} = \frac{\mathbf{X}_j^T\left(\tilde{\epsilon}+\mathbf{X}_j\beta_j^{old}\right)}{\Sigma_{j,l}}$;
10   Determine mixture component and sample the new value β_j;
11         $\epsilon^{new} = \epsilon + \left(\beta_j^{old} - \beta_j\right)\mathbf{X}_j$;
12   Sample σ_ϵ^2;
13   Sample σ_G^2;

Algorithm 1 provides a full overview of the sampling scheme of the model as it has been previously implemented. For each marker j, we must compute $\hat{\beta}_{j,l}$ to determine which mixture a marker belongs to, before then sampling $\hat{\beta}_{j,l}$ given the mixture group assigned. This quantity depends on the dot product $\mathbf{X}_j^T\mathbf{y}_c$, with $\mathbf{y}_c$ the centred phenotype. If we keep in memory the vector of residuals $\epsilon = \mathbf{y_c} - \mathbf{X}\boldsymbol{\beta}_{\gamma\neq\mathbf{0}}$, then we can compute efficiently $\mathbf{y_c} - \mathbf{X}_{\backslash\mathbf{j}}\boldsymbol{\beta}_{\gamma\neq\mathbf{0}_{\backslash\mathbf{j}}}$ by the update $\mathbf{y_c} - \mathbf{X}_{\backslash\mathbf{j}}\boldsymbol{\beta}_{\gamma\neq\mathbf{0}_{\backslash\mathbf{j}}} = \tilde{\epsilon} + \mathbf{X_j}\beta_\mathbf{j}$, thus sampling from the joint distribution with a complexity $\mathcal{O}\left(p\right)$. The most expensive operation in Algorithm 1 is computing the numerator in step 9: $\mathbf{X}_j^T\left(\tilde{\epsilon} + \mathbf{X}_j\beta_j^{old}\right)$. As the column vector $\mathbf{X}_j$ contains the centered and scaled genotypes, step 9 involves one sum of two dense vectors and a dot product of two dense vectors. However, if we store in memory the mean, μ_j, and standard deviation σ_j of each column of the genotype matrix, we can express the numerator in step 9 with these quantities and the j-th column of the original genotype matrix $\mathbf{G}$ as (with

$\sigma_j^2 = (\mathbf{G}_j - \mu_j \mathbf{1})^T (\mathbf{G}_j - \mu_j \mathbf{1}) / (n-1)$ by definition):

$$
\begin{aligned}
num = & \frac{(\mathbf{G}_j - \mu_j \mathbf{1})^T}{\sigma_j} \left(\epsilon + \beta_j^{old} \frac{(\mathbf{G}_j - \mu_j \mathbf{1})}{\sigma_j} \right) \\
= & \frac{(\mathbf{G}_j - \mu_j \mathbf{1})^T}{\sigma_j} \epsilon + \beta_j^{old} \frac{(\mathbf{G}_j - \mu_j \mathbf{1})^T}{\sigma_j} \frac{(\mathbf{G}_j - \mu_j \mathbf{1})}{\sigma_j} \\
= & \frac{\mathbf{G}_j^T}{\sigma_j} \epsilon - \frac{\mu_j}{\sigma_j} \sum_{i=1}^{n} \epsilon + \beta_j^{old} (n-1)
\end{aligned}
\tag{11}
$$

and we can do the same for the ϵ update:

$$
\epsilon_{new} = \epsilon + \left(\beta_j^{old} - \beta_j\right) \frac{(\mathbf{G}_j - \mu_j \mathbf{1})}{\sigma_j} = \epsilon + \frac{\left(\beta_j^{old} - \beta_j\right)}{\sigma_j} (\mathbf{G}_j - \mu_j \mathbf{1}) \tag{12}
$$

for which we only have to compute the difference of a sparse vector and a dense vector, and the sum of two dense vectors. Finally, to avoid computing $\sum_{i=1}^{n} \epsilon_{new}$ for each marker, we assign a variable to this quantity and update it after each ϵ update as follows (with $\mu_j = \sum_{i=1}^{n} \mathbf{G}_{i,j}/n$ by definition):

$$
\sum_{i=1}^{n} \epsilon_{new} = \sum_{i=1}^{n} \epsilon + \frac{\left(\beta_j^{old} - \beta_j\right)}{\sigma_j} \left(\sum_{i=1}^{n} \mathbf{G}_{i,j} - n\mu_j \right) = \sum_{i=1}^{n} \epsilon \tag{13}
$$

meaning that the sum of ϵ elements is constant during the algorithm execution (as expected as all involved vectors are zero-mean). Therefore, the only quantity to be computed per run (apart from the ϵ update) is the dot product $\frac{\mathbf{G}_j^T}{\sigma_j}\epsilon$ which can also be reduced, as the elements of $\mathbf{G}_j$ can only be either $\{0, 1, 2\}$ with sequence data or hard-coded genotype. We call $\mathcal{I}_1$ the indicator function such that $\epsilon \mathcal{I}_1 = \begin{cases} \epsilon_j & x_j = 1 \\ 0 & else \end{cases}$ and similarly $\epsilon \mathcal{I}_2 = \begin{cases} \epsilon_j & x_j = 2 \\ 0 & else \end{cases}$ which then gives the dot product as $\frac{\mathbf{G}_j^T}{\sigma_j}\epsilon = \frac{\sum \epsilon \mathcal{I}_1 + 2 \sum \epsilon \mathcal{I}_1}{\sigma_j}$ meaning that multiple $\mathcal{O}(n)$ multiplications are now $\mathcal{O}(n_z)$ sums, and also that instead of storing in memory a sparse matrix of elements plus its indexes, we just need to store three ragged arrays of indexes, one for the "1" elements, a second one for the "2" elements, and a third one for the "M"issing elements. Those arrays contain information for all markers processed by a MPI task and are of unsigned integer type (32 bits). They store indices of the 1, 2 and M elements within the marker (i.e. ranging from 0 to $N-1$). It corresponds to the smallest integer type that allows us to scale to hundreds of thousands or millions individuals. On top of those 3 ragged arrays there are two meta-data arrays for each element type which provide the starts and lengths of the 1, 2 and M elements for each marker in the ragged arrays. They are loaded in memory from reading sparse data files stemming from the conversion of the original Plink .bed file and accessed in parallel by the tasks with MPI I/O.

Even though the sparse representation is optimal in number of operations, performance may vary depending on hardware as a vectorised dot product may be faster than sparse dot product. Spatially, the sparse representation is optimal as long as the columns are sparse. In genotype data, even though the expected number of non-zeros per column is given by the average MAF ($\sim 20\%$ in the UK Biobank data), the distribution is long tailed (Supplementary Figure 15). These columns at the tail of the distribution can dominate the total size of the data structure in memory. Encoding a single column has a constant size of $N \times 2$ bits in plink's .bed file format (referred from now on as binary format), while in sparse representation a column has varying size of $n_z \times 32$ bits. If we encode the columns with less than 6% of non-zeros as sparse and the rest in the original binary format, we can have a total memory occupancy of 60% the size of the original genotype matrix in Plink bed format. In Supplementary Figure 15, we represent on panel (b) the distribution of the proportion non-zeros per column of a genotype matrix for $\sim 4 \times 10^5$ individuals and $\sim 1.5 \times 10^7$ SNPs, solid line representing the mean of the distribution and slashed line the median. In panel (c) we show the total size of the data in memory as a function of the threshold used to split between binary and sparse format, in purple we see how the binary representations dominates the total size up until the mean of the distribution, after which, the size of the sparse data structure starts to dominate and ends up being around four times bigger than the original .bed file size (dotted horizontal line). We found the optimal threshold to be around 0.064 (6.4%, Supplementary Figure 15).

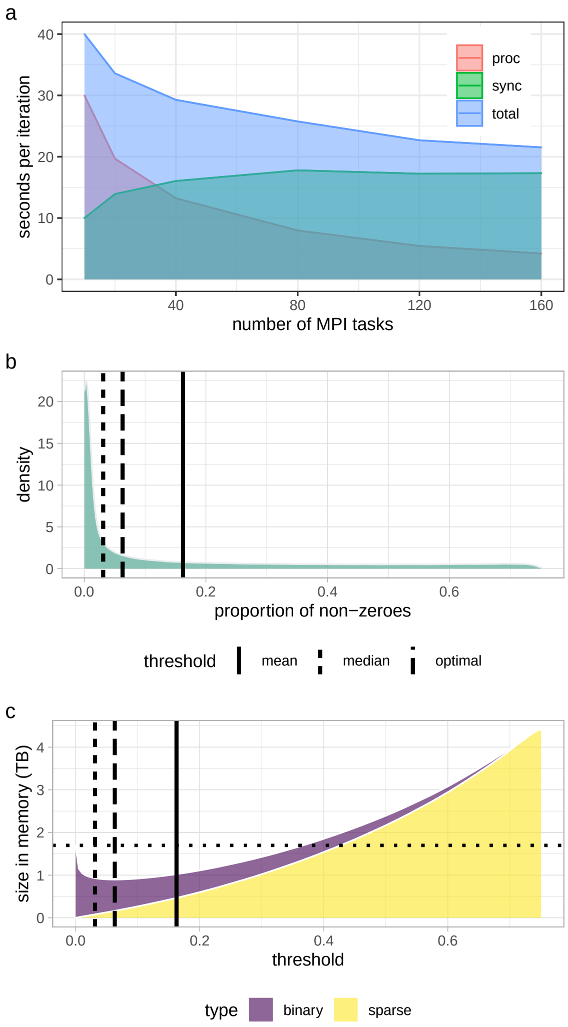

Supplementary Figure 15. A mixed representation bulk synchronous hybrid-parallel Gibbs sampling scheme for genomic data.(a) The minimum seconds per iteration achieved for 382,466 unrelated individuals from the UK Biobank data genotyped at 8,430,466 markers, with an increasing number of message-passing interface (MPI) tasks used. The total seconds is given in blue and this is subset into (i) the time taken to process the markers and estimate all of the 8,433,421 marker effects and hyper-parameters (proc), and (ii) the time taken to synchronise the estimates as they are being obtained (sync). With increasing data parallelism parameter estimation times drop quickly to less than 5 seconds with 160 MPI tasks, however the time taken to synchronise the estimates increases as the number of tasks increases. The SD was 1 second, with variation in sampling times induced by fluctuations in networking speed that influenced the synchronisation times. Each MPI task was able to used 4 CPUs. (b) the distribution of the proportion non-zeros per column of a genotype matrix for $\sim 4 \times 10^5$ individuals and $\sim 1.5 \times 10^7$ SNPs taken from UKB, with solid line representing the mean of the distribution and dashed line the median. (c) the size in memory in TB of the data as the coding of the SNP markers moves from binary to the sparse indexed format, the optimal threshold is achieved between mean and median of the distribution of non-zeros in the genotype matrix. Above this threshold columns are coded in binary format below in sparse index. Through a combination of a mixed data representation and highly vectorized look-up tables, memory usage is reduced while maintaining fast computational speed.

Finally, we implement a vectorized dot product for genotype data stored in the raw binary format based on a couple of look-up tables, by writing the dot product as:

$$\begin{aligned}\frac{(\mathbf{G}_j - \mu_j \mathbf{1})^T}{\sigma_j}\epsilon &= \sum_i \frac{\psi_{i,j}\epsilon_i}{\sigma_j} \\ &= \frac{1}{\sigma_j}\left(\sum_i a_i\epsilon_i - \mu_j \sum_i b_i\epsilon_i\right)\end{aligned} \tag{14}$$

with coefficients a_i and b_i being 0.0, 1.0 or 2.0 depending on the value of $\mathbf{G}_{i,j}$ and following Table 2.

$\mathbf{G}_{i,j}$	0	1	2	NA
2-bit	11	10	00	01
a_i	0.0	1.0	2.0	0.0
b_i	1.0	1.0	1.0	0.0
$\psi_{i,j}$	$0.0 - 1.0\mu_j$	$1.0 - 1.0\mu_j$	$2.0 - 1.0\mu_j$	$0.0 - 0.0\mu_j$

Supplementary Table 2. a and b coefficient values used for building up the two look-up tables needed for the vectorization of the dot product computation when processing binary data.

As 1 byte of plink's .bed can contain $4^4 = 256$ different combinations of information for 4 individuals, we can setup two lookup tables with 256×4 entries each that will give for any byte the corresponding 4 a_i and b_i coefficients, hence allowing for vectorisation of Eq. 14 by performing $a_i\epsilon_i$ and $b_i\epsilon_i$ and accumulating them for 4 individuals at once. Additionally, we use OpenMP to parallelize the loop over the marker's bytes. This greatly extends previously proposed sparse residual updating schemes and also facilitates the synchronous, fully parallel bulk-synchronous Gibbs sampling scheme that we describe in the next section below.

Bulk-synchronous parallel Hogwild Gibbs sampling with sparse data

Bulk-synchronous parallel Hogwild Gibbs sampling [19] assigns block of columns from $\mathbf{X}$ to workers that then sample from $f\left(\beta_j|\boldsymbol{\beta}_{\setminus j}, \mathbf{y}\right)$ for each of the columns in their block. Workers can communicate between each other exchanging the current values of the variables they are sampling, or the whole state of variables for workers in particular. If we perform global synchronisation steps the algorithm is called Bulk-synchronous parallel Hogwild (BSP), if on the other hand, workers exchange messages without a global synchronisation, the algorithm is called Asynchronous parallel Hogwild (ASP) [20].

Algorithm 2: Hogwild Gibbs with '$\Delta\epsilon$-exchange'.

components : Define K parallel workers
Define global variables μ, β, ϵ,π, σ_g^2, σ_e^2;
Initialize variables;
for $i \leftarrow 1$ **to** I **do**
    Update μ;
    Update β in parallel using **DEpsX**(K);
    Update hyperparameters $\pi, \sigma_g^2, \sigma_e^2$;

We propose Algorithm 2, which is a modification of a BSP algorithm where we sample the individual coefficients in parallel conditioned on the hyperparameters. We assign workers (MPI tasks) subsets of coefficients to sample, and each worker performs local Gibbs steps until a global synchronisation is triggered. This global synchronisation happens many times in each iteration, during the phase in which we sample the individual coefficients β_j. For this algorithm, we developed a synchronisation scheme called '$\Delta\epsilon$-exchange'as outlined in Algorithm 3. In this scheme each individual worker is assigned a block of columns from $\mathbf{X}$ and is in charge of sampling from $f\left(\beta_j|\boldsymbol{\beta}_{\setminus j}, \mathbf{y}\right)$ for each of the columns in its block. We add an additional parameter for the synchronisation rate Ω. After Ω columns have been sampled in all workers (around 5-10 in practice to avoid divergence occurring), a synchronisation move is executed.

The purpose of the synchronisation move is to update all of the workers' state based on the coefficients sampled from $t = 1$ until $t = \Omega$ in all workers. The sufficient statistic for this state is contained in the residual vector ϵ. Thus from $t = 1$ until $t = \omega$ each worker computes $f\left(\beta_j|\epsilon_{t=1}\right)$ and keeps track of its local change in ϵ which we denote $\Delta\epsilon = \sum_1^{\Omega} \mathbf{X}_\omega\beta_\omega$ for ω in the set of indexes for the current batch of variables in the workers list of variables. For the synchronisation step, we use the MPI_Allreduce collective, meaning that each task will receive the sum of locally accumulated $\Delta\epsilon$ from all tasks to update its $\epsilon_{t=1} = \sum^{w} \Delta\epsilon_w$ for $w = (1...W)$ workers. With the new $\epsilon_{t=1}$, the worker proceeds to sample the next Ω-sized batch of columns from its set of columns. This synchronisation scheme allows workers to exchange state information in compact form, as the total size of memory occupied in total by the messages is $\mathcal{O}\left(NW\right)$.

Algorithm 3: '$\Delta\epsilon$-exchange'for synchronising changes in backfitted residuals in our BSP Gibbs sampling algorithm.

DEpsX (K)
  components : Set of K workers, each one β_k, Set of K messages, each one $\Delta\epsilon_K$, K sets of $\sim \frac{p}{K}$ columns, each set of columns assigned to a worker.
  foreach *worker* β_k **do**
    $\epsilon_k = \epsilon$;
    $\Delta\epsilon_k = 0$;
    foreach *column* i *in a subset of size* Ω *of the columns assigned to* β_k **do**
      β_j^{old}=β_i;
      draw β_i from $f\left(\beta_i \mid \epsilon, \sigma_\epsilon^2, \sigma_G^2, \pi\right)$;
      $\Delta\epsilon_k$=$\Delta\epsilon_k$-X_i $\left(\beta_i - \beta_j^{old}\right)$;
    Wait until all workers are finished processing their Ω sets;
    $\epsilon = \epsilon + \sum_k \Delta\epsilon_k$;

Previous results point to BSP Gibbs sampling for a multivariate Gaussian converging if the covariance matrix is strictly diagonal-dominant [20] with zero covariance of the markers split across workers. The risk for genomic data, is that two markers in LD get updated at the same time in parallel, double counting their effects, and leading to ϵ being mis-estimated after a synchronization has occurred. Suppose we have one fixed causal marker and two other markers i and j that are assigned to different MPI tasks. Suppose that the Pearson correlation between the causal marker and marker i or j is ρ_i and ρ_j, respectively. Finally, let ρ denote the correlation between the markers i and j. For simplicity in this example suppose that the inclusion probability of the causal marker is q and we make an assumption that the inclusion probability of the marker i is then $P(\beta_i \neq 0) = q\rho_i$ and for marker j it is $P(\beta_j \neq 0) = q\rho_j$, that means that the inclusion probability is proportional to the correlation between causal and other markers. In reality, the effect size estimate is actually proportional to the causal effect: $\hat{\beta}_i = \rho_i\beta_{causal}$ and the function between posterior inclusion probability and causal effect size $q(\beta_{causal})$ is not linear for $\beta_{causal} \geq 0$ as described in Eq.(10) and thus we cannot assume that $P(\beta_i \neq 0) = q\rho_i$ in practice. In the case of parallelising the markers between two tasks we are interested in the probability that two markers from different tasks will absorb the effect of a same causal variant. Thus, we are interested in the probability $P(\beta_i \neq 0, \beta_j \neq 0 | i, j \in U)$, where U is the set of markers that are updated simultaneously in two different tasks. Thus, we can write:

$$P(\beta_i \neq 0, \beta_j \neq 0 | i, j \in U) = P(\beta_i \neq 0)P(\beta_j \neq 0) = q^2\rho_i\rho_j.$$

We see that the probability of making a mistake is dependant on the product $\rho_i\rho_j$. The correlation matrix R of the three markers

$$R = \begin{pmatrix} 1 & \rho_i & \rho_j \\ \rho_i & 1 & \rho \\ \rho_j & \rho & 1 \end{pmatrix}$$

has to be positive semi-definite and thus we can examine what are the possible values for the product $\rho_i\rho_j$ given that we know ρ. Note that the value of ρ can be controlled by providing some blocking mechanism that would assign SNPs to the tasks so that the correlation for the markers from different tasks would be limited to ρ and this is what we advocate here, placing contiguous blocks of markers into different tasks, so as to maximise the LD within a block (MPI task), but minimise the LD across blocks. The maximum possible values for the product follow a linear function that depends on ρ as

$$\max_{\rho_i, \rho_j, \rho = \tilde{\rho}} = 0.5 + 0.5\tilde{\rho}.$$

To get better estimates for the constraints for the product $\rho_i\rho_j$ then we need to make further assumptions about the distribution of ρ_i or ρ_j. Therefore, we can say that $P(\beta_i \neq 0, \beta_j \neq 0 | i, j \in U) \leq q^2(0.5 + 0.5\rho)$. This result and inequality only holds per sampled pair (i, j). We then multiply this result with the probability of sampling the pair (i, j) that both have correlations $\rho_i, \rho_j > 0$. Denoting a set of markers that have a positive correlation with one specific causal marker as the causal radius C, The probability of sampling any pair (i, j) is

$$P(i, j \in U) = \frac{1}{T^2},$$

where T is the number of markers per one task. The probability of pair (i, j) belonging to C is $P(i, j \in C) = c(<< 1)$, some reasonable values could be proposed or estimated for this (for example, $c = (\frac{\#(markers-in-LD)}{2T})^2$). Combining the results together we get that the probability of making a mistake at one update of a pair (i, j):

$$P(\beta_i \neq 0, \beta_j \neq 0) = P(\beta_i \neq 0, \beta_j \neq 0 | (i, j) \in U; (i, j) \in C) P((i, j) \in U) P((i, j) \in C) = P(\beta_i \neq 0, \beta_j \neq 0 | (i, j) \in U) \frac{c}{T^2} \leq q^2 (0.5 + 0.5\rho) \frac{c}{T^2}.$$

This result goes for one fixed causal marker and it also represents the expected number of mistakes per sampled pair (i, j) for one causal marker. If we want to find the expected number of mistakes per sampled pair, we should sum across the P causal markers:

$$Errors \leq \sum_{i=1}^{P} q_i^2 (0.5 + 0.5\rho) \frac{c}{T^2} = (0.5 + 0.5\rho) \frac{c}{T^2} \sum_{i=1}^{P} q_i^2 \leq (0.5 + 0.5\rho) \frac{cP}{T^2}$$

To provide some intuition, we can think of an extreme scenario and assume that there are 100,000 variants in the SNP marker data that would enter the model as they are in LD with underlying causal variants, that each of these variants has posterior inclusion probability of 1, and that for each variant there are two blocks with 30,000 markers in total of which 100 markers have LD = 1 with the causal variant, and that both blocks contain 30,000 markers. Placing these values into what we derive above and sampling over 10,000 iterations leads to probability of an error ~ 0.1 throughout the sampling for this extreme example. Having derived a stable highly parallel Gibbs sampling algorithm for large-scale genomics data, we then performed exhaustive empirical validation of our algorithm in simulation study as described below.

Testing algorithm performance and parallelism in simulation

We explored the influence of increasing parallelism in our algorithm. We used the simulated data described above for the randomly sampled 50,000 UK Biobank individuals with imputed genotype data for chromosome 22, where we sampled randomly 4988 evenly spaced markers as causal variants and randomly assigned the effect sizes from a normal distribution with zero mean and variance 0.6/4988 (the fourth scenario). For each of the 50 simulation replicates, we compared the three chains obtained by running the BayesRR-RC model (with 20 MAF-LD groups) in serial, with a single MPI task and synchronisation rate of 1 (residual updating after sampling each SNP), to three chains obtained by increasing the number of MPI tasks to 4 and then to 8, with synchronisation rates of 10 and 20 sampling steps before residual updating. For each simulation, we ran three chains of our BayesRR-RC model with different starting values for 3000 iterations. Like with all MCMC chains of regression models, convergence and sampling properties will be problem specific and dependent upon the LD of the markers, LD among the causal variants, the phenotypic variation attributable to the SNP markers across the MAF and LD spectrum, the study sample size, the degree of data parallelism per total marker number, and the synchronisation rate. Thus, the aim here is to simply show a series of diagnostic tests that can be utilized to explore the properties of the posterior to highlight how the different metrics can be used to identify convergence issues. We use the distribution, across simulations, of the proportion of effective samples obtained for the hyperparameter estimate of the proportion of phenotypic variance attributable to the markers of each group. This shows that for all ranges of parallelism, we achieve more effective samples for low MAF and low LD variants. As high MAF SNPs are interchangeable in the model to a large degree, their entry and exit from the model is correlated across iterations, and thus this is entirely expected and is actually a consequence of the model mixing. With high synchronisation rates, where many marker updates occur before residual updating by message passing a reduction in effective sample sizes occurs. We also use the distribution of the Gelman-Rubin test statistic for the three chains, a general metric to monitor convergence that compares within- and among-chain variance, as the number of iterations increases. Finally, a Geweke statistic value can be used to test the equality of the means of the first and last part of the Markov chains. We present the results of this simulation in Supplementary Figure 16 also including the distribution of z-scores of the posterior distribution of the phenotypic variance attributable to the markers for each MAF-LD group from the simulated values, which show stability of the estimates obtained with increasing data parallelism (tasks), but that a very high synchronisation rate with high parallelism can lead to poor convergence rates, meaning that the chains would have to be run for longer (Supplementary Figure 16).

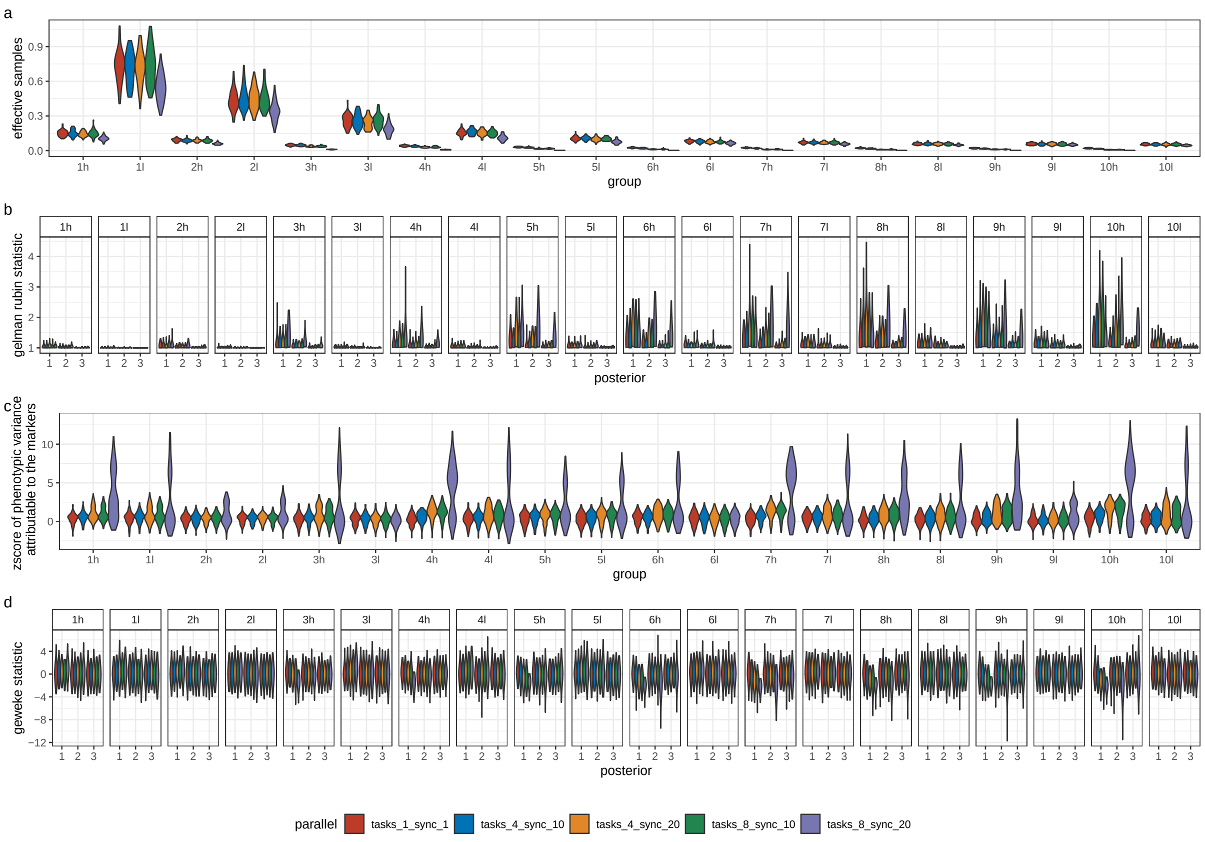

Supplementary Figure 16. Simulation study of increasing task parallelism and increasing message passing rate for our hybrid-parallel sampling scheme. We aimed to compare (a) the effective samples obtained, (b) the convergence rate of the algorithm, (c) the accuracy of the estimation, and (d) the stability of the estimates obtained as data parallelism increases within a burn-in period of the initial 3000 iterations. For 50,000 randomly selected UK Biobank individuals, and 111,425 imputed SNP markers of chromosome 22, we simulated 50 replicate phenotypes by randomly selecting 4,988 SNPs as causal variants and randomly allocating effect sizes from a normal distribution, with SNP heritability of 0.5. For each simulation, we ran three chains of our BayesRR model with different starting values for 3000 iterations. The SNP marker data was grouped into deciles of the distribution of minor allele frequency (MAF) and within each decile the markers were further grouped these into two groups based on the distribution of linkage disequilibrium (LD), giving twenty groups in total (1l = MAF decile 1, low LD; 1h = MAF decile 1, high LD; ...; 10l = MAF decile 10, low LD; 10h = MAF decile 10, high LD). We repeated the three chains, but with increasing data parallelism: (1) in serial where one MPI task is used and the residual is updated after each marker is sampled (tasks__1__sync__1); (2) where the markers were split across four MPI processes with synchronisation occurring by message passing after 10 markers have been updated (task__4__sync__10); (3) where the markers were split across four MPI processes with synchronisation occurring after 20 markers have been updated (task__4__sync__20); (4) with 8 MPI processes and synchronisation of 10 (task__8__sync__10); and (5) with 8 MPI processes and synchronisation of 20 (task__8__sync__20). (a) shows the distribution across simulations of the proportion of effective samples obtained for the hyperparamter estimate of the proportion of phenotypic variance attributable to the markers of each group. For all ranges of parallelism, we achieve more effective samples for low MAF and low LD variants. With high synchronisation rates, where many marker updates occur before residual updating by message passing a reduction in effective sample sizes occurs. (b) gives the distribution of the Gelman-Rubin test statistic for the three chains, a general metric to monitor convergence that compares within- and among-chain variance, as the number of iterations increases. On the x-axis, 1 gives the distribution of the statistic across chains and MAF-LD groups for the first 500 iterations showing divergence of the chains (y-axis value >> 1) across all MAF-LD groups, 2 gives the distribution for the first 1000 iterations, and 3 gives the distribution for the whole chain showing convergence of the chains by the end of this initial 3000 iteration sampling period irrespective of the data parallelism, with the exception of a few groups with infrequent synchronisation and high data parallelism which have yet to converge within this burn-in phase. (c) gives the distribution of z-scores of the posterior distribution of the phenotypic variance attributable to the markers for each MAF-LD group from the simulated values, showing stability of the estimates with increasing data parallelism (tasks), but not with infrequent synchronisation within the 3000 iterations run here. (d) shows the distribution of the Geweke statistic value which is a test of the equality of the means of the first and last part of the Markov chains. On the x-axis, 1 gives the distribution of the statistic calculated using all iterations across all MAF-LD groups, 2 gives the distribution discarding the first 500 iterations, and 3 gives the distribution discarding the first 1000 iterations. (a) - (d) suggest that our hybrid-parallelism sampling scheme achieves the same accuracy and convergence rates as a serial sampling scheme, provided that frequent synchronisation occurs and data parallelism is kept moderate. At high data parallelism and infrequent synchronisation, our theory shows that we are more likely to make a sampling mistake, preventing chains from converging and requiring longer sampling times. Convergence and accuracy of the MCMC Gibbs sampling chain will be problem specific and dependent upon the LD of the markers, LD among the causal variants, the phenotypic variation attributable to the SNP markers across the MAF and LD spectrum, the study sample size, the degree of data parallelism per total marker number, and the synchronisation rate. Therefore, like with all MCMC chains, a series of diagnostic tests can be utilized to explore the properties of the posterior and here we show how different metrics can be used to identify convergence issues.

Implementation and processing setup

We implement algorithms 2 and 3 in C++ as a pure CPU MPI + OpenMP hybrid solution. All data structures were properly aligned in memory to assist vectorization and assembly code was examined to ensure that the code was properly vectorized where expected. We utilize the scientific library boost (see Code Availability) and we profiled and benchmarked the code with Intel performance analysis tools such as Advisor and Ampflier. Current implementation requires to be compiled with Intel compiler on an architecture supporting at least AVX2 although support for AVX512 is recommended for performance. UK Biobank results were generated on the cluster Helvetios from EPFL (see Code Availability) using 10 compute nodes and setting 8 MPI tasks per node and dedicating 4 (physical) cores to each task. 10 is the minimal number of nodes that was required to hold all the data in memory in its mixed-representation. An overview of the run times and memory use are provided in Supplementary Figure 15.

Supplementary Note 3

Posterior summaries and discovery

The ability of the additive regression model outlined and applied here to infer the underlying distribution of genomic effects is limited unless an additive model with many 0 coefficients holds as approximately true and the true number of underlying nonzero coefficients is $<< n$. Various ad hoc penalty functions in machine learning, and the range of proper priors employed by members of the Bayesian alphabet and beyond, all impose a restriction on the size of the regression coefficients, and while these restrictions differ, they all provide shrinkage estimators that by their definition are biased as they are shrunk toward zero (this is true of mixed-linear association models also). In other words, the penalty function (prior) will be important and will influence the inference made here. Thus, the inference we obtain can only be made with respect to our *a priori* assumption that many marker effects are zero, and that the effects of those that are not zero can be reflected by a mixture of zero centred Gaussian distributions. Given this, we focused on comparing the posterior distributions of different traits obtained under the same model, focusing on the hyper-parameter estimates obtained for MAF-LD-annotation groups, and comparing these across traits. It has been shown in Bayesian penalized regression models that what is learned about β is a function of what is learned about $\mathbf{X}\boldsymbol{\beta}$ and thus by placing separate hyper-parameters over different genomic groups we can obtain inference as to the variance contributed by each group [21]. As we show through theory and simulation study described below, MAF-LD-annotation specific hyper-parameters likely results in improved inference as to the distribution of genetic effects. However, with the exception of very rare variants with LD ~ 0, we cannot treat each β_j as independent and thus here we outline a strategy to identify associated genes, or genomic regions within a probabilistic framework.

For a simple example, consider two markers in LD that are correlated with a single causal variant, where either or both markers may be in the model at any one iteration and the expected posterior inclusion probability of each SNP is 0.5. In this scenario, we cannot use the posterior inclusion probability of each marker to assess association and thus instead, we take an approach of assessing the contribution of different genomic regions to trait variation whilst controlling the posterior type I error rate (PER), which is more suitable controlling for false positives, than controlling the genome-wide error rate (GER). Many papers have discussed the advantages of controlling the false discovery rate (FDR), and related measures rather than controlling GER [22] and here we follow [23] where the posterior probability that β_j is nonzero for at least one SNP j in a window or genomic segment is used to make inferences on the presence of an association in that segment.

Briefly, following [23], we will refer to this probability as the window posterior probability of association (WPPA). The underlying assumption is that if a genomic window contains a marker in LD with a causal variant, one or more SNPs in that window will have nonzero β_j. Thus, WPPA, which is estimated by counting the number of MCMC samples in which β_j is nonzero for at least one SNP j in the window, can be used as a proxy for the posterior probability that the genomic region contains a causal variant. Because WPPA for a given window is a partial association conditional on all other SNPs in the model, including those flanking the region, the influence of flanking markers on the WPPA signal for any given window will be inversely related to the distance k of the flanking markers. Thus, as the number of markers between a causal variant and the focal window increases, the influence of the causal variant on the WPPA signal will decrease and so WPPA computed for a given window can be used to locate associations for that given window [23].

This measure can be shown to control the PER, which in frequentest statistics would be associated with the test of a hypothesis. The null hypothesis in this case is that the genomic region does not contain any SNPs associated with the trait. Using this notation, WPPA is the conditional probability that the null is false given the observed data, while PER is the conditional probability that the null hypothesis is true given that it has been rejected based on some statistical test. Suppose the test is based on WPPA and the null is rejected whenever WPPA is larger than some value t. Then, PER is the probability that the null hypothesis is true given WPPA is larger than t, and it can be written as:

$$\text{PER} = \Pr(H_0 \text{is true}|\text{WPPA} > t) = E[(1 - \text{WPPA})|\text{WPPA} > t] \tag{15}$$

Thus, for any interval with WPPA $> t$ the proportion of false positives among significant results will be $\leq (1 - t)$. Here, we are interested in detecting genes and genomic regions that explain more than some proportion v of the total phenotypic variance attributable to the SNP markers (genetic variance). The genomic segment variance is defined as the sum of the squared partial regression coefficient estimates at each iteration and these are divided by the sum of all the squared partial regression coefficient estimates genome-wide to give a proportion for each genomic region at each iteration. Then we simply count the

proportion of MCMC samples where the proportion of genetic variance is greater than a thresholds of 0.001% and we denote this metric as the posterior probability of window variance (PPWV).

We extend this PPWV approach to develop an association metric for LD blocks of the genome. Currently, association studies predominantly estimate SNP effects and test for association one marker at a time, which does not control for local LD among SNP markers. Thus, the level of association determined is at the regional level as results are reduced, using LD patterns, to a subset of the strongest associated LD-independent variables. We can solve the problem of having a correlated posterior distribution by applying our PPWV approach to the LD blocks of the genome providing a Bayesian probabilistic metric of association that is equivalent to selecting the number of independent associated SNP markers. We define LD blocks as a group of SNPs that have squared correlation greater that 0.15 and then for each iteration, we sum the squared partial regression coefficient estimates for all the SNPs within the block, divide this by the sum of all the squared partial regression coefficient estimates genome-wide to give a proportion for each genomic region at each iteration. Then we simply count the proportion of MCMC samples where the proportion of genetic variance is greater than a thresholds of 0.001% providing a probabilistic association metric for each LD block that controls the FDR genome-wide. Within each associated region the individual SNP posterior inclusion probabilities can then also be used to "fine-map" the associations, in order to select the base-pair position that is most likely to be closest to the true underlying causal variant in imputed SNP data.

Supplementary Note 4

Comparison to other approaches under collinearity

Genome-wide association studies have predominantly been conducted using single marker regression via ordinary least squares (OLS). Recently, it has been proposed that if aggregation due to familial or molecular similarity (e.g. population stratification) exists in the data, a better estimation approach is generalized least squares (GLS), as it poses a more general covariance structure than OLS. GLS estimates can be obtained within mixed-linear association models, which first declare all marker effects as random variables, for example, assuming that $u_j \sim N(0, \sigma_u^2)$, or from a mixture of distributions, with all markers in the set taken as independently and identically distributed random variables. Second, when the markers are evaluated for association, they are then treated as a fixed effect. The resulting model can be written as

$$\mathbf{y} = \mathbf{X}_1\beta_1 + \mathbf{X}_1 u_1 + \mathbf{X}_{\backslash 1}\mathbf{u}_{\backslash 1} + \boldsymbol{\epsilon} \tag{16}$$

where a focal genetic marker, here $\mathbf{X}_1$ is fitted twice, first as a fixed effect to estimate the regression coefficient β_1, and also as part of all of the other markers with their effects, u, estimated as random (note here \1 indicates all markers other than marker 1). Under this model the phenotypic covariance structure is

$$\mathbf{V} = \mathbf{X}_1\mathbf{X}_1^T\sigma_G^2 + \mathbf{X}_{\backslash 1}\mathbf{X}_{\backslash 1}^T\sigma_G^2 + \mathbf{I}\sigma_\epsilon^2 \tag{17}$$

With orthogonal covariates, the estimated variance components that compose $\mathbf{V}$ can remain constant when testing each marker in turn. However, with collinearity among markers the situation becomes more complex. Below, we first describe the impact of multicollinearity on ridge regression estimates. We then outline the equivalence of a ridge regression and a mixed linear model, before then demonstrating increased variance of the estimates obtained from Eq. (16) under multicollinearity. Finally, we then go on to show that estimates from BayesR are less subject to inflated variance, except under extensive multicollinearity, before then describing how extending the model to provide minor allele frequency and LD specific hyperparameters provides estimates with improved properties across a range of underlying generative data models.

In Eq. (16) if markers were all simply estimated as random, following a single distribution, then a ridge regression estimator of Hoerl and Kennard 1970 [24] would be obtained, which was proposed to replace $\mathbf{X}^T\mathbf{X}$ in the OLS solutions by $\mathbf{X}^T\mathbf{X} + \lambda\mathbf{I}$, with $\lambda \in [0, \infty]$ a tuning or penalty parameter. This gives the ridge regression estimator

$$\hat{\boldsymbol{\beta}}(\lambda) = [\mathbf{X}^T\mathbf{X} + \lambda\mathbf{I}]^{-1}\mathbf{X}^T\mathbf{Y} \tag{18}$$

where λ is strictly positive and the solution or regularization path of the ridge estimate $\hat{\boldsymbol{\beta}}(\lambda) : \lambda \in [0, \infty]$ is the set of ridge estimates across the values of λ. The expectation of the ridge estimator

$$\begin{aligned}\mathbb{E}[\hat{\boldsymbol{\beta}}(\lambda)] &= \mathbb{E}[(\mathbf{X}^T\mathbf{X} + \lambda\mathbf{I})^{-1}\mathbf{X}^T\mathbf{Y}] \\ &= (\mathbf{X}^T\mathbf{X} + \lambda\mathbf{I})^{-1}\mathbf{X}^T\mathbb{E}(\mathbf{Y}) \\ &= (\mathbf{X}^T\mathbf{X} + \lambda\mathbf{I})^{-1}(\mathbf{X}^T\mathbf{X})\boldsymbol{\beta}\end{aligned} \tag{19}$$

with $\hat{\boldsymbol{\beta}}$ the maximum likelihood OLS estimator. If we consider an orthonormal design matrix $\mathbf{X}$, with $\mathbf{X}^T\mathbf{X} = \mathbf{I} = (\mathbf{X}^T\mathbf{X})^{-1}$ then we can express the relationship between $\hat{\boldsymbol{\beta}}$, and the ridge estimator, $\hat{\boldsymbol{\beta}}(\lambda)$, as

$$\begin{aligned}\hat{\boldsymbol{\beta}}(\lambda) &= (\mathbf{X}^T\mathbf{X} + \lambda\mathbf{I})^{-1}\mathbf{X}^T\mathbf{Y} \\ &= (\mathbf{I} + \lambda\mathbf{I})^{-1}\mathbf{X}^T\mathbf{Y} \\ &= (1 + \lambda\mathbf{I})^{-1}\mathbf{I}\mathbf{X}^T\mathbf{Y} \\ &= (1 + \lambda\mathbf{I})^{-1}(\mathbf{X}^T\mathbf{X})^{-1}\mathbf{X}^T\mathbf{Y} \\ &= (1 + \lambda\mathbf{I})^{-1}\hat{\boldsymbol{\beta}}\end{aligned} \tag{20}$$

If we define $\mathbf{W}_\lambda = (\mathbf{X}^T\mathbf{X} + \lambda\mathbf{I})^{-1}(\mathbf{X}^T\mathbf{X})$ then the ridge estimator $\hat{\boldsymbol{\beta}}(\lambda)$ can be expressed as $\mathbf{W}_\lambda\hat{\beta}$ for

$$\begin{aligned}\mathbf{W}_\lambda\hat{\boldsymbol{\beta}} &= \mathbf{W}_\lambda(\mathbf{X}^T\mathbf{X})^{-1}\mathbf{X}^T\mathbf{Y} \\ &= [(\mathbf{X}^T\mathbf{X})^{-1}(\mathbf{X}^T\mathbf{X} + \lambda\mathbf{I})]^{-1}(\mathbf{X}^T\mathbf{X})^{-1}\mathbf{X}^T\mathbf{Y} \\ &= (\mathbf{X}^T\mathbf{X} + \lambda\mathbf{I})^{-1}\mathbf{X}^T\mathbf{Y} \\ &= \hat{\boldsymbol{\beta}}(\lambda)\end{aligned} \tag{21}$$

The variance of the ridge estimator is then

$$\begin{aligned}\mathrm{Var}[\hat{\boldsymbol{\beta}}(\lambda)] &= \mathrm{Var}[\mathbf{W}_\lambda \hat{\boldsymbol{\beta}}] \\ &= \mathbf{W}_\lambda \mathrm{Var}[\hat{\boldsymbol{\beta}}]\mathbf{W}_\lambda^T \\ &= \sigma_\epsilon^2 \mathbf{W}_\lambda (\mathbf{X}^T\mathbf{X})^{-1}\mathbf{W}_\lambda^T \\ &= \sigma_\epsilon^2 (\mathbf{X}^T\mathbf{X} + \lambda\mathbf{I})^{-1}\mathbf{X}^T\mathbf{X}[(\mathbf{X}^T\mathbf{X}+\lambda\mathbf{I})^{-1}]^T\end{aligned} \tag{22}$$

and the mean square error of $\hat{\boldsymbol{\beta}}(\lambda)$ is

$$\begin{aligned}\mathbf{MSE}[\hat{\boldsymbol{\beta}}(\lambda)] &= \mathbb{E}[(\mathbf{W}_\lambda\hat{\boldsymbol{\beta}})^T(\mathbf{W}_\lambda\hat{\boldsymbol{\beta}})] \\ &= \mathbb{E}(\hat{\boldsymbol{\beta}}^T\mathbf{W}_\lambda^T\mathbf{W}_\lambda\hat{\boldsymbol{\beta}}) - \mathbb{E}(\boldsymbol{\beta}^T\mathbf{W}_\lambda\hat{\boldsymbol{\beta}}) - \mathbb{E}(\hat{\boldsymbol{\beta}}^T\mathbf{W}_\lambda^T\boldsymbol{\beta}) + \mathbb{E}(\boldsymbol{\beta}^T\boldsymbol{\beta}) \\ &= \mathbb{E}(\hat{\boldsymbol{\beta}}^T\mathbf{W}_\lambda^T\mathbf{W}_\lambda\hat{\boldsymbol{\beta}}) - \mathbb{E}(\boldsymbol{\beta}^T\mathbf{W}_\lambda^T\mathbf{W}_\lambda\hat{\boldsymbol{\beta}}) - \mathbb{E}(\hat{\boldsymbol{\beta}}^T\mathbf{W}_\lambda^T\mathbf{W}_\lambda\boldsymbol{\beta}) + \mathbb{E}(\boldsymbol{\beta}^T\mathbf{W}_\lambda^T\mathbf{W}_\lambda\boldsymbol{\beta}) \\ &\quad - \mathbb{E}(\boldsymbol{\beta}^T\mathbf{W}_\lambda^T\mathbf{W}_\lambda\boldsymbol{\beta}) + \mathbb{E}(\boldsymbol{\beta}^T\mathbf{W}_\lambda^T\mathbf{W}_\lambda\hat{\boldsymbol{\beta}}) + \mathbb{E}(\hat{\boldsymbol{\beta}}^T\mathbf{W}_\lambda^T\mathbf{W}_\lambda\boldsymbol{\beta}) \\ &\quad - \mathbb{E}(\boldsymbol{\beta}^T\mathbf{W}_\lambda\boldsymbol{\beta}) - \mathbb{E}(\hat{\boldsymbol{\beta}}^T\mathbf{W}_\lambda^T\boldsymbol{\beta}) - \mathbb{E}(\boldsymbol{\beta}^T\boldsymbol{\beta}) \\ &= \mathbb{E}[(\hat{\boldsymbol{\beta}}-\boldsymbol{\beta})^T\mathbf{W}_\lambda^T\mathbf{W}_\lambda(\hat{\boldsymbol{\beta}}-\boldsymbol{\beta})] \\ &\quad - \boldsymbol{\beta}^T\mathbf{W}_\lambda^T\mathbf{W}_\lambda\boldsymbol{\beta} + \boldsymbol{\beta}^T\mathbf{W}_\lambda^T\mathbf{W}_\lambda\boldsymbol{\beta} + \boldsymbol{\beta}^T\mathbf{W}_\lambda^T\mathbf{W}_\lambda\boldsymbol{\beta} - \boldsymbol{\beta}^T\mathbf{W}_\lambda\boldsymbol{\beta} - \boldsymbol{\beta}^T\mathbf{W}_\lambda\boldsymbol{\beta} + \boldsymbol{\beta}^T\boldsymbol{\beta} \\ &= \mathbb{E}[(\hat{\boldsymbol{\beta}}-\boldsymbol{\beta})^T\mathbf{W}_\lambda^T\mathbf{W}_\lambda(\hat{\boldsymbol{\beta}}-\boldsymbol{\beta})] + \boldsymbol{\beta}^T(\mathbf{W}_\lambda-\mathbf{I})^T(\mathbf{W}_\lambda-\mathbf{I})\boldsymbol{\beta} \\ &= \sigma_\epsilon^2\mathrm{tr}[\mathbf{W}_\lambda(\mathbf{X}^T\mathbf{X})^{-1}\mathbf{W}_\lambda^T] + \boldsymbol{\beta}^T(\mathbf{W}_\lambda-\mathbf{I})^T(\mathbf{W}_\lambda-\mathbf{I})\boldsymbol{\beta}\end{aligned} \tag{23}$$

The first summand is the sum of the variances of the ridge estimator, while the second summand is the squared bias of the ridge estimator. With an orthonormal design matrix, $\mathbf{X}$, Theorem 2 of Theobald 1974 [25] shows:

$$\mathbf{MSE}[\hat{\boldsymbol{\beta}}(\lambda)] = \frac{p\sigma_\epsilon^2}{(1+\lambda)^2} + \frac{\lambda^2}{(1+\lambda)^2}\boldsymbol{\beta}^T\boldsymbol{\beta} \tag{24}$$

which achieves a minimum at $\lambda = p\sigma_\epsilon^2/\boldsymbol{\beta}^T\boldsymbol{\beta} = \sigma_\epsilon^2/\sigma_\beta^2$, with σ_β^2 the variance of the β coefficients. This has been stated in the genetics literature as the optimal shrinkage parameter [26] for a ridge regression. However, this is derived under the assumption of uncorrelated covariates within the design matrix $\mathbf{X}$.

To explore the effects of correlated covariates we use the ridge loss function, defined as

$$\mathcal{L}_{ridge}(\boldsymbol{\beta};\lambda) = ||\mathbf{Y}-\mathbf{X}\boldsymbol{\beta}||_2^2 + \lambda||\boldsymbol{\beta}||_2^2 = \sum_{i=1}^n (Y_i - \mathbf{X}_i\boldsymbol{\beta})^2 + \lambda\sum_{j=1}^p \beta_j^2 \tag{25}$$

which is the sums-of-squares with a penalty, $\lambda\sum_{j=1}^p \beta_j^2$, referred to as the ridge penalty, which shrinks the regression coefficients towards zero. The radius of the ridge constraint, the squared Euclidean norm of β, $||\beta||_2^2$, depends upon λ, $\mathbf{X}$ and $\mathbf{Y}$, and taking its expectation

$$\begin{aligned}\mathbb{E}[||\hat{\beta}(\lambda)||_2^2] &= \mathbb{E}\left[[(\mathbf{X}^T\mathbf{X}+\lambda\mathbf{I})^{-1}(\mathbf{X}^T\mathbf{X})\hat{\boldsymbol{\beta}}]^T(\mathbf{X}^T\mathbf{X}+\lambda\mathbf{I})^{-1}(\mathbf{X}^T\mathbf{X})\hat{\boldsymbol{\beta}}\right] \\ &= \mathbb{E}[\mathbf{Y}^T\mathbf{X}(\mathbf{X}^T\mathbf{X}+\lambda\mathbf{I})^{-2}\mathbf{X}^T\mathbf{Y}] \\ &= \sigma_\epsilon^2\mathrm{tr}[\mathbf{X}(\mathbf{X}^T\mathbf{X}+\lambda\mathbf{I})^{-2}\mathbf{X}^T] + \boldsymbol{\beta}\mathbf{X}^T\mathbf{X}(\mathbf{X}^T\mathbf{X}+\lambda\mathbf{I})^{-2}\mathbf{X}^T\mathbf{X}\boldsymbol{\beta}\end{aligned} \tag{26}$$

provides a measure that can be evaluated given different properties of the design matrix $\mathbf{X}$. With the same λ and the same $\boldsymbol{\beta}$, Eq. (26) shows that the degree of collinearity among the covariates alters the variance of the estimated effects. Thus, in a ridge regression penalization does not remove collinearity but simply reduces its effects on the variance of the ridge estimator provided that the λ value is sufficiently large (and thus the σ_β^2 is small). We explore Eq. (26) in a simulation study described below and presented in Figure S1, Figure 1 and Figure 2. This theory is an extension of previous work [27] which showed that the inflation of the SNP heritability is proportional to a ratio of the average LD among causal variants and the markers and the average LD among all the markers, with inflation expected when causal variants are in higher LD with the markers than on average. Eq. (26) is a function of X'X, with the LD values the off-diagonal elements in X'X, but it suggests that inflation would be irrespective of the average LD across the genome, simply being expected if high-LD markers had strong effects and showing that inflation would occur only for the estimates of markers that are in LD with those causal variants. Thus, if SNP heritability is allocated across SNPs at random then estimation will on average be correct, irrespective of the LD among SNPs. If the effects of SNPs

vary according to the MAF or LD of the SNP, and assumptions are made that all SNP effects are sampled from the same distribution, then this will lead to bias as the estimates at high-LD markers in strong LD with underlying causal variants will be inflated and this inflation will be sufficiently large and occur at a sufficient number of genomic locations so as to impact upon the global estimate of SNP heritability.

This issue has been detected, and demonstrated in simulation, in a number of recent papers [28–31]. However, to date it has remained little understood from a theoretical perspective. The LD-MAF corrections proposed in the literature all serve to alter the lambda value for SNPs, or sets of SNPs, so that it becomes proportional to the LD and MAF of the marker, in essence reducing the σ_G^2, or making it more specific to the markers in question, and increasing the λ value for common, highly correlated covariates. The equivalence of ridge regression and mixed-linear models has been shown many times, using well-established results from prediction of random variables dating back to Henderson [32]. The model $\mathbf{Y} = \mathbf{g} + \boldsymbol{\epsilon}$, with $\mathbf{g}$ the genetic value of the individuals, and the model $\mathbf{Y} = \mathbf{X}\boldsymbol{\beta} + \boldsymbol{\epsilon}$, with $\mathbf{g} = \mathbf{X}\boldsymbol{\beta}$, $\mathbf{g} \sim N(0, \mathbf{X}\mathbf{X}^T\sigma_G^2)$ with marker effects thus $\boldsymbol{\beta} = \mathbf{X}^T(\mathbf{X}\mathbf{X}^T)^{-1}\mathbf{g}$, are equivalent. Following Henderson [32], assuming σ_ϵ^2 and σ_G^2 are known, with no fixed effect component, the log-likelihood can be shown to be proportional to:

$$\sigma_\epsilon^{-2}\|\mathbf{Y} - \mathbf{g}\|_2^2 + \mathbf{g}^T\mathbf{I}\sigma_G^2\mathbf{g} \tag{27}$$

equating the partial derivatives of this mixed model loss function with respect to $\mathbf{g}$ to zero, yields the estimating equations known as Henderson's mixed model equations. Returning to the mixed linear association model described in Eq.(16), using $\mathbf{u}$ to denote the marker effects estimated as random, β for the focal marker effect estimated as fixed, and assuming independent marker effects, Henderson's mixed model equations (MME) take the form:

$$\begin{bmatrix} \mathbf{X}_1^T\mathbf{X}_1 & \mathbf{X}_1^T\mathbf{X}_1 & \mathbf{X}_1^T\mathbf{X}_{\setminus 1} \\ \mathbf{X}_1^T\mathbf{X}_1 & \mathbf{X}_1^T\mathbf{X}_1 + \mathbf{I}\lambda & \mathbf{X}_1^T\mathbf{X}_{\setminus 1} \\ \mathbf{X}_{\setminus 1}^T\mathbf{X}_1 & \mathbf{X}_{\setminus 1}^T\mathbf{X}_1 & \mathbf{X}_{\setminus 1}^T\mathbf{X}_{\setminus 1} + \mathbf{I}\lambda \end{bmatrix} \begin{bmatrix} \beta_1 \\ u_1 \\ \mathbf{u}_{\setminus 1} \end{bmatrix} = \begin{bmatrix} \mathbf{X}_1^T\mathbf{y} \\ \mathbf{X}_1^T\mathbf{y} \\ \mathbf{X}_{\setminus 1}^T\mathbf{y} \end{bmatrix} \tag{28}$$

where $\lambda = \frac{\sigma_\epsilon^2}{\sigma_\beta^2}$. Subtracting the u_1 from the β equations gives $u_1 = 0$ and thus the MME reduce to:

$$\begin{bmatrix} \mathbf{X}_1^T\mathbf{X}_1 & \mathbf{X}_1^T\mathbf{X}_{\setminus 1} \\ \mathbf{X}_1^T\mathbf{X}_{\setminus 1} & \mathbf{X}_{\setminus 1}^T\mathbf{X}_{\setminus 1} + \mathbf{I}\lambda \end{bmatrix} \begin{bmatrix} \beta_1 \\ \mathbf{u}_{\setminus 1} \end{bmatrix} = \begin{bmatrix} \mathbf{X}_1^T\mathbf{y} \\ \mathbf{X}_{\setminus 1}^T\mathbf{y} \end{bmatrix} \tag{29}$$

This has been derived previously [33], however there is an explicit assumption that any estimation error of the random marker effect estimates go into the residual and does not influence the fixed estimate of the marker. For the random effect component, the equivalence with the ridge regression estimator of Eq.(18) is evident, as is the equivalence of Eq. (27) with Eq. (25) above. Thus an MLMAi model returns "ridge regression" estimate of the marker effects, and as we show above ridge regression estimates are inflated when effect sizes are higher for high LD markers. It then follows that mixed model effect size estimates could be biased when effect sizes are higher for high LD markers.

Seen in this light, we can now explore the influence of multicollinearity on the BayesR dirac spike and slab model described above and compare it to that of a ridge regression. If we denote a measure of fit, such as the ridge loss function described above, being composed of $l(\beta)$ and a penalty function $pen_\lambda(\beta)$, then from a Bayesian perspective these correspond to the negative logarithms of the likelihood and the prior distribution, respectively. We can parameterize the BayesR dirac spike and slab model described above using the latent indicator of each SNP, j, $\gamma = (\gamma_j, \ldots, \gamma_p)^T$ with $\gamma_{j,l} = 0$ or 1, indicating whether or not the effect of SNP j follows a normal distribution with variance $\sigma_l^2 (l = 1, 2, 3, 4)$. Then $p(\gamma_{j,l} = 1|\pi_l) = \pi_l$ and the prior distribution of each SNP effect β_j conditional on the indicator $\gamma_{j,l}$ is

$$f(\beta_j|\gamma_{j,l}) = \begin{cases} \frac{1}{\sqrt{2\pi\sigma_l^2}}\exp(-\frac{\beta_j^2}{2\sigma_l^2}), & \text{if} \gamma_{j,l} = 1 \quad (l = 2, 3, 4) \\ \delta_0(\beta_j), & \text{if} \gamma_{j,l} = 0 \end{cases} \tag{30}$$

The joint distribution $p(\beta_j, \gamma_j)$ conditional on π_β is

$$\begin{aligned} f\left(\beta_j, \gamma_j|\pi_\beta, \sigma_\beta^2\right) &= \prod_{l=1}^4 f(\beta_j|\gamma_{j,l}) f(\gamma_{j,l} = 1|\pi_l) \\ &= (\delta_0(\beta_j)\pi_1)^{\gamma_{j,1}} \prod_{l=2}^4 \left(\frac{1}{\sqrt{2\pi\sigma_l^2}}\exp(-\frac{\beta_j^2}{2\sigma_l^2})\pi_l\right)^{\gamma_{j,l}} \end{aligned} \tag{31}$$

to simplify the following, we assume only a single normal distribution with $\pi_1 + \pi_2 = 1$ and we redefine the regression coefficient as $\beta_j = \gamma_j \alpha_j$ with $\alpha_j|\sigma_\beta^2 \sim N(0, \sigma_\beta^2)$. then:

$$\begin{aligned} f\left(\alpha_j, \gamma_j|\pi_\beta, \sigma_\beta^2\right) &= (\delta_0(\alpha_j)\pi_1)^{\gamma_{j,1}} \left(\frac{1}{\sqrt{2\pi\sigma_\beta^2}}\exp(-\frac{\alpha_j^2}{2\sigma_\beta^2})\pi_l\right)^{\gamma_{j,2}} \\ &= \pi_1^{\gamma_{j,1}}(1-\pi_1)^{\gamma_{j,2}}\frac{1}{\sqrt{2\pi\sigma_\beta^2}}\exp(-\frac{\alpha_j^2}{2\sigma_\beta^2}) \end{aligned} \tag{32}$$

Now as above, if we define an active set of markers, $\mathbf{X}_{\gamma\neq 0}$, as those columns of $\mathbf{X}$ where $\beta_{\gamma\neq 0}$, with an active set of γ, and $||\gamma||_0 = \sum_{j=1}^p \gamma_j$ be its cardinality. The joint prior on the vector γ, α then factorizes across all the markers as

$$\begin{aligned} f\left(\alpha, \gamma|\pi_\beta, \sigma_\beta^2\right) &= \prod_{j=1}^p f\left(\alpha_j, \gamma_j|\pi_\beta, \sigma_\beta^2\right) \\ &= \pi_1^{||\gamma||_0}(1-\pi_1)^{p-||\gamma||_0}(2\pi\sigma_\beta^2)^{-\frac{p}{2}}\exp\left\{-\frac{1}{2\sigma_\beta^2}\sum_{j=1}^p \alpha_j^2\right\} \end{aligned} \tag{33}$$

as above we can express the likelihood in terms of γ, α as

$$f\left(y|\gamma, \alpha, \pi_\beta, \sigma_\epsilon\right) = (2\pi\sigma_\epsilon^2)^{-\frac{n}{2}}\exp\left\{-\frac{1}{2\sigma_\epsilon^2}||y - \mathbf{X}_{\gamma\neq 0}\alpha_{\gamma\neq 0}||_2^2\right\} \tag{34}$$

and then under this reparamterisation the posterior is given as

$$\begin{aligned} f\left(\alpha, \gamma|\pi_\beta, \sigma_\beta^2, \sigma_\epsilon^2, y\right) &\propto f\left(\alpha, \gamma|\pi_\beta, \sigma_\beta^2\right) f\left(y|\gamma, \alpha, \pi_\beta, \sigma_\epsilon\right) \\ &\propto \exp\left\{\frac{1}{2\sigma_\epsilon^2}||y - \mathbf{X}_{\gamma\neq 0}\alpha_{\gamma\neq 0}||_2^2 - \frac{1}{2\sigma_\beta^2}||\alpha||_2^2 - log\left(\frac{1-\pi_1}{\pi_1}\right)||\gamma||_0\right\} \end{aligned} \tag{35}$$

The regularized maximum a posterior estimator is equivalent to minimising over γ, α the least squares objective function as

$$\min_{\gamma,\alpha}||y - \mathbf{X}_{\gamma\neq 0}\alpha_{\gamma\neq 0}||_2^2 + \lambda\|\alpha\|_2^2 + 2\sigma_\epsilon^2\log\left(\frac{1-\pi_1}{\pi_1}\right)||\gamma||_0 \tag{36}$$

In comparison to the ridge loss function described above, the first two terms are very similar and the third term imposes a sparsity constraint on the model. The term $\lambda\|\alpha\|_2^2$ has the same expectation as in Eq. (26) but with $\mathbf{X}$ replaced with $\mathbf{X}_{\gamma\neq 0}$. To give some insight into the influence of collinearity on $\mathbb{E}[||\gamma||]_0$ and on the active set, we explore a two SNP scenario.

In a single site updating scheme, the probability that the first marker enters the model is given by Eq. 10. We seek to derive the probability that the second marker enters the model conditional on the first marker being in the model. We consider a scenario where we observe our standardised outcome $\tilde{\mathbf{y_c}}$ and two correlated predictors $\mathbf{X}_1$ and $\mathbf{X}_2$. We assume that $\tilde{\mathbf{y_c}}$, $\mathbf{X}_1$ and $\mathbf{X}_2$ are scaled with zero mean and unit variance. We can then derive the partial least squares regression for $\tilde{\mathbf{y_c}}$ regressed on $\mathbf{X}_2$, adjusting for $\mathbf{X}_1$. If $\beta_{x_1,\tilde{y}} = \frac{\mathbf{X}_1^T\tilde{\mathbf{y}}}{\Sigma_{1,1}}$, with $\Sigma_{1,1} = \mathbf{X}_1^T\mathbf{X}_1 + \lambda_1\mathbf{I}$, then a residual vector $\boldsymbol{\epsilon}_{y_c,X_1} = \mathbf{y}_c - \beta_{x_1,\tilde{y_c}}\mathbf{X}_1$ is the vector left after backfitting $\beta_{x_1,\tilde{y_c}}$ and we define $\boldsymbol{\epsilon}_{X_1,X_2} = \mathbf{X}_2 - \rho_{X_1,X_2}\mathbf{X}_1$ as the additional information in X_2 left to fit $\beta_{x_2,\epsilon_{y_c,X_1}}$, with ρ_{X_1,X_2} the correlation of X_1 and X_2. The correlation between the two residuals $\boldsymbol{\epsilon}_{y_c,X_1}$ and $\boldsymbol{\epsilon}_{X_1,X_2}$ can be used to estimate $\beta_{x_2,\epsilon_{y_c,X_1}}$, since $\beta_{x_2,\epsilon_{y_c}} = \frac{N}{\Sigma_{1,l}}\rho_{\epsilon_{y_c,X_2}}$. The correlation is a ratio between a covariance and a variance as

$$\begin{aligned} Cov_{\boldsymbol{\epsilon}_{y_c,X_1},\boldsymbol{\epsilon}_{X_1,X_2}} &= \frac{1}{N}\sum\left(\mathbf{y}_c - \beta_{x_1,\tilde{y_c}}\mathbf{X}_1\right)\left(\mathbf{X}_2 - \rho_{X_1,X_2}\mathbf{X}_1\right) \\ &= \frac{1}{N}\sum\left(\mathbf{y}_c X_2 - \rho_{x_1,x_2}X_1\tilde{y_c} - \beta_{x_1,\tilde{y_c}}X_1X_2 + N\beta_{x_1,\tilde{y_c}}\rho_{X_1,X_2}\right) \\ &= \rho_{\epsilon_{y_c,X_2}} - \rho_{X_1,X_2}\beta_{x_1,\tilde{y_c}}\frac{\Sigma_{1,l}}{N} - \beta_{x_1,\tilde{y_c}}\rho_{X_1,X_2} + \beta_{x_1,\tilde{y_c}}\rho_{X_1,X_2} \\ &= \rho_{\epsilon_{y_c,X_2}} - \rho_{X_1,X_2}\beta_{x_1,\tilde{y_c}}\frac{\Sigma_{1,l}}{N} \\ &= \rho_{\epsilon_{y_c,X_2}} - \rho_{X_1,X_2}\frac{1}{N}X_1\tilde{y_c} \end{aligned} \tag{37}$$

The variance in the correlation denominator is $S^2_{\epsilon_{X_1,X_2}} = 1 - \rho^2_{X_1,X_2}$ which gives

$$\beta_{y_c,X_2|X_1} = \frac{N}{\Sigma_{2,l}} \times \frac{\rho_{\epsilon_{y_c,X_2}} - \rho_{X_1,X_2}\frac{1}{N}X_1\tilde{y_c}}{1-\rho^2_{X_1,X_2}} \tag{38}$$

Eq. 38 can then be used in Eq. 9 and Eq. 10 to determine the posterior inclusion probability of the second covariate conditional on the first covariate being in the model. From this, the expectation, $\mathbb{E}[\|\gamma\|_0]$ for a two SNP scenario is then

$$\begin{aligned}\mathbb{E}[\|\gamma\|_0] &= p(l_1 = 1|\boldsymbol{\theta},\mathbf{y}) + p(l_2 = 1|\boldsymbol{\theta},\mathbf{y}) \\ &= \frac{1}{1+\exp\left[log(\pi_0) - (-\frac{1}{2}[-\log(|\lambda\Sigma^{-1}_{1,1}|) - (\frac{\beta^2_{y_c,X_1}\Sigma_{1,1}}{\sigma^2_\beta})])\right]} \\ &+ \frac{1}{1+\exp\left[log(\pi_0) - (-\frac{1}{2}[-\log(|\lambda\Sigma^{-1}_{2,1}|) - (\frac{\beta_{y_c,X_2|X_1}\Sigma_{2,1}}{\sigma^2_\beta})])\right]}\end{aligned} \tag{39}$$

With the dirac spike and slab and ridge regression estimators minimizing the same sum-of-squares, the key difference with the constrained estimation formulation of ridge regression is not in the explicit form of λ but in what is bounded the domain of acceptable values for α. For the BayesR estimator the domain is specified by a bound on the $\ell_0 norm$ of the regression parameter, while for its ridge counterpart the bound is applied to the squared $\ell_2 norm$ of β. Multicollinearity will reduce the likelihood of the second covariate entering the model as it's inclusion is dependent upon ρ_{X_1,X_2} the correlation among covariates and $\rho_{\epsilon_{y_c},X_2}$ the correlation of the second marker and the residual vector after backfitting the first marker. This will limit the range of possible estimates to be lower than those obtained from ridge regression, reducing inflation of $\lambda\|\alpha\|^2_2$ under high collinearity, but not entirely removing it. Due to the sampling of markers from a series of normal distributions, collinearity will still inflate $\lambda\|\alpha\|^2_2$, however, the degree to which this occurs will depend upon the number of correlated markers, the degree of correlation among them and the strength of the effects. Therefore, our aim here is not to derive a general solution predictive of all situations, merely it is to highlight that in order to make some inference as to the underlying distribution of genetic effects, it is required to extend the model as outlined in the following section.

Small-scale simulation example

While we assess the performance of our model in the large-scale simulation work, smaller-scale focused simulation work was also conducted to support and test the inference made. Our theory suggests that there will be increased variance of the regression coefficient estimates and, as a result, an inflated estimate of the phenotypic variance attributable to SNP markers under high multicollinearity for both mixed linear model approaches and a Dirac spike and slab mixture model. To create a toy example of this, we conducted a simulation study where for each of 50 replicates, we simulated 50 independent genomic regions, each containing two SNP markers. In each simulation replicate, we simulated values for 5,000 individuals at each of the 50 SNP marker pairs, by first simulating from a standard multivariate normal distribution with correlation set to either 0 or 0.99. From this, we obtained the integral from $-\inf$ to q of the probability density function, where q is the z-score of the values obtained for each individual from the multivariate normal. From these integrals, we then made two draws from the inverse of the cumulative density function of the binomial distribution to obtain the marker value for each individual, with frequency 0.3. This gave marker values (0, 1, or 2), with the pairs of SNPs having either all LD = 0, or all LD = 0.99. For each of the 50 pairs of SNPs, we assigned effect size 0 to the first marker and 0.1 to the second marker. We then scaled the SNP markers to zero mean and unit variance and multiplied the markers by the effect sizes to obtain the genetic values for the 5,000 individuals, with variance 0.5. We then simulated the environmental component of the phenotype from a normal distribution with zero mean and variance 0.5 and then created a phenotype as the sum of the genetic values and the environmental values, with zero mean and unit variance.

We then analysed these 50 data sets using different methods of single-marker OLS regression (OLS), mixed-linear model association (MLMA), ridge regression (Ridge), and a Dirac spike and slab mixture of regressions model (BayesR), all of which are described above. For the frequentist approaches, we directly solved the estimation equations, scaling the SNP markers to have zero mean and unit variance. For BayesR we sampled the effects for 5000 iterations, with burn-in period of 2000 iterations to obtain the posterior mean effect sizes, again scaling the SNP markers to zero mean and unit variance. We repeated these analyses many times, each time fixing the estimated phenotypic variance attributable to the markers σ^2_G to be a

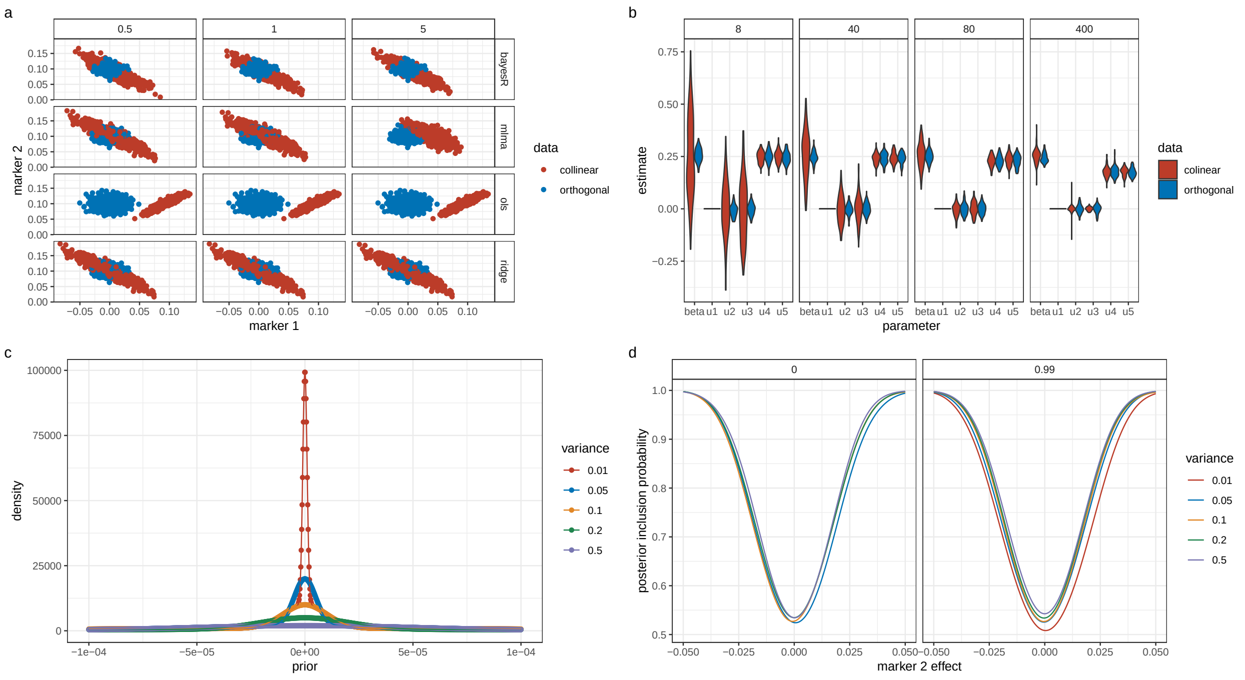

Supplementary Figure 17. Theory and simulation study of SNP marker model parameters. (a) accompanies Eq. (26) and shows the distribution of the point estimates of the effect sizes of two correlated markers of effect size (0,0.1) under orthogonality (LD = 0) and collinearity (LD = 0.99) across 2500 replicates (50 independent genomic regions for 5,000 individuals within each of 50 replicates) for a range of different models: a dirac spike and slab mixture of regressions model (bayesR), a mixed linear association model (MLMA), single-marker ordinary least squares (OLS), and ridge regression (Ridge). Panels give the lambda shrinkage parameter of the model, the error variance divided by the phenotypic variance attributable to the SNP markers, showing that as lambda decreases the variation of the estimates increases under multicollinearity. (b) accompanies Eq.(29) and shows the marker estimates obtained from Henderson's mixed model equations for a MLMA with the focal marker as fixed (beta) and random (u1), with four other markers in the model. Markers were either uncorrelated (orthogonal, LD=0) or the focal marker was correlated with the first two out of the four other markers (collinear, LD=0.99). Panels give the lambda shrinkage parameter, showing that as lambda decreases the variation of the estimates increases under multicollinearity. (c) shows the prior density of the BayesR model for different hyperparameter values of the phenotypic variance attributable to genetic effects (variance), showing that as the variance attributable to the markers decreases, the prior has higher mass around zero. Thus, with a grouped mixture of regressions model (BayesRR-RC), each hyperparameter estimate will be smaller and thus there will be higher prior density around zero. This then has consequences for marker inclusion in the BayesRR model. Higher prior mass around zero makes little difference for the inclusion of uncorrelated markers, but it results in reduced posterior inclusion probability for correlated markers as shown in (d). For (d), we calculated the inclusion probability (PIP) of two markers with LD = 0 and LD = 0.99, as the variance attributable to the SNP markers, and thus the prior distribution, changes assuming a background inclusion probability of 0.1, a sample size of 5000, and an effect size of 0.01 SD for marker 1 (see Methods). (d) shows that the PIP of the second marker is reduced across a range of possible effect size values (the average of 1000 replicated simulations for 1000 marker 2 effect values for each line) as the hyperparameter estimate decreases, and thus the smaller hyperparameter estimates in a BayesRR model means that correlated markers are less likely to enter the model, controlling better for the effects of multicollinearity.

different value. We selected (2, 1, 0.5, 0.1, and 0.01) and fixed the residual variance σ^2_ϵ to be 0.5, to give different lambda values $\lambda = \frac{\sigma^2_\epsilon}{\sigma^2_G}$, giving $\lambda = 0.25, 0.5, 1, 5,$ and 50. Our aim here was to explore the pattern of effect sizes that we obtain under these λ values. So first, we plotted the effect sizes obtained for each of the 50 SNP pairs obtained across the 50 simulation replicates in Supplementary Figure 17a, to show the differences in the variance of the estimates obtained across approaches when the pairs of SNP markers were orthogonal (LD=0), or collinear (LD=0.99), under different lambda values. Second, we then plot the distribution of the sum of the squared regression coefficients in Supplementary Figure 18c across approaches, when the pairs of SNP markers were orthogonal (LD=0), or collinear (LD=0.99), under different lambda values, where the expectation is 0.5 (sum of the 50 squared 0.1 SD effect sizes). This simulation confirmed, that regression coefficient estimates have higher variance under multicollinearity, resulting in inflation of the sum of the squared coefficient estimates for all approaches when the variation attributable to SNP markers is overestimated, resulting in a reduction in the lambda values.

We then further explored the performance of the MLMA and BayesR models under multicollinearity to (i)

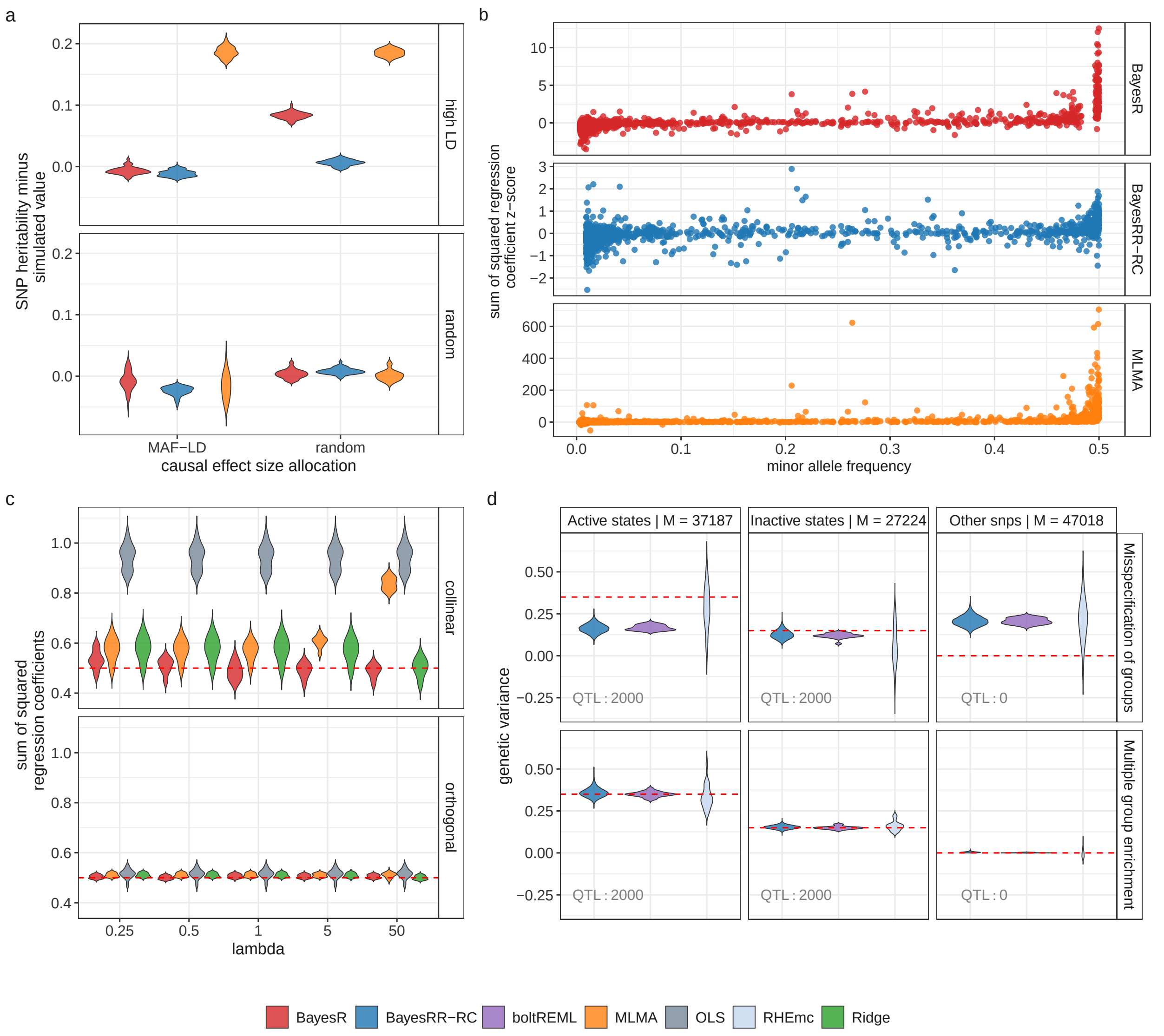

Supplementary Figure 18. Theory and simulation study for genetic penalized regression models under multicollinearity. (a) Smaller-scale simulation study then that presented in the main text using real genomic data from chromosome 22 where 50 replicate phenotypes were generated by either allocating 5000 LD-independent causal variants to high LD SNPs (y-axis panel: high LD), or randomly allocating 5000 SNPs as causal variants (y-axis panel: random), and then either randomly allocating effect sizes to those SNPs (x-axis: random), or allocating effect sizes proportional to their LD and MAF (x-axis: MAF-LD, see Methods). In this simulation every LD block of chromosome 22 contributes to the trait variance. SNP heritability estimation error is plotted as the difference of the estimate and the true simulated value across the 50 replicates. (b) We then investigated this further for the scenario where causal variants are allocated to high-LD SNPs. While the 5000 causal variants are LD-independent, they are each correlated with a large number of SNPs of simulated effect size 0. For each causal variant, we took all the markers in LD ≥ 0.05 and summed the squared estimated regression coefficients of these markers. The true simulated value is simply the square of the effect size allocated to the causal variant, and we subtracted this from the sum of the squared regression coefficients divided by the SD of the simulated genetic effects, to give a z-score for each causal variant and this is plotted on the y-axis for MLMA, BayesR, and BayesRR-RC. (c) Our theory outlines how this overestimation is the result of the effect of multicollinearity (see Methods) and an example is shown here, where 50 pairs of SNP markers with LD = 0.9 were simulated for each of 50 simulation replicates, where only one marker of each pair has an effect (0,0.1 SD), giving the sum of the squared regression coefficients as 0.5 for each simulation (dotted red line). lambda is the shrinkage parameter, the ratio of the error variance and the variance attributable to the SNP markers, used for MLMA, ridge regression (Ridge) and the BayesR model to estimate the effects. (d) Simulation of a genetic architecture (dotted red line) using real annotations from the Epigenome Roadmap Project [34] (active states, inactive states, other snps). We compared BayesRR-RC to other recent approaches providing annotation-specific variance component estimates in individual-level data when SNPs are randomly assigned to an annotation (labelled: misspecification of groups), or when specifying enrichment using prior knowledge (labelled: multiple group enrichment)

better understand the interplay between the fixed GLS estimate obtained and the random marker effects, and (ii) to better understand how the prior of the BayesR model changes with lambda and how this constrains the

inclusion probabilities of correlated markers. We first examined the influence of varying lambda and varying the collinearity of markers on the variation of the effect size estimates obtained from the Henderson's mixed model equations, where one focal marker is estimated as fixed, and a further five markers are estimated as random, with LD between the markers estimated as fixed and random. To do this, we simulated five markers in the same manner as described above that were either (i) entirely orthogonal with LD = 0, or (ii) had LD = 0.99 among the first three markers, with the final two markers having LD = 0 with all others. We assigned effect sizes to the five markers as $\beta = (0.25, 0, 0, 0.25, 0.25)$, multiplied these effect sizes by the simulated marker values scaled to zero mean and unit variance to create the genetic values, and then added an environmental component simulated from a normal distribution with mean zero and variance 1 minus the variance of the genetic values (0.1875) to give a phenotype with zero mean and unit variance. We directly solved the Henderson's mixed model equations, fixing the lambda value at different levels (the appropriate lambda from theory assuming orthogonal covariate would $= (1 - 0.1875)/0.1875 = 4.333$). We find that even with high shrinkage, a lambda value of almost 20 times greater than the theoretical orthogonal expectation is required to produce effect sizes under collinearity, with similar variance to those obtained under orthogonality (Supplementary Figure 17b).

For BayesR, we first explored the density of the posterior distribution by simulating draws from the prior as we change the variance attributable to the SNP markers. Supplementary Figure 17c shows these densities, revealing how the prior becomes strongly centred on zero and almost exponentially distributed as the variance becomes small. This is in contrast to the almost flat prior observed with high variance, which will do little to constrain effect size estimates toward zero. We then conducted 1000 simulation replicates of paired SNP markers for 10 different scenarios of variance attributable to the SNP markers of 0.01, 0.05, 0.1, 0.2, and 0.5, for pairs of SNPs with correlation of either 0 or 0.99. For each of these 10,000 data sets, we simulate a pair of SNPs for 5000 individuals, assuming error variance of 0.5, effect size for the first marker of 0.01 SD and then we simulated a sequence of 1000 different effect sizes from -0.05 to 0.05. Of these 10 million phenotypes and pairs of SNPs obtained, we then determine the posterior inclusion probability of the second marker, given that the first marker is in the model, with the effect size correctly estimated as 0.01, from the BayesR model derivations presented above. The lines presented in Supplementary Figure 17d go through the mean posterior inclusion probability of the second SNP marker across the 1000 simulation replicates, for each of the 1000 different effect sizes from -0.05 to 0.05 for marker 2, with a different colour for each scenario of the variance attributable to the SNP markers. The plot shows a reduction in the posterior inclusion probability of the second SNP marker as the variance attributable to the SNP markers decreases under multicollinearity. Thus, if the hyperparameter estimates of the variance contributed by markers is kept small, by having different hyperparameters for different groups of markers, then the BayesR model acts to constrain the inclusion of any additional correlated markers in the model.

Having confirmed our theory, we then conducted a further simulation study to replicate these observations using real genomic data. We randomly selected 50,000 individuals from the UK Biobank study data and used the imputed SNP data from chromosome 22 as supplied in the data release. We simulated phenotypes under contrasting generative models:

- We chose markers of high LD with other SNPs to be the causal variants and we assigned effects proportional to the LD score of those markers and their minor allele frequency. To do this, we first grouped the SNPs using the clumping procedure in Plink (see Code Availability) based on 1 - MAF, selecting the highest frequency variants and removing any variants with LD < 0.01, to obtain 4988 independent SNPs. For these 4988 SNPs we calculated the LD score of the markers. We then assigned effect sizes to these selected SNPs, drawing them from a single normal distribution with variance $\sim \text{LD\_score}^1 \text{MAF}^{-1}$. We multiplied these effect sizes by the simulated marker values scaled to zero mean and unit variance to create the genetic values with variance 0.5, and then added an environmental component simulated from a normal distribution with mean zero and variance 1 minus the variance of the genetic values to give a phenotype with zero mean and unit variance.

- We then took the same 4988 SNPs but assigned effect sizes to the markers at random from a normal distribution with zero mean and variance 0.5/4988. We multiplied these effect sizes by the simulated marker values scaled to zero mean and unit variance to create the genetic values with variance 0.5, and then added an environmental component simulated from a normal distribution with mean zero and variance 1 minus the variance of the genetic values to give a phenotype with zero mean and unit variance.

- We then sampled randomly 4988 evenly spaced markers as causal variants, but assigned effect sizes proportional to the LD score and minor allele frequency of the markers as described above. We

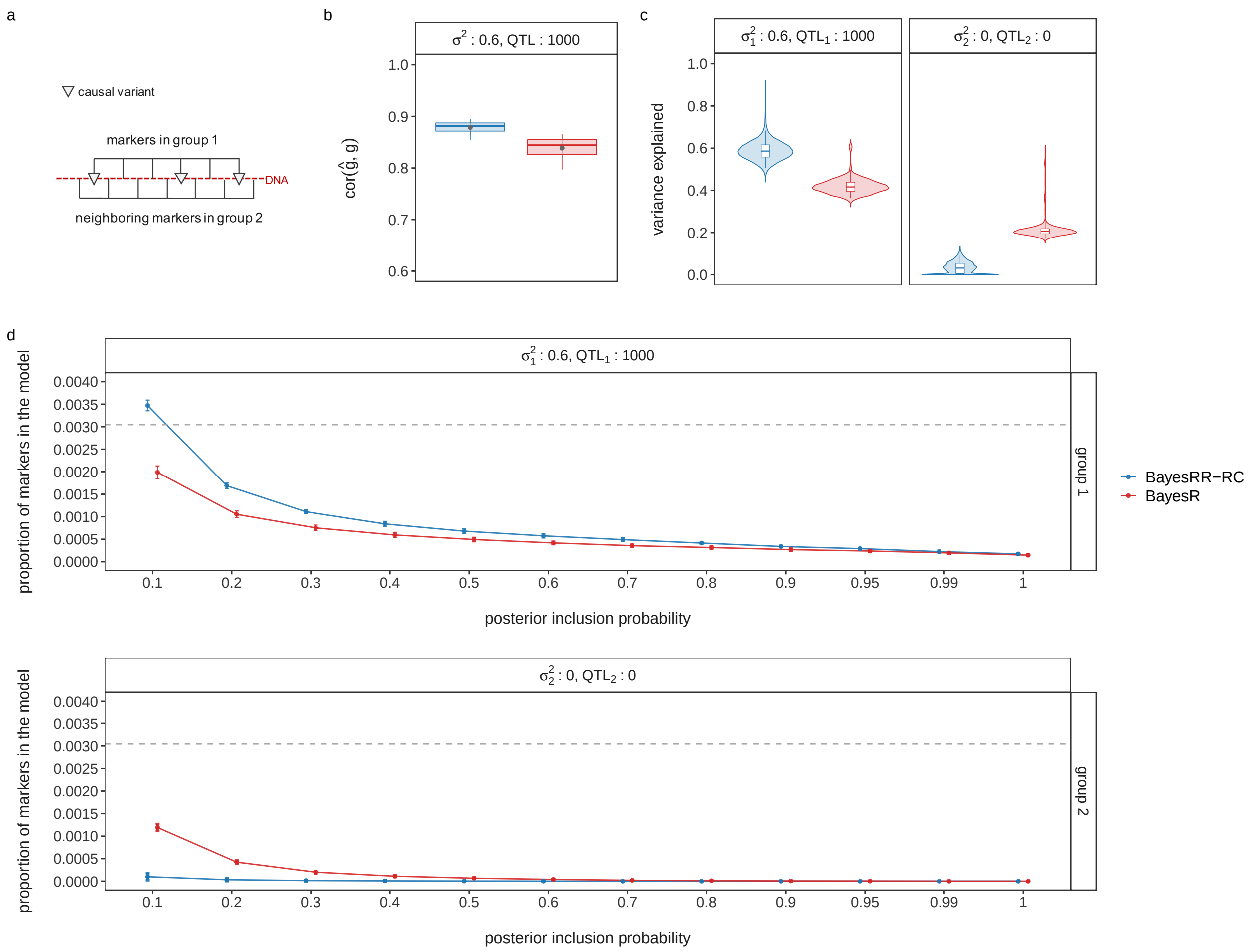

Supplementary Figure 19. Classification power of BayesRR-RC. Grouping effects in a BayesRR-RC model improves the power of BayesR to estimate effect sizes and infer the genetic architecture of common complex traits and diseases. This setting compares 10 simulations of 5 chains with different starting values (chain length : 2500, burn-in : 500, thin : 5) executed using BayesRR-RC. (a) Each simulation has two groups in high LD with an interdigitated structure where one in two SNPs is assigned to group 1 and all genetic variance is assigned to group 1 with 1000 QTL. Annotation-specific estimates for BayesR are calculated post-analysis for each group. (b) Estimation of markers effects in an independent data set. BayesRR-RC improves on correlation between predicted and simulated genetic values. This increase in prediction implies that adding functional information to BayesR better fits the data and improves prediction accuracy. (c) Genetic variance and (d) proportion of markers entering the model at posterior inclusion probability (pip) thresholds summarized across 10 simulations for group 1 and group 2. The proportion of markers included in the model is closer to the truth (dotted grey line) when using BayesRR-RC compared to BayesR. Effects are thus more likely attributed to the correct group using our approach, which also explains why we estimate more accurately the group genetic variance compared to the baseline. Simulation setting: $N = 20,000$ unrelated European individuals from the UK Biobank, $M = 328,385$ markers (chromosome 2). Dots in box plots show the mean of the correlation between predicted and simulated genetic values.

multiplied these effect sizes by the simulated marker values scaled to zero mean and unit variance to create the genetic values with variance 0.6, and then added an environmental component simulated from a normal distribution with mean zero and variance 1 minus the variance of the genetic values to give a phenotype with zero mean and unit variance.

- Finally, we then sampled randomly 4988 evenly spaced markers as causal variants and randomly assigned the effect sizes from a normal distribution with zero mean and variance 0.5/4988. We multiplied these effect sizes by the simulated marker values scaled to zero mean and unit variance to create the genetic values with variance 0.5, and then added an environmental component simulated from a normal distribution with mean zero and variance 1 minus the variance of the genetic values to give a phenotype with zero mean and unit variance.

This replicates our main simulation study, but creates a situation where there is an association at every LD block on chromosome 22 and thus the results seen in the main simulation study should be magnified

here. We analysed 50 simulation replicates of each of the four scenarios with BayesR, BayesRR-RC with 20 MAF-LD groups (deciles of MAF, each split into two groups based on median LD score within each MAF decile), and a MLMAi model implemented in software GCTA. For the Bayesian methods we ran three chains with different starting values for each of the 200 simulation replicates for 3000 iterations, removing the first 1500 iterations as burn-in and taking the posterior mean across the three chains. In Supplementary Figure 18a we plot the distribution of the posterior mean for BayesR and BayesRR-RC, and the MLMA point estimates, of the proportion of variance attributable to the SNP markers minus the true simulated value obtained across the 50 simulation replicates for each of the four scenarios, showing inflation of the MLMA estimates when selecting high LD variants, and inflation of the BayesR estimates with high LD and random effect size estimates. In contrast, estimates obtained from BayesRR-RC were unbiased across all scenarios. By simulating an effect size MAF relationship $\sim \text{LDscore}^1\text{MAF}^{-1}$, we are assigning the smallest absolute effect size values to the most common SNPs, which appears to limit the inflation of the estimates for BayesR, when selecting high LD SNPs as causal variants (Supplementary Figure 18a). We then examined the effect size estimates obtained from these three approaches across the MAF spectrum under the second scenario of high LD causal variant selection, but random effect size allocation, to show using z-scores calculated as the estimated effects minus the simulated effects, divided by the SD of the simulated effects. We find overestimation of common variant effect sizes under BayesR, and dramatic inflation of effect size estimates under MLMA showing poor recovery of the underlying effect size distribution (Supplementary Figure 18b). Grouping effects by MAF and LD in a BayesRR-RC model resolved this overestimation issue (Supplementary Figure 18b) as seen in our original large-scale simulation study.

We then explore the ability of the model to recover a different set of annotation-specific effect sizes using the same set of 50,000 randomly selected UK Biobank individuals and imputed genotype data for chromosome 22 grouped by chromatin state annotations (15-state ChromHMM model) from the epigenome of primary mononuclear cells from peripheral blood (E062) of the Epigenome Roadmap Project [34]. We simulated the genetic architecture as follows :

- We first mapped SNPs to active and inactive chromatin states from the mnemonic bed files for E062 (see Code availability). 37,187 SNPs mapped to active chromatin states including transcription start site (TSS) and their flanking regions, genic and other enhancers, untranslated transcribed regions (UTR) and actively transcribed regions and zinc finger genes states. 27,224 SNPs mapped to inactive states including heterochromatin, bivalent/poised TSS and their flanking regions, bivalent enhancers and repressed polycomb states. The remaining 47,018 SNPs were grouped and labelled as Other SNPs.
- To simulate enrichment in both chromatin states, we randomly sampled 2000 SNPs as causal variants from variants mapped to active chromatin states and another 2000 SNPs from variants mapped to inactive chromatin states. We then assigned effect sizes to these 4000 selected SNPs, drawing them from a normal distribution with zero mean and variance 0.35/2000 for active states and 0.15/2000 for inactive states.
- We multiplied annotation-specific effect sizes by the simulated marker values scaled to zero mean and unit variance to create the annotation-specific genetic values with variance 0.35 for active states, 0.15 for inactive states and 0 for other SNPs. We finally added an environmental component simulated from a normal distribution with mean zero and variance 1 minus 0.5 (the sum of the genetic values) to give a phenotype with zero mean and unit variance.

We analyzed 20 simulation replicates with our BayesRR-RC software specfiying annotations (active states, inactive states and other SNPs) with 2 LD groups based on median LD score within each annotation. We compared our software to BoltREML [35] and RHEmc [36] both multi-variance component methods that also use individual-level data but provide single heritability estimates per genetic component. For BayesRR-RC we ran three chains with different starting values for each of the 20 simulations replicates for 3000 iterations, removing the first 1000 iterations as burn-in and taking the posterior mean across the three chains. We then performed the same analysis but randomly assigning SNPs to each annotation resulting in mis-specification of the underlying genetic architecture. In Supplementary Figure 18d, we plot the estimated sum of the squared regression coefficients that is evenly split across the three annotations when misspecifying the underlying genetic architecture (labelled : Misspecification of groups) and shows enrichment when we properly assign SNPs to annotation (labelled : Multiple group enrichment). We find that BayesRR-RC performs as BoltREML and RHEmc, with RHEmc estimates showing higher variability, supporting our main simulation results.

We also further examined the ability of BayesRR-RC to recover effect sizes compared to BayesR by comparing 10 simulations of 5 chains with different starting values where each simulation has two groups in high LD with an interdigitated structure where one in two SNPs is assigned to group 1 (Supplementary Figure 19a). We then simulated phenotypes as previously described, randomly selecting 1000 causal variants in group 1 only, using 20,000 randomly selected UK Biobank individuals and imputed genotype data for chromosome 2 (with MAF > 0.05). In Supplementary Figure 19, we compare the proportion of markers entering the model in group 1 and group 2 at different posterior inclusion probability thresholds. Annotation-specific estimates for BayesR are calculated post-analysis for each group. We also compare the correlation of estimated genetic values with the truth when using BayesRR-RC and BayesR. For this, we conducted estimation of marker effects in an independent data set to compare prediction accuracy. We simulated 10 new phenotypes and computed the genetic value $\hat{g} = X\hat{\beta}$ where X is the genotype matrix and $\hat{\beta}$ is a vector of estimated marker effects for each individual. Supplementary Figure 19 shows BayesRR-RC has improved model performance over BayesR to recover effect sizes and infer underlying genetic architectures.

Supplementary References

1. Edward I George and Robert E McCulloch. Variable selection via gibbs sampling. *Journal of the American Statistical Association*, 88(423):881–889, 1993.

2. Gertraud Malsiner-Walli and Helga Wagner. Comparing spike and slab priors for bayesian variable selection. *Austrian Journal of Statistics*, 40(4):241–264, Feb. 2016.

3. M. Erbe, B. J. Hayes, L. K. Matukumalli, S. Goswami, P. J. Bowman, C. M. Reich, B. A. Mason, and M. E. Goddard. Improving accuracy of genomic predictions within and between dairy cattle breeds with imputed high-density single nucleotide polymorphism panels. *Journal of Dairy Science*, 95(7):4114–4129, 2020/05/10 2012.

4. Gerhard Moser, Sang Hong Lee, Ben J. Hayes, Michael E. Goddard, Naomi R. Wray, and Peter M. Visscher. Simultaneous discovery, estimation and prediction analysis of complex traits using a bayesian mixture model. *PLOS Genetics*, 11(4):1–22, 04 2015.

5. Gemma E. Moran, Veronika Ročková, and Edward I. George. Variance prior forms for high-dimensional bayesian variable selection. *Bayesian Anal.*, 14(4):1091–1119, 12 2019.

6. Andrew Gelman et al. Prior distributions for variance parameters in hierarchical models (comment on article by browne and draper). *Bayesian Analysis*, 1(3):515–534, 2006.

7. Ismaël Castillo, Johannes Schmidt-Hieber, Aad Van der Vaart, et al. Bayesian linear regression with sparse priors. *The Annals of Statistics*, 43(5):1986–2018, 2015.

8. Daniel Trejo Banos, Daniel L McCartney, Marion Patxot, Lucas Anchieri, Thomas Battram, Colette Christiansen, Ricardo Costeira, Rosie M Walker, Stewart W Morris, Archie Campbell, et al. Bayesian reassessment of the epigenetic architecture of complex traits. *Nature Communications*, 11(1):1–14, 2020.

9. Stuart Geman and Donald Geman. Stochastic relaxation, gibbs distributions, and the bayesian restoration of images. In *Readings in Computer Vision*, pages 564–584. Elsevier, 1987.

10. Yali Amit and Ulf Grenander. Comparing sweep strategies for stochastic relaxation. *Journal of Multivariate Analysis*, 37(2):197–222, 1991.

11. M. Goddard. Genomic selection: prediction of accuracy and maximisation of long term response. *Genetica*, 136:245 EP –, 08 2009.

12. Trevor Park and George Casella. The bayesian lasso. *Journal of the American Statistical Association*, 103(482):681–686, 2008.

13. Carlos M Carvalho, Nicholas G Polson, and James G Scott. The horseshoe estimator for sparse signals. *Biometrika*, 97(2):465–480, 2010.

14. Andrew Gelman, Daniel Lee, and Jiqiang Guo. Stan: A probabilistic programming language for bayesian inference and optimization. *Journal of Educational and Behavioral Statistics*, 40(5):530–543, 2015.

15. Martín Abadi, Ashish Agarwal, Paul Barham, Eugene Brevdo, Zhifeng Chen, Craig Citro, Greg S. Corrado, Andy Davis, Jeffrey Dean, Matthieu Devin, Sanjay Ghemawat, Ian Goodfellow, Andrew Harp, Geoffrey Irving, Michael Isard, Yangqing Jia, Rafal Jozefowicz, Lukasz Kaiser, Manjunath Kudlur, Josh Levenberg, Dandelion Mané, Rajat Monga, Sherry Moore, Derek Murray, Chris Olah, Mike Schuster, Jonathon Shlens, Benoit Steiner, Ilya Sutskever, Kunal Talwar, Paul Tucker, Vincent Vanhoucke, Vijay Vasudevan, Fernanda Viégas, Oriol Vinyals, Pete Warden, Martin Wattenberg, Martin Wicke, Yuan Yu, and Xiaoqiang Zheng. TensorFlow: Large-scale machine learning on heterogeneous systems, 2015. Software available from tensorflow.org.

16. John Salvatier, Thomas V. Wiecki, and Christopher Fonnesbeck. Probabilistic programming in python using PyMC3. *PeerJ Computer Science*, 2:e55, apr 2016.

17. James Johndrow, Paulo Orenstein, and Anirban Bhattacharya. Scalable approximate mcmc algorithms for the horseshoe prior. *Journal of Machine Learning Research*, 21(73):1–61, 2020.

18. Bala Rajaratnam, Doug Sparks, Kshitij Khare, and Liyuan Zhang. Uncertainty quantification for modern high-dimensional regression via scalable bayesian methods. *Journal of Computational and Graphical Statistics*, 28(1):174–184, 2019.

19. Matthew Johnson, James Saunderson, and Alan Willsky. Analyzing hogwild parallel gaussian gibbs sampling. In C. J. C. Burges, L. Bottou, M. Welling, Z. Ghahramani, and K. Q. Weinberger, editors, *Advances in Neural Information Processing Systems 26*, pages 2715–2723. Curran Associates, Inc., 2013.

20. Elaine Angelino, Matthew James Johnson, Ryan P Adams, et al. Patterns of scalable bayesian inference. *Foundations and Trends® in Machine Learning*, 9(2-3):119–247, 2016.

21. Daniel Gianola. Priors in whole-genome regression: The bayesian alphabet returns. *Genetics*, 194(3):573–596, 2013.

22. Matthew Stephens and David J. Balding. Bayesian statistical methods for genetic association studies. *Nature Reviews Genetics*, 10(10):681–690, 2009.

23. Rohan Fernando, Ali Toosi, Anna Wolc, Dorian Garrick, and Jack Dekkers. Application of whole-genome prediction methods for genome-wide association studies: a bayesian approach. *Journal of Agricultural, Biological and Environmental Statistics*, 22(2):172–193, 2017.

24. Arthur E. Hoerl and Robert W. Kennard. Ridge regression: Biased estimation for nonorthogonal problems. *Technometrics*, 12(1):55–67, 1970.

25. C. M. Theobald. Generalizations of mean square error applied to ridge regression. *Journal of the Royal Statistical Society. Series B (Methodological)*, 36(1):103–106, 1974.

26. Robert M. Maier, Zhihong Zhu, Sang Hong Lee, Maciej Trzaskowski, Douglas M. Ruderfer, Eli A. Stahl, Stephan Ripke, Naomi R. Wray, Jian Yang, Peter M. Visscher, and Matthew R. Robinson. Improving genetic prediction by leveraging genetic correlations among human diseases and traits. *Nature Communications*, 9(1):989, 2018.

27. Jian Yang, Andrew Bakshi, Zhihong Zhu, Gibran Hemani, Anna AE Vinkhuyzen, Sang Hong Lee, Matthew R Robinson, John RB Perry, Ilja M Nolte, Jana V van Vliet-Ostaptchouk, et al. Genetic variance estimation with imputed variants finds negligible missing heritability for human height and body mass index. *Nature Genetics*, 47(10):1114, 2015.

28. Luke M Evans, Rasool Tahmasbi, Scott I Vrieze, Gonçalo R Abecasis, Sayantan Das, Steven Gazal, Douglas W Bjelland, Teresa R De Candia, Michael E Goddard, Benjamin M Neale, et al. Comparison of methods that use whole genome data to estimate the heritability and genetic architecture of complex traits. *Nature Genetics*, 50(5):737–745, 2018.

29. Doug Speed, Na Cai, Michael R Johnson, Sergey Nejentsev, David J Balding, UCLEB Consortium, et al. Reevaluation of snp heritability in complex human traits. *Nature Genetics*, 49(7):986, 2017.

30. Doug Speed, John Holmes, and David J Balding. Evaluating and improving heritability models using summary statistics. *Nature Genetics*, 52(4):458–462, 2020.

31. Kangcheng Hou, Kathryn S Burch, Arunabha Majumdar, Huwenbo Shi, Nicholas Mancuso, Yue Wu, Sriram Sankararaman, and Bogdan Pasaniuc. Accurate estimation of snp-heritability from biobank-scale data irrespective of genetic architecture. *Nature Genetics*, page 1, 2019.

32. C.R. Henderson. Best linear unbiased prediction of breeding values not in the model for records. *Journal of Dairy Science*, 60(5):783 – 787, 1977.

33. Jian Yang, Noah A Zaitlen, Michael E Goddard, Peter M Visscher, and Alkes L Price. Advantages and pitfalls in the application of mixed-model association methods. *Nature Genetics*, 46(2):100–106, 2014.

34. Anshul Kundaje, Wouter Meuleman, Jason Ernst, Misha Bilenky, Angela Yen, Alireza Heravi-Moussavi, Pouya Kheradpour, Zhizhuo Zhang, Jianrong Wang, Michael J Ziller, et al. Integrative analysis of 111 reference human epigenomes. *Nature*, 518(7539):317, 2015.

35. Po-Ru Loh, Gaurav Bhatia, Alexander Gusev, Hilary K Finucane, Brendan K Bulik-Sullivan, Samuela J Pollack, Teresa R de Candia, Sang Hong Lee, Naomi R Wray, Kenneth S Kendler, et al. Contrasting genetic architectures of schizophrenia and other complex diseases using fast variance-components analysis. *Nature Genetics*, 47(12):1385, 2015.

36. Ali Pazokitoroudi, Yue Wu, Kathryn S Burch, Kangcheng Hou, Bogdan Pasaniuc, and Sriram Sankararaman. Scalable multi-component linear mixed models with application to snp heritability estimation. *bioRxiv*, page 522003, 2019.
