# Supplementary material for: Probabilistic inference of the genetic architecture underlying functional enrichment of complex traits
Source: Nat Commun. 2021 Nov 30;12:6972. doi: 10.1038/s41467-021-27258-9 (PMC8633298; doi:10.1038/s41467-021-27258-9)
Supplement: Supplementary file 3 — Description of Additional Supplementary Files [file 41467_2021_27258_MOESM3_ESM.pdf]

Description of Additional Supplementary Files

File Name: Supplementary Data 1
Description: SNP partitioned into 13 annotation groups.

File Name: Supplementary Data 2
Description: SNP heritability attributable to each genomic annotation and phenotype.

File Name: Supplementary Data 3
Description: SNP heritability estimates from RHE-mc.

File Name: Supplementary Data 4
Description: SNP heritability estimates from stratified-LDSC.

File Name: Supplementary Data 5
Description: SNP heritability estimates from SumHer.

File Name: Supplementary Data 6
Description: Mean effect sizes of gene components for exons contributing to the phenotypic variance with > 95% probability.

File Name: Supplementary Data 7
Description: Mean effect sizes of gene components for introns contributing to the phenotypic variance with > 95% probability.

File Name: Supplementary Data 8
Description: Mean effect sizes of gene components for 1kb regions contributing to the phenotypic variance with > 95% probability.

File Name: Supplementary Data 9
Description: Mean effect sizes of gene components for cis regions contributing to the phenotypic variance with > 95% probability.

File Name: Supplementary Data 10
Description: Contribution of SNPs with posterior inclusion probability (PIP) > 0.95 to each phenotype and corresponding p-value from UKB GWAS summary statistics (see Code Availability).
