# Supplementary material for: Probabilistic inference of the genetic architecture underlying functional enrichment of complex traits
Source: Nat Commun. 2021 Nov 30;12:6972. doi: 10.1038/s41467-021-27258-9 (PMC8633298; doi:10.1038/s41467-021-27258-9)
Supplement: Supplementary file 5 — Reporting Summary [file 41467_2021_27258_MOESM5_ESM.pdf]

nature research

Corresponding author(s): Matthew Robinson

Last updated by author(s): Oct 21, 2021

Reporting Summary

Nature Research wishes to improve the reproducibility of the work that we publish. This form provides structure for consistency and transparency in reporting. For further information on Nature Research policies, see our Editorial Policies and the Editorial Policy Checklist.

Statistics

For all statistical analyses, confirm that the following items are present in the figure legend, table legend, main text, or Methods section.

n/a	Confirmed	
☐	☒	The exact sample size (*n*) for each experimental group/condition, given as a discrete number and unit of measurement
☐	☒	A statement on whether measurements were taken from distinct samples or whether the same sample was measured repeatedly
☐	☒	The statistical test(s) used AND whether they are one- or two-sided<br>*Only common tests should be described solely by name; describe more complex techniques in the Methods section.*
☐	☒	A description of all covariates tested
☐	☒	A description of any assumptions or corrections, such as tests of normality and adjustment for multiple comparisons
☐	☒	A full description of the statistical parameters including central tendency (e.g. means) or other basic estimates (e.g. regression coefficient) AND variation (e.g. standard deviation) or associated estimates of uncertainty (e.g. confidence intervals)
☐	☒	For null hypothesis testing, the test statistic (e.g. *F*, *t*, *r*) with confidence intervals, effect sizes, degrees of freedom and *P* value noted<br>*Give P values as exact values whenever suitable.*
☐	☒	For Bayesian analysis, information on the choice of priors and Markov chain Monte Carlo settings
☐	☒	For hierarchical and complex designs, identification of the appropriate level for tests and full reporting of outcomes
☐	☒	Estimates of effect sizes (e.g. Cohen's *d*, Pearson's *r*), indicating how they were calculated

Our web collection on statistics for biologists contains articles on many of the points above.

Software and code

Policy information about availability of computer code

Data collection	No software was used in the Data collection.

Data analysis

Our BayesRR-RC model is implemented within the software GMRM, with full open source code available at: https://github.com/medical-genomics-group/gmrm.

UCSC Table Browser https://genome.ucsc.edu/cgi-bin/hgTables

flashPCA https://github.com/gabraham/flashpca

Plink1.90 https://www.cog-genomics.org/plink2/

GCTA https://cnsgenomics.com/content/software

HACER database http://bioinfo.vanderbilt.edu/AE/HACER/

snp2tfbs database https://ccg.epfl.ch//snp2tfbs/

fastGWA database http://fastgwa.info/ukbimp/phenotypes/

Computing environment https://www.epfl.ch/research/facilities/scitas/hardware/helvetios/

For manuscripts utilizing custom algorithms or software that are central to the research but not yet described in published literature, software must be made available to editors and reviewers. We strongly encourage code deposition in a community repository (e.g. GitHub). See the Nature Research guidelines for submitting code & software for further information.

Data

Policy information about availability of data

All manuscripts must include a data availability statement. This statement should provide the following information, where applicable:

- Accession codes, unique identifiers, or web links for publicly available datasets
- A list of figures that have associated raw data
- A description of any restrictions on data availability

This project uses UK Biobank data under project 35520. The Estonian Genome Centre data are protected and are not available due to data privacy laws. The Estonian Genome Centre data can be made available under restricted access upon request from the cohort author R.M. with appropriate research agreements. Summaries of all posterior distributions generated in this study are provided in Supplementary Data tables. Full posterior distributions of the SNP marker effects sizes and estimated variance components for each trait are deposited on Dryad with DOI: https://doi.org/10.5061/dryad.sqv9s4n51

The following publicly available databases were used:

UCSC Table Browser https://genome.ucsc.edu/cgi-bin/hgTables

HACER database http://bioinfo.vanderbilt.edu/AE/HACER/

snp2tfbs database https://ccg.epfl.ch//snp2tfbs/

fastGWA database http://fastgwa.info/ukbimp/phenotypes/

Field-specific reporting

Please select the one below that is the best fit for your research. If you are not sure, read the appropriate sections before making your selection.

☒ Life sciences ☐ Behavioural & social sciences ☐ Ecological, evolutionary & environmental sciences

For a reference copy of the document with all sections, see nature.com/documents/nr-reporting-summary-flat.pdf

Life sciences study design

All studies must disclose on these points even when the disclosure is negative.

Sample size

We restricted our discovery analysis of the UK Biobank to a sample of European-ancestry individuals. To infer ancestry, we used both self-reported ethnic background (UK Biobank data code 21000-0) selecting coding 1 and genetic ethnicity (UK Biobank data code 22006-0) selecting coding 1. We also took the 488,377 genotyped participants and projected them onto the first two genotypic principal components (PC) calculated from 2,504 individuals of the 1,000 Genomes project with known ancestries. Using the obtained PC loadings, we then assigned each participant to the closest population in the 1000 Genomes data: European, African, East-Asian, South-Asian or Admixed, selecting individuals with PC1 projection < absolute value 4 and PC 2 projection < absolute value 3. This gave a sample size of 456,426 individuals.

To facilitate contrasting the genetic basis of different phenotypes, we then removed closely related individuals as identified in the UK Biobank data release. While the BayesRR model can accommodate relatedness similar to mixed linear models, we wished to simply compare phenotypes at markers that enter the model due to LD with underlying causal variants. Relatedness leads to the addition of markers within the model to capture the phenotypic covariance of closely related individuals, and this will vary across traits in accordance with the genetic and environmental covariance for each phenotype. For these unrelated individuals, we used the imputed autosomal genotype data of the UK Biobank provided as part of the data release. We used the genotype probabilities to hard-call the genotypes for variants with an imputation quality score above 0.3. The hard-call-threshold was 0.1, setting the genotypes with probability <0.9 as missing. From the good quality markers (with missingness less than 5\% and p-value for Hardy-Weinberg test larger than 10-6, as determined in the set of unrelated Europeans) were selected those with minor allele frequency (MAF) > 0.0002 and rs identifier, in the set of European-ancestry participants, providing a data set 9,144,511 SNPs, short indels and large structural variants. From this we took the overlap with the Estonian Genome centre data to give a final set of 8,430,446 markers. From the UK Biobank European data set, samples were excluded if in the UKB quality control procedures they (i) were identified as extreme heterozygosity or missing genotype outliers; (ii) had a genetically inferred gender that did not match the self-reported gender; (iii) were identified to have putative sex chromosome aneuploidy; (iv) were excluded from kinship inference. Information on individuals who had withdrawn their consent for their data to be used was also removed. These filters resulted in a data set with 382,466 individuals. This sample size was chosen as it represents the largest single biobank data available to date providing the most appropriate data source for the testing of novel individual-level methodology.

We then selected the recorded measures of BMI (UK Biobank variable identifier 21001-0.0) and height (variable identifier 50-0.0) collected during initial assessment visit (year 2006-2010). BMI and height phenotypes 6 standard deviations (SD) away from the mean were not included in the analyses. For Type 2 Diabetes (T2D) in UKB we selected as cases very broadly as individuals who have main or secondary diagnosis (UKB fields 41202-0.0 - 41202-0.379 and 41204-0.0 - 41204-0.434) of “non-insulin-dependent diabetes mellitus” (ICD 10 code E11) or self-reported non-cancer illness (UKB field 20002-0.0 - 20002-2.28) “type 2 diabetes” (code 1223). From respondents self-reporting just “diabetes” (code 1220), we selected as cases those who did not self-report “type 1 diabetes” (code 1222) and had no Type 1 Diabetes (ICD code E10) diagnosis. Individuals with self-reported “diabetes” and “type 1 diabetes”/E10 were also left out from controls. We also defined coronary artery disease (CAD) cases broadly as participants with one of the following primary or secondary diagnoses or cause of death: ICD 10 codes I20 to I28; self-reported angina (code 1074) or self-reported heart attack/myocardial infarction (code 1075). Participants with self-reported “heart/cardiac problem” (code 1066) were not included as cases but also excluded from controls. This gave a sample size for each trait of 25,773 T2D cases and 359,730 T2D controls, 39,766 CAD cases and 344,054 CAD controls, 382,402 measures of height and 381,899 measures of BMI.

Data exclusions

We restricted our discovery analysis of the UK Biobank to a sample of European-ancestry individuals. To infer ancestry, we used both self-reported ethnic background (UK Biobank data code 21000-0) selecting coding 1 and genetic ethnicity (UK Biobank data code 22006-0) selecting coding 1. We also took the 488,377 genotyped participants and projected them onto the first two genotypic principal components (PC) calculated from 2,504 individuals of the 1,000 Genomes project with known ancestries. Using the obtained PC loadings, we then assigned each participant to the closest population in the 1000 Genomes data: European, African, East-Asian, South-Asian or Admixed, selecting individuals with PC1 projection < absolute value 4 and PC 2 projection < absolute value 3. This gave a sample size of 456,426 individuals.

To facilitate contrasting the genetic basis of different phenotypes, we then removed closely related individuals as identified in the UK Biobank data release. While the BayesRR model can accommodate relatedness similar to mixed linear models, we wished to simply compare phenotypes at markers that enter the model due to LD with underlying causal variants. Relatedness leads to the addition of markers within the model to capture the phenotypic covariance of closely related individuals, and this will vary across traits in accordance with the genetic and environmental covariance for each phenotype. For these unrelated individuals, we used the imputed autosomal genotype data of the UK Biobank provided as part of the data release. We used the genotype probabilities to hard-call the genotypes for variants with an imputation quality score above 0.3. The hard-call-threshold was 0.1, setting the genotypes with probability <0.9 as missing. From the good quality markers (with missingness less than 5\% and p-value for Hardy-Weinberg test larger than 10-6, as determined in the set of unrelated Europeans) were selected those with minor allele frequency (MAF) > 0.0002 and rs identifier, in the set of European-ancestry participants, providing a data set 9,144,511 SNPs, short indels and large structural variants. From this we took the overlap with the Estonian Genome centre data to give a final set of 8,430,446 markers. From the UK Biobank European data set, samples were excluded if in the UKB quality control procedures they (i) were identified as extreme heterozygosity or missing genotype outliers; (ii) had a genetically inferred gender that did not match the self-reported gender; (iii) were identified to have putative sex chromosome aneuploidy; (iv) were excluded from kinship inference. Information on individuals who had withdrawn their consent for their data to be used was also removed. These filters resulted in a data set with 382,466 individuals. This sample size was chosen as it represents the largest single biobank data available to date providing the most appropriate data source for the testing of novel individual-level methodology.

We then selected the recorded measures of BMI (UK Biobank variable identifier 21001-0.0) and height (variable identifier 50-0.0) collected during initial assessment visit (year 2006-2010). BMI and height phenotypes 6 standard deviations (SD) away from the mean were not included in the analyses. For Type 2 Diabetes (T2D) in UKB we selected as cases very broadly as individuals who have main or secondary diagnosis (UKB fields 41202-0.0 - 41202-0.379 and 41204-0.0 - 41204-0.434) of “non-insulin-dependent diabetes mellitus” (ICD 10 code E11) or self-reported non-cancer illness (UKB field 20002-0.0 - 20002-2.28) “type 2 diabetes” (code 1223). From respondents self-reporting just “diabetes” (code 1220), we selected as cases those who did not self-report “type 1 diabetes” (code 1222) and had no Type 1 Diabetes (ICD code E10) diagnosis. Individuals with self-reported “diabetes” and “type 1 diabetes”/E10 were also left out from controls. We also defined coronary artery disease (CAD) cases broadly as participants with one of the following primary or secondary diagnoses or cause of death: ICD 10 codes I20 to I28; self-reported angina (code 1074) or self-reported heart attack/myocardial infarction (code 1075). Participants with self-reported “heart/cardiac problem” (code 1066) were not included as cases but also excluded from controls. This gave a sample size for each trait of 25,773 T2D cases and 359,730 T2D controls, 39,766 CAD cases and 344,054 CAD controls, 382,402 measures of height and 381,899 measures of BMI. All of the exclusion criteria described here were predetermined prior to analysis and was conducted to maximize the available sample size.

Replication

For the Estonian Genome Centre Data, 32,594 individuals were genotyped on Illumina Global Screening (GSA) arrays and we imputed the data set to an Estonian reference, created from the whole genome sequence data of 2,244 participants\cite{est_data}. From 11,130,313 markers with imputation quality score > 0.3, we selected SNPs that overlapped with the UK Biobank, resulting in a set of 8,433,421 markers.

We selected height and BMI measures from the Estonian Genome Centre data, in 32,594 individuals genotyped on GSA array and converted them to sex-specific z-scores after applying the same outlier removal procedure as in UKB and adjusting for the age at agreement. Prevalent cases of CAD and T2D in the Estonian Biobank cohort were first identified on the basis of the baseline data collected at recruitment, where the

	information on prevalent diseases was either retrieved from medical records or self-reported by the participant. The cohort was subsequently linked to the Estonian Health Insurance database that provided additional information on prevalent cases (diagnoses confirmed before the date of recruitment) as well as on incident cases during the follow-up.<br><br>As the UK Biobank marker effects are estimated from traits that were standardized to a z-score prior to analysis, all effect sizes obtained are on the SD scale. Thus when we create a genomic predictor, for say coding SNPs, by multiplying SNPs mapped to coding regions genotyped in Estonia to the effect sizes obtained in the UK Biobank for each iteration, to obtain a genetic predictor for each iteration, providing a posterior predictive distribution that is also on the SD scale. For each trait, we created 2000 genomic predictors for each individual in the Estonian Biobank, at each of the 13 annotation groups, by selecting effect size estimates obtained every tenth iteration from the last 3000 iterations of each of the four Gibbs chains and combining them together in a single posterior.<br><br>We calculated prediction accuracy as the proportion of phenotypic variation explained by the genomic predictor, and area under the receiver operator curve (AUC) for T2D and CAD using each individual's mean genetic predictor. For each of the 13 annotation groups, we calculated the partial correlation of the genetic predictor of each of the 2000 iterations and the phenotype, and then used this to estimate the independent proportional contribution of each group to the total prediction accuracy, providing a metric of replication for our UK Biobank enrichment results. All of the genome-wide predictors provided significant prediction accuracy, replicating our results. We also replicated our findings for the regions which contribute the highest amount of variation for the four traits.
Randomization	This is a population-level study and thus individuals were not grouped into experimental groups. We controlled for a series of covariates. All phenotypes were adjusted for age of attending assessment centre (UKB code 21003-0.0, factor with levels for each age), year of birth (UKB field 34-0.0, factor with levels for each year), UK Biobank recruitment centre (UKB field 54-0.0, factor with levels for each centre), Genotype batch (UKB field 22000, factor with levels for each batch) and final 20 leading principal components of 1.2 million LD clumped markers from the 8,430,446 markers included in the analysis, calculated using flashPCA (see Code Availability). The residuals were then converted to z-scores with 0 mean and variance of 1. Similarly as for relatedness, population stratification is also accounted for within the BayesRR model through the addition of a background of marker effects entering the model, however we also wished to account for this in the standard manner by adjusting for the leading 20 PCs of the SNP data to get as close as possible to the inclusion of markers in the model that reflect LD with the causal variants. We note that as with any association model, while we take steps to adjust for known spatial (UKB centre), batch, and ancestry effects, and that the effects of each SNP is estimated jointly (and thus conditionally on the effects of all the other SNPs) environmentally induced covariance between SNP markers and a phenotype is still possible.
Blinding	This is a population-level study and thus blinding is not relevant here. The authors were not involved in the data collection. Individuals were invited to participate in the study.

Reporting for specific materials, systems and methods

We require information from authors about some types of materials, experimental systems and methods used in many studies. Here, indicate whether each material, system or method listed is relevant to your study. If you are not sure if a list item applies to your research, read the appropriate section before selecting a response.

Materials & experimental systems

n/a	Involved in the study
☒	☐ Antibodies
☒	☐ Eukaryotic cell lines
☒	☐ Palaeontology and archaeology
☒	☐ Animals and other organisms
☐	☒ Human research participants
☒	☐ Clinical data
☒	☐ Dual use research of concern

Methods

n/a	Involved in the study
☒	☐ ChIP-seq
☒	☐ Flow cytometry
☒	☐ MRI-based neuroimaging

Human research participants

Policy information about studies involving human research participants

Population characteristics	UK Biobank participants characteristics are publicly available here: https://biobank.ndph.ox.ac.uk/showcase/<br><br>Estonian Genome Centre Data characteristics are also publicly available here: https://biobank.ndph.ox.ac.uk/showcase/
Recruitment	Volunteer population-level studies.
Ethics oversight	UK Biobank has approval from the North West Multi-centre Research Ethics Committee (MREC) to obtain and disseminate data and samples from the participants (http://www.ukbiobank.ac.uk/ethics/), and these ethical regulations cover the work in this study. Written informed consent was obtained from all participants. Data from this project were held under UK Biobank project ID 35520.<br><br>All Estonian biobank participants have signed a broad informed consent form and the study was carried out under ethical approval 1.1-12/2856 from the Estonian Committee on Bioethics and Human Research (Estonian Ministry of Social Affairs).

Note that full information on the approval of the study protocol must also be provided in the manuscript.
